# The intrinsically disordered CARDs‐Helicase linker in RIG‐I is a molecular gate for RNA proofreading

**DOI:** 10.15252/embj.2021109782

**Published:** 2022-04-19

**Authors:** Brandon D Schweibenz, Swapnil C Devarkar, Mihai Solotchi, Candice Craig, Jie Zheng, Bruce D Pascal, Samantha Gokhale, Ping Xie, Patrick R Griffin, Smita S Patel

**Affiliations:** ^1^ Department of Biochemistry and Molecular Biology Robert Wood Johnson Medical School Rutgers University Piscataway NJ USA; ^2^ Graduate Program in Biochemistry Rutgers University Piscataway NJ USA; ^3^ Cell and Development Biology Rutgers University Piscataway NJ USA; ^4^ Department of Molecular Medicine The Scripps Research Institute Jupiter FL USA; ^5^ Department of Cell Biology and Neuroscience Rutgers University Piscataway NJ USA; ^6^ Cellular and Molecular Pharmacology Rutgers University Piscataway NJ USA; ^7^ Rutgers Cancer Institute of New Jersey New Brunswick NJ USA; ^8^ Department of Integrative Structural and Computational Biology Jupiter FL USA

**Keywords:** intrinsically disordered linker, regulatory region, RIG‐I, RNA discrimination, self‐vs‐non‐self, Immunology

## Abstract

The innate immune receptor RIG‐I provides a first line of defense against viral infections. Viral RNAs are recognized by RIG‐I's C‐terminal domain (CTD), but the RNA must engage the helicase domain to release the signaling CARD (Caspase Activation and Recruitment Domain) domains from their autoinhibitory CARD2:Hel2i interactions. Because the helicase itself lacks RNA specificity, mechanisms to proofread RNAs entering the helicase domain must exist. Although such mechanisms would be crucial in preventing aberrant immune responses by non‐specific RNAs, they remain largely uncharacterized to date. This study reveals a previously unknown proofreading mechanism through which RIG‐I ensures that the helicase engages RNAs explicitly recognized by the CTD. A crucial part of this mechanism involves the intrinsically disordered CARDs‐Helicase Linker (CHL), which connects the CARDs to the helicase subdomain Hel1. CHL uses its negatively charged regions to antagonize incoming RNAs electrostatically. In addition to this RNA gating function, CHL is essential for stabilization of the CARD2:Hel2i interface. Overall, we uncover that the CHL and CARD2:Hel2i interface work together to establish a tunable gating mechanism that allows CTD‐chosen RNAs to bind the helicase domain, while at the same time blocking non‐specific RNAs. These findings also indicate that CHL could represent a novel target for RIG‐I‐based therapeutics.

## Graphical abstract

Specificity for RNA‐binding mediated activation of RIG‐I is ensured by a gating mechanism involving the CHL and CARD:Hel2i interface.

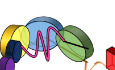

## Introduction

RIG‐I (Retinoic Acid Inducible Gene‐I) is an innate immune receptor responsible for surveilling the cytoplasm for viral RNAs (Yoneyama *et al*, 2005). RIG‐I recognizes short blunt‐ended double‐stranded (ds) RNAs with 5’‐triphosphate (5'ppp), 5’‐diphosphate (5'pp), and 5’‐m7G cap as PAMPs (pathogen‐associated molecular pattern) (Jiang *et al*, 2011; Goubau *et al*, 2014; Schuberth‐Wagner *et al*, 2015; Devarkar *et al*, 2016). Such PAMP features are not present in endogenous RNAs but found in many viral RNA genomes and most replication intermediates of negative‐strand and positive‐strand RNA viruses (Stumper *et al*, 2005; Kato *et al*, 2006; Rehwinkel *et al*, 2010; Schuberth‐Wagner *et al*, 2015; Devarkar *et al*, 2016; Hu *et al*, 2017). Upon recognizing viral RNA PAMPs, RIG‐I initiates a signaling cascade that culminates in type I interferon response, efficiently controlling viral infections (Yoneyama *et al*, 2004; Stumper *et al*, 2005; Poeck *et al*, 2010).

**Figure 1:**
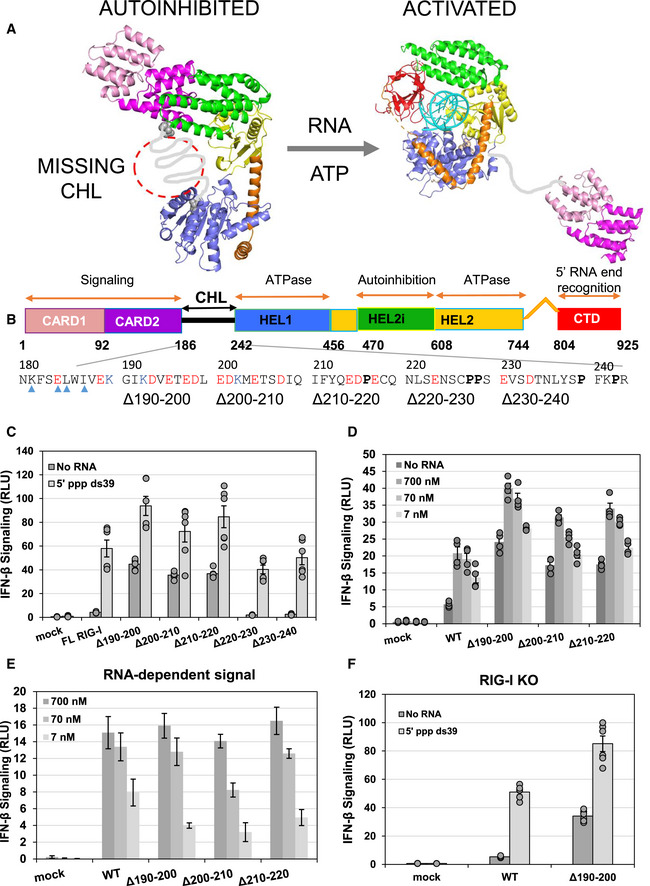
The CARDs‐Helicase Linker (CHL) regulates RIG‐I signaling In the autoinhibitory state of RIG‐I, the helicase is in an open conformation and the CARDs are interacting with Hel2i. The missing CHL, in gray, passes near the RNA binding groove of the helicase domain as its spans the 40 Å distance between the C terminus of CARD2 and N terminus of Hel1. PAMP RNA and ATP binding induces a conformational change to close the helicase subdomains around the ligands and release the CHL and CARDs. Models were generated from crystal structures (PDB ID: 4A2W, left; composite model of two crystal structures PDB IDs: 4NQK and 5E3H, right and colored as in (B)).Domain schematic of RIG‐I with the CHL sequence highlighted. Blue arrows, CARD2 residues known to contact the Hel2i domain in the CARD2:Hel2i interface in autoinhibited duck RIG‐I. Negative and positive charged amino acids are labeled red and blue, respectively. Proline residues are in bold.IFN response of mock‐transfected, WT and CHL RIG‐I mutants measured by luciferase reporter assays in HEK293T cells under No RNA or 50 nM 5’ppp ds39 transfected conditions and reported as Relative Luciferase Units (RLU). Each condition was performed three times by two independent workers (*n* = 6), and individual trials are plotted. Bars represent mean value, and error bars reflect standard error.Dose‐titration IFN response of mock‐transfected, WT, and constitutively active CHL RIG‐I mutants measured by luciferase reporter assays in HEK293T cells. Cells were transfected with either no RNA or 700 nM, 70 nM, or 7 nM of 5’ ppp ds39 RNA. Each condition was performed two times by two independent workers (*n* = 4). Bars represent mean value, and error bars reflect standard error.5’ppp ds39 stimulated IFN response of mock, WT, and constitutively active CHL RIG‐I mutants. Each total IFN response in (D) was subtracted by each plasmid’s no RNA transfection condition to show the amount of IFN response explicitly due to transfected RNA. Bars represent the average IFN response of each RNA condition minus the average no RNA IFN response, and error bars represent the average standard error of the two.IFN response assay of mock, WT, and Δ190–200 RIG‐I as in (C) except the assay was performed in HEK293T RIG‐I KO cells. Each condition was performed three times by two independent workers (*n* = 6), and individual trials are plotted. Bars represent mean value, and error bars reflect standard error.

Structural studies show that RIG‐I has flexibly linked domains that can switch between active and inactive states to respond appropriately to viral RNAs (Fig 1A) (Civril *et al*, 2011; Jiang *et al*, 2011; Kowalinski *et al*, 2011; Luo *et al*, 2011, 2012; Devarkar *et al*, 2016). The core helicase domain contains two helicase subdomains, Hel1 and Hel2, and a nested Hel2i, flanked by a tandem array of signaling domain CARDs (Caspase Activation and Recruitment Domains) and a PAMP recognition C‐terminal domain (CTD). When RIG‐I is not bound to RNA, the helicase domain is in an open, ATPase‐inactive conformation. In this conformation, the CARDs are sequestered by Hel2i through CARD2:Hel2i interactions and inactive in signaling (Fig 1A). When RIG‐I fully engages the RNA in a closed helicase conformation, the induced conformational changes activate the ATPase and disrupt the CARD2:Hel2i interface to release the CARDs from their autoinhibitory interactions (Zheng *et al*, 2015, 2018; Dickey *et al*, 2019). The exposed CARDs can initiate an immune response by interacting with downstream adapter proteins (Peisley *et al*, 2014; Wu *et al*, 2014).

RIG‐I must be activated by viral RNAs and not self RNAs that are abundant in the cytoplasm. Aberrant immune responses initiated by RIG‐I are harmful and lead to autoinflammatory disorders (Roers *et al*, 2016; Crowl *et al*, 2017). RIG‐I has evolved several mechanisms to discriminate self and non‐self. PAMP RNAs are explicitly recognized by the CTD of RIG‐I, which provides the first layer of RNA proofreading (Cui *et al*, 2008; Lu *et al*, 2010; Wang *et al*, 2010; Vela *et al*, 2012; Ramanathan *et al*, 2016). RIG‐I then utilizes its ATPase activity to power translocation along the RNA, and dissociation from non‐PAMP RNAs (Devarkar *et al*, 2018). However, to activate the signaling CARDs, the chosen RNA must be loaded into the helicase domain. Unlike CTD, which specifically recognizes blunt 5’ tri‐ or diphosphorylated RNA ends, the helicase domain generally interacts with the phosphodiester backbone (Jiang *et al*, 2011; Kowalinski *et al*, 2011; Luo *et al*, 2011). Hence, regulatory mechanisms must exist to selectively load CTD‐chosen RNAs into the helicase domain and filter out non‐specific RNAs. However, we do not know how RNA binding into the helicase domain is regulated. Previous studies suggested that the CARD2:Hel2i interface plays a role in gating the helicase (Vela *et al*, 2012; Ramanathan *et al*, 2016), but the underlying mechanisms were not known.

In this study, we have discovered a new regulatory region that controls RNA binding into the helicase domain. This finding was made serendipitously while studying the role of the ~56 aa linker that connects the CARDs to Hel1, referred to here as CHL (CARDs‐Helicase Linker). No known functions were associated with the CHL, except that it tethers the CARDs to the helicase and that appropriately long CHL might be important for enabling intermolecular CARDs:CARDs interactions. Therefore, we were surprised to find that small deletions in CHL did not impair RIG‐I signaling and instead constitutively activated RIG‐I signaling without the addition of PAMP RNA. These results suggest that CHL is not a passive linker but a regulatory region that keeps RIG‐I in the autoinhibitory state in the absence of PAMP RNA. To understand the role of CHL, we carried out a detailed mechanistic study of the CHL mutants using a combination of cellular, biochemical, and biophysical methods, including hydrogen–deuterium exchange and mass spectrometry (HDX‐MS), equilibrium RNA binding, and stopped‐flow kinetics. These studies revealed two essential roles of the CHL. One role of the CHL is to stabilize the CARD2:Hel2i interface and maintain RIG‐I in an autoinhibitory conformation in the absence of PAMP RNA. The second role of CHL is to shield the helicase domain and antagonize non‐specific RNAs. Both functions of the CHL depend on its negatively charged amino acids acting as an electrostatic gate. The RNA proofreading function of the CHL is greatly enhanced by a stable CARD2:Hel2i interface. The two work synergistically to minimize erroneous RNA binding events, ensuring that RNAs chosen by the CTD are specifically loaded into the helicase domain. The newly discovered regulatory functions of the CHL can be potentially leveraged in RIG‐I‐based therapeutics.

## Results

### CHL deletions constitutively activate RIG‐I signaling in the absence of PAMP RNA

**Figure EV1:**
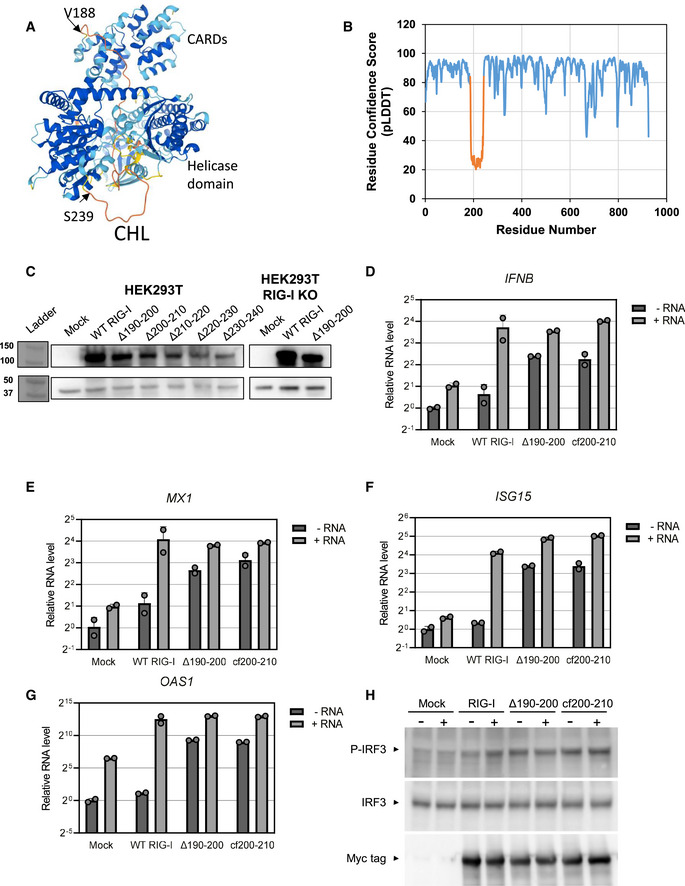
Induction of the IFN response genes by the CHL mutants AAlphaFold predicted structure of RIG‐I shows CHL bound in the RNA binding pocket of the helicase domain. The colors correspond to model per‐residue confidence (pLDDT): Dark blue, very high (pLDDT > 90), light blue, confident (90 > pLDDT > 70), yellow, low (70 > pLDDT > 50), orange, very low (pLDDT < 50). Regions below 50 pLDDT are predicted to be unstructured, like the CHL in orange.BGraph demonstrating per‐residue confidence (pLDDT) of AlphaFold prediction shown in (A), indicating low‐confidence in the prediction of CHL structure. The CHL (186–241) is highlighted in orange.CWestern Blot confirms RIG‐I expression in the reporter assays in Fig 1. In each experiment, pcDNA3.1 myc‐tagged RIG‐I constructs (approximately 108 kDa) were recognized with a primary α‐Myc antibody. β‐actin (approximately 42 kDa) was used as a normalization control. Numbers (left) refer to molecular weight in kDa. Note that HEK293T and HEK293T RIG‐I KO Western blots were performed on separate gels.D–GqRT–PCR assays show the induction of antiviral IFN response genes in the absence and presence of PAMP RNA, 5’ppp ds39. Note the Y‐axis is in log scale. Each bar represents the mean ± SD. Each point represents a mechanical replicate (*n* = 2).HWestern blot to show induction of pIRF3 in RIG‐I and RIG‐I mutant transfected cells.

The negatively charged CHL (186–241 aa) is predicted to be intrinsically disordered (Oates *et al*, 2013), and accordingly, not resolved in the autoinhibited duck RIG‐I structure (Kowalinski *et al*, 2011) (Fig 1A). The AlphaFold structure also predicts that the CHL is disordered, demonstrated by a low confidence score and random coil conformation modeling (Jumper *et al*, 2021) (Fig EV1A and B). Additionally, the crystal structure of RIG‐I suggests that the missing CHL is in close proximity to the helicase domain. Interestingly, AlphaFold structure prediction places the unstructured CHL within the RNA binding channel of the helicase domain (Fig EV1A). To investigate the role of CHL in RIG‐I signaling, we made segmental deletions of 11 amino acids in the 190–240 region (Fig 1B) and tested the mutants in cell signaling assays. WT RIG‐I and CHL mutants were transfected in HEK293T cells and their interferon response was measured using a dual‐luciferase IFN‐β promoter‐reporter assay with and without PAMP RNA. As expected, in the absence of PAMP RNA, WT RIG‐I showed minimal basal signaling response, but unexpectedly, three of the five CHL mutants showed close to 10‐fold higher basal signaling response than WT RIG‐I (Fig 1C). We verified that all the RIG‐I proteins were expressed after transfection in the 293T cells (Fig EV1C). The CHL mutants that were constitutively activated had deleted regions close to CARD2 (Δ190–200, Δ200–210, and Δ210–220), whereas mutants with deletions close to Hel1 (Δ220–230 and Δ230–240) behaved like WT RIG‐I and did not signal well in absence of PAMP RNA. The results indicate that the 190–220 region of the CHL is involved in autoinhibiting RIG‐I signaling in the absence of PAMP RNA. The 220–240 region may have a different role.

When the cells were transfected with PAMP RNA, 5’ppp ds39, all tested RIG‐I constructs responded with increased signaling (Fig 1C). To test the relative sensitivities of the wild‐type RIG‐I and the constitutively active CHL mutants to activation by the PAMP RNA, we titrated the cells with 7–700 nM PAMP RNA and measured the signaling responses (Fig 1D). The CHL mutants had an overall higher interferon signaling activity than wild‐type RIG‐I at all RNA concentrations. After subtracting the basal signaling response, we find that the PAMP signaling response of the CHL mutants at the lowest 7 nM PAMP RNA concentration is about 2‐fold lower than wild‐type RIG‐I (1.7‐fold decrease for WT, while CHL mutants decreased between 2.5‐fold and 3.5‐fold) (Fig 1E). The signaling response of the CHL mutants at intermediate or high RNA concentrations was comparable to wild‐type RIG‐I. Thus, CHL deletions only slightly impairs the RNA‐dependent signaling activity relative to wild‐type RIG‐I at low PAMP RNA concentrations.

To confirm that the signaling activity measured in HEK293T cells is not due to activation of endogenous RIG‐I, we tested a minimal CHL mutant panel in HEK293T RIG‐I KO cells (Fig 1F). Overall, we obtained the same results in RIG‐I KO and 293T cells. The Δ190–200 RIG‐I showed a 7‐fold higher basal signaling activity in the RIG‐I KO cells, which is similar to the 10‐fold observed in the 293T cells. This confirms that the CHL mutants are constitutively activated in interferon signaling.

To demonstrate that the constitutively active CHL mutant activates the antiviral interferon (IFN) response pathway, we measured the expression of several IFN response genes, including IFNB, MX1, ISG15, OAS1 using qRT–PCR in the absence and presence of 5’ppp ds39 PAMP RNA. The Δ190–200 RIG‐I activated the IFN response genes and pIRF3 expression in the absence of PAMP RNA, whereas WT RIG‐I required PAMP RNA for activation of these genes (Fig EV1D–H). These cellular studies demonstrate that CHL is a critical regulatory region that controls the RIG‐I signaling pathway inducing the IFN response genes.

### CHL stabilizes the CARD2:Hel2i interface to minimize CARDs exposure and prevent non‐specific RNA binding

Small deletions in CHL can increase the basal signaling activity of RIG‐I in two ways. First, the deletions may destabilize the autoinhibited conformation of RIG‐I and spontaneously expose the CARDs independently of RNA binding. Additionally, the deletions may dysregulate RNA binding, promoting CARDs activation through promiscuous binding of self RNAs.

**Figure 2:**
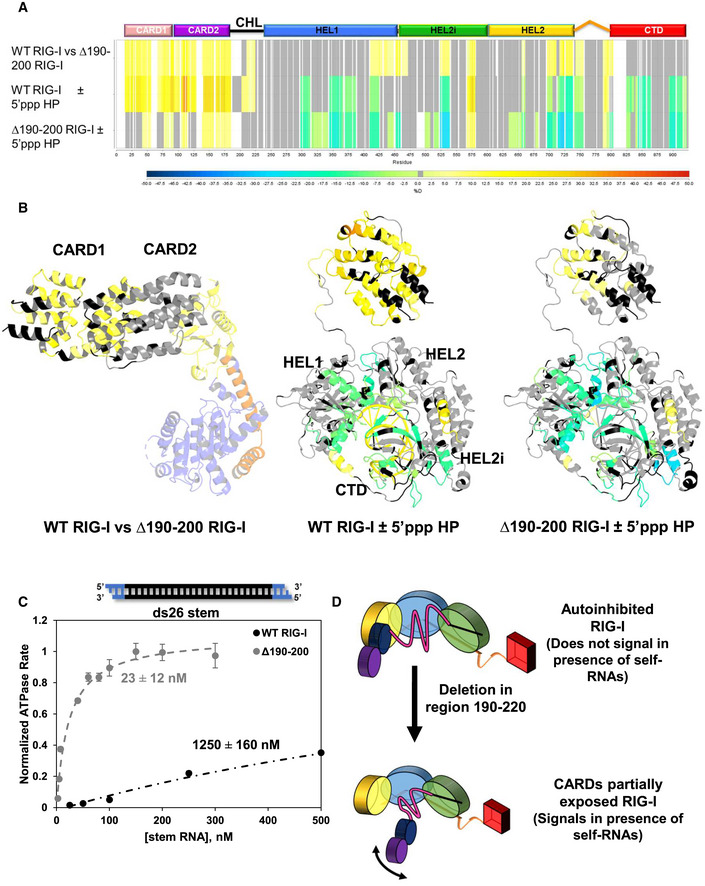
CHL stabilizes CARDs in the autoinhibitory conformation and inhibits stem RNA binding Hydrogen–Deuterium Exchange Mass Spectroscopy (HDX) heatmap of three comparisons: WT RIG‐I vs Δ190–200 RIG‐I without RNA; WT RIG‐I with and without 5’‐triphosphate (5’ppp) RNA, a RIG‐I PAMP; and Δ190–200 RIG‐I with and without 5’ppp RNA. The bar below indicates % deuteration of a given peptide region. White spaces indicate no sequence coverage, and grey represents regions in which the coverage was non‐significant.Data from (A) modeled onto autoinhibited RIG‐I (PDB ID: 4A2W) or activated RIG‐I, which is a composite model of two crystal structures (PDB IDs: 5E3H and 4NQK). In the autoinhibited structure, only CARDs and Hel2i are colored according to the HDX results.RIG‐I binding to ds26 stem RNA. The ATPase activity of WT RIG‐I and Δ190–200 RIG‐I was measured at increasing concentration of ds26 stem RNA (black: RNA, blue: DNA). The binding curves were fit using a hyperbola (Equation 5) to obtain the stem RNA K_D,app_ for each RIG‐I construct, as shown. The mean value is shown as a bar; error bars denote standard error. For each point, standard error was determined by the fit to each point’s time course (mechanical replicates, *n* = 3).Model showing the consequences of CHL deletion. Small deletions in the CHL disrupt both the CHL positioning and the autoinhibitory CARD2:Hel2i interface, causing aberrant CARDs exposure and stem RNA binding. The coloring of the helicase subdomains, CTD, and CARDs is the same as in Fig 1.

**Figure EV2:**
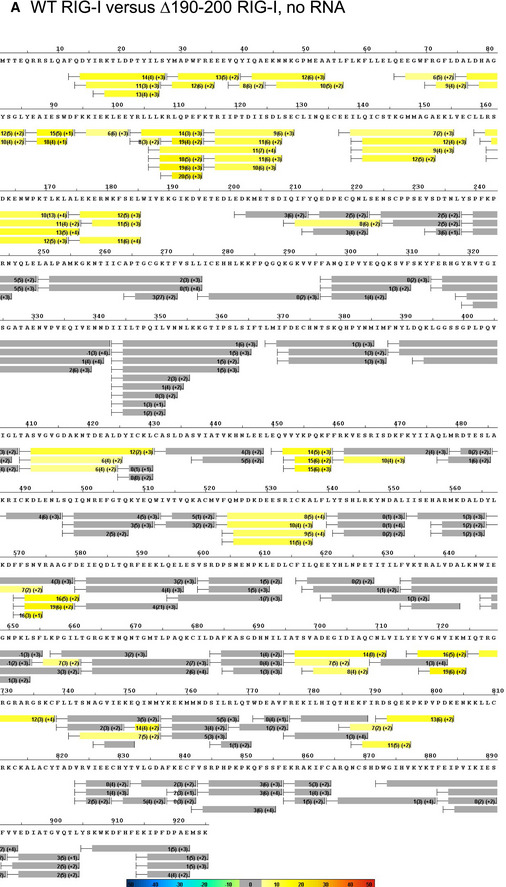
Hydrogen–Deuterium Exchange Mass Spectroscopy (HDX) heatmap comparing WT RIG‐I and Δ190–200 RIG‐I The average ΔD_2_O% ± standard deviation between WT RIG‐I and Δ190–200 RIG‐I across all HDX time points. HDX Workbench colors each peptide according to the smooth color gradient HDX perturbation key shown in each indicated figure. Average ΔD_2_O% between −5% and 5% are considered non‐significant and are colored gray.

To test the first possibility, we used differential HDX‐MS analysis, a powerful technique to monitor CARDs exposure in RIG‐I used previously (Zheng *et al*, 2015, 2018, 2019). The solvent exchange rates were significantly different between WT and Δ190–200 RIG‐I in the absence of RNA. The CHL mutant showed a higher solvent exchange rate in many regions, including the CARDs (aa 1–186), Hel1 (aa 410–460), Hel2 (aa 695–756, 785–803), and Hel2i (aa 566–574 and 522–539) (Figs 2A and B, and EV2). This indicates that 190–200 deletion has a global effect on the autoinhibited conformation of RIG‐I. In particular, increased solvent exchange at the CARD2:Hel2i interface, comprising the CARD2 latch peptide (aa 103–114) and the interacting Hel2i regions (aa 566–574 and 522–539) suggests this global effect is caused by the 190–200 deletion through destabilization of the CARD2:Hel2i interface.

Next, we compared the solvent exchange rates in the absence and presence of 5’ppp ds10 hairpin, which stimulates CARDs release in WT RIG‐I (Fig 2A and B, Appendix Figs S1 and S2). CARDs exposure was more prominent in WT RIG‐I than in Δ190–200 RIG‐I. This is understandable because the Δ190–200 RIG‐I CARDs are already partially exposed before adding RNA. Differential HDX‐MS also shows that RNA binding regions are protected in WT and Δ190–200 RIG‐I to a similar extent. Hence, the CHL mutant is not defective in RNA binding, consistent with the increased signaling with PAMP RNA in the above assays. The most significant finding from the HDX‐MS experiments is that CHL has a role in stabilizing RIG‐I CARDs in the autoinhibitory conformation in the absence of RNA.

To investigate if partial CARDs exposure dysregulates RNA binding, we measured the affinity of WT and Δ190–200 RIG‐I for a stem RNA mimic. The ds26 stem was designed to contain a 17 bp dsRNA region flanked by 4 bp of dsDNA ends. The DNA ends block RIG‐I from end binding, forcing it to bind internally in the RNA stem region, mimicking secondary stem structures in self RNAs. RNA K_D,app_ values were determined from titration experiments monitoring RIG‐I's RNA‐dependent ATPase activity as a function of increasing RNA. As expected, WT RIG‐I binds to ds26 stem with a very weak affinity and K_D,app_ ~1250 nM (Fig 2C). Interestingly, Δ190–200 RIG‐I showed a much higher affinity and K_D,app_ of ~20 nM, which is 50‐fold tighter than WT RIG‐I. As shown (Fig 1D), it appears that a small deletion in CHL in the 190–220 region exposes the CARDs and disrupts the positioning of CHL to enable stem RNA to bind into the helicase domain. These results indicate that the hyperactive signaling response of the CHL mutants could be both due to partially exposed CARDs and promiscuous binding and activation by self RNAs in the cytoplasm. Thus, CHL has a dual role—stabilize the CARDs in the autoinhibitory conformation and regulate RNA binding into the helicase domain.

### The negative charges in the CHL are essential in autoinhibiting aberrant RIG‐I signaling

How does the deletion of 11 aa in CHL destabilize the CARD2:Hel2i interface? Is it a length effect, or does the deletion disrupt some autoinhibitory interactions of the CHL with the helicase? An unstructured CHL of 56 aa should be theoretically 168 Å long (assuming ~3 Å per aa), which is four times longer than the distance it needs to span between CARD2 and Hel1 (~40 Å). A 33 Å deletion is not long enough to shorten the linker and disrupt the interface if the CHL is truly unstructured, as the Alphfold structure prediction indicates (Fig EV1A and B). Thus, CHL may have specific, dynamic interactions with the helicase domain that stabilizes the autoinhibitory CARDs conformation.

**Figure 3:**
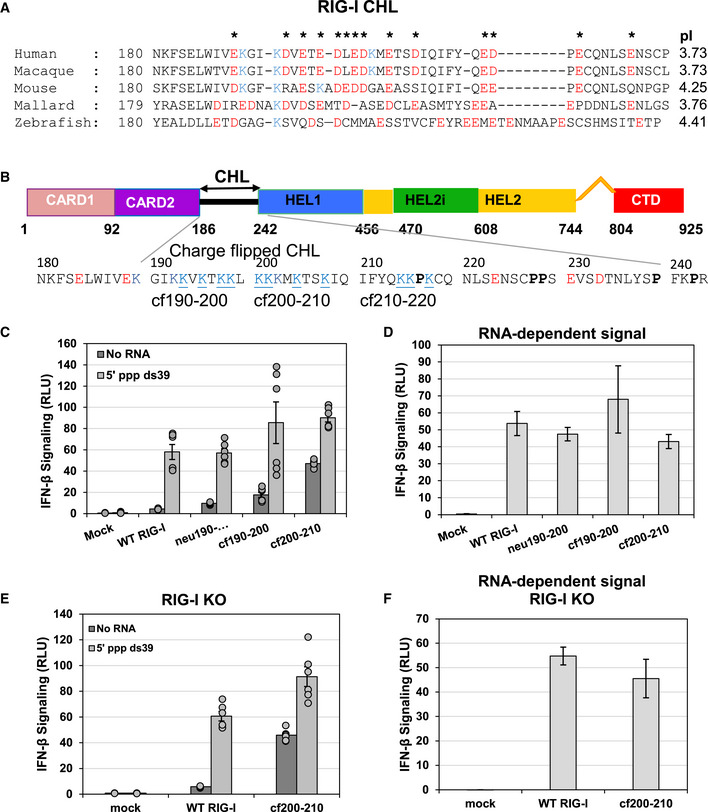
The CHL’s regulatory ability is predicated on its negatively charged amino acids Alignment of RIG‐I homolog sequences spanning human to bony fish from residues 180–228 shows the conserved negatively charged amino acids in the CHL of all species. Note, this CHL region 190–220 was identified in Fig 1 as important for regulation. All glutamate and aspartate residues are colored red, and lysines are colored blue. Asterisks indicate when a negative charge, either glutamate or aspartate, is conserved at that position in three out of five homologs tested. pI was calculated for each RIG‐I homolog region aligning with human RIG‐I amino acids 186–241.RIG‐I domain schematic with the sequence of CHL highlighted. The sequences of the charge flip mutants (cf190–200, cf200–210, cf210–220) are shown.IFN response measured by reporter assays in HEK293T cells in Mock‐transfected, WT RIG‐I, a glycine‐serine‐threonine repeat substitution CHL mutant (neu190–200), and CHL charge flip (cf) mutants under No RNA conditions or 50 nM 5’ ppp ds39 RNA transfected conditions and reported as Relative Luciferase Units (RLU). Each condition was performed three times by two independent workers (*n* = 6), and individual trials are plotted. Bars represent mean value, and error bars reflect standard error.5’ppp ds39 stimulated IFN response. No RNA signal from panel C was subtracted from the total amount of signaling after RNA transfection. Bars represent each plasmid’s average 50 nM 5’ ppp ds39 RNA IFN response minus the average no RNA IFN response, while the error bars represent the average standard errors of each construct with and without RNA transfection.IFN response of Mock, WT RIG‐I, and cf200–210 RIG‐I performed as in (C) in HEK293T RIG‐I KO cells. Each condition was performed three times by two independent workers (*n* = 6), and individual trails are plotted. Bars represent mean value, and error bars reflect standard error.5’ppp ds39 stimulated IFN response of Mock, WT RIG‐I, and cf200–210 RIG‐I as in (D) in HEK293T RIG‐I KO cells. Each total IFN response in (E) was subtracted by each plasmid’s no RNA transfection condition to show the amount of IFN response explicitly due to transfected RNA. Bars represent the average IFN response of each RNA condition minus the average no RNA IFN response, and error bars represent the average standard error of the two.

**Figure EV3:**
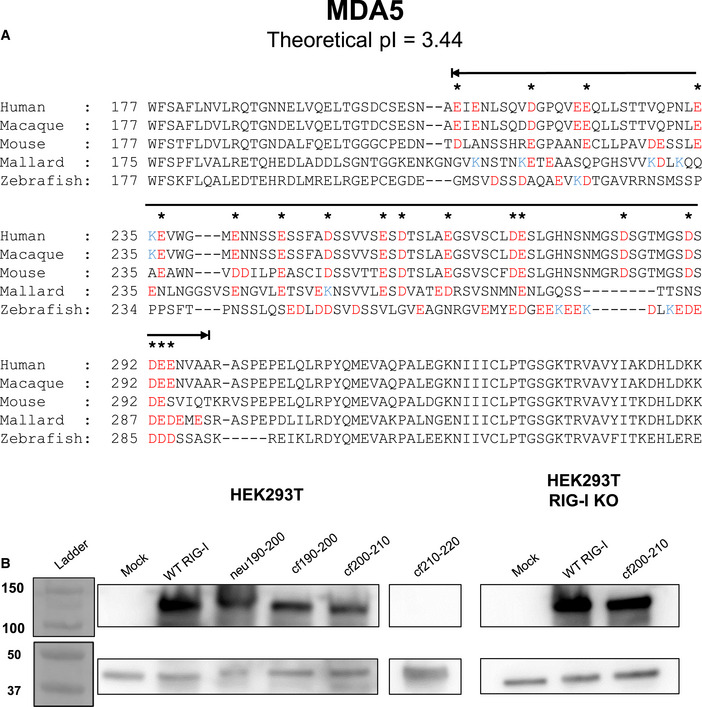
CHL in MDA5 and Western analysis of RIG‐I expression in the reporter assays of the charge flip CHL mutants RIG‐I Like Receptor MDA5 has an electronegative CARD2‐helicase linker region similar to RIG‐I. MDA5 sequences from five homologs spanning human to bony fish were aligned and analyzed as in Fig 3A. Black line above the sequence indicates the MDA5 CHL. All glutamate and aspartate residues are colored red, and lysines are colored blue. Asterisks indicate when a negative charge, either glutamate or aspartate, is conserved at that position in three out of five homologs tested.Western blot of signaling data shown in Fig 3 confirm protein expression, as in Fig EV1. In each experiment, pcDNA3.1 myc‐tagged RIG‐I constructs (approximately 108 kDa) were recognized with a primary α‐Myc antibody. β‐actin (approximately 42 kDa) was used as a normalization control. Cf210–220 was not included in cell signaling data as it did not express. Numbers (left) refer to molecular weight in kDa.

Sequence analysis of several RIG‐I homologs from bony fish to mammals shows that CHL is highly negatively charged. Although the CHL sequence conservation is poor relative to the helicase domain, the negative charges in CHL are well conserved (Fig 3A). Between residues, 186–220, 11 of the 35 amino acids of the human CHL are either aspartate or glutamate, with only three lysine residues across the CHL. The entire CHL region from 186–241 has a theoretical pI of 3.73. RIG‐I homologs ranging from monkeys to zebrafish similarly contain negatively charged CHLs, with predicted pI’s ranging from 3.73 to 4.41. This pattern further extends to RIG‐I‐like receptor MDA5, whose larger CHL region has a similarly conserved, negative charge pattern (Fig EV3A). The negative charges in CHL may interact with the positively charged RNA binding pocket of the helicase domain, as predicted by AlphaFold (Fig EV1A), to stabilize the autoinhibitory conformation.

To determine if CHL's negatively charged regions are involved in the autoinhibitory mechanism, we systematically introduced clusters of neutral or positively charged amino acids in the 190–220 region. We chose this region because deletions in the 190–220 region aberrantly activated RIG‐I signaling in above signaling experiments. The 190–200 segment was replaced with a glycine‐serine‐threonine repeat to make the neu190–200 mutant, mimicking a more neutral, potentially less structured region. To create positively charged segments, the negative aspartate and glutamate residues were charged flipped to lysine in cf190–200, cf200–210, and cf210–220 (Fig 3B). The signaling activities of these CHL mutants were compared to WT RIG‐I using the reporter assay in HEK293T cells. All RIG‐I proteins, except cf210–220, were expressed to similar levels in the cells (Fig EV3B). For unknown reasons, cf210–220 mutant was not expressed. Changing the acidic 190–200 segment to a neutral one increased the basal signaling activity by 2–3 fold and charge flipping by 5‐fold (Fig 3C). Strikingly, charge flipping the 200–210 segment (cf200–210) increased the signaling response by 13‐fold. Thus, charge flipping has a similar effect of activating RIG‐I signaling as the 11 aa deletion. The CHL mutants were also activated by 5’ppp ds39 RNA. Subtracting the basal signaling response from the total shows that PAMP RNA stimulates the CHL mutants to a similar level as WT RIG‐I at this intermediate RNA concentration, like the deletion mutants (Fig 3D). The above results support the hypothesis that the negative charges in the 190–210 CHL region are involved in some specific interactions with the helicase domain, and deletion or charge flipping disrupts these interactions to destabilize the CARD2:Hel2i interface, without impairing the PAMP RNA stimulated response.

Cell signaling assays in 293T RIG‐I KO cells showed a similarly high basal signaling response from the cf200–210 RIG‐I, confirming that endogenous RIG‐I activation was not responsible for the aberrant signaling in the absence of PAMP RNA (Fig 3E and F). The cf200–210 RIG‐I mutants also induced the IFN response genes and expressed pIRF3 in the absence of PAMP RNA, whereas this response was observed in WT RIG‐I only in the presence of PAMP RNA (Fig EV1D–H). Based on these studies, we conclude that the negatively charged amino acids in the 190–210 region in CHL are involved in regulating the autoinhibitory conformation of RIG‐I. The loss of some key interactions involving the negative charges in CHL, rather than CHL shortening alone, is responsible for CARDs exposure.

### CHL's negative charges compete with RNA to block stem RNA binding into the helicase domain

**Figure 4:**
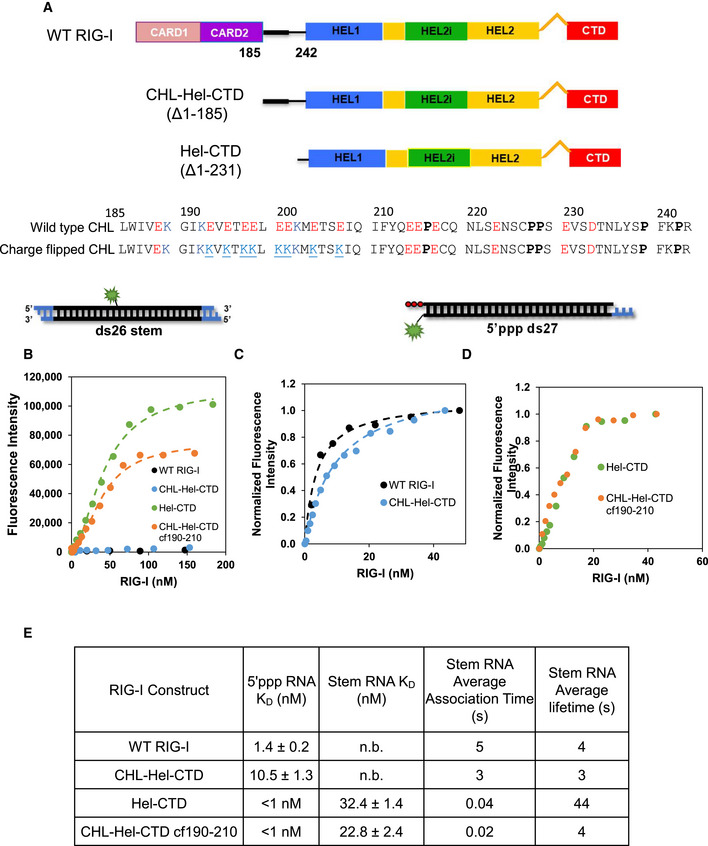
Negative charges in the CHL are important for inhibiting stem RNA binding Schematics of the tested RIG‐I constructs. The 190–210 CHL is highlighted by a thicker black bar. The negative amino acids are in red and positive ones in blue. The negatively charged amino acids in the 190–210 region were mutated to lysines to create the CHL‐Hel‐CTD cf190–210 construct. This change resulted in a pI change in the CHL from to 3.7 to 9.5.Fluorimetric titrations show ds26 stem RNA (Cy3 fluorophore at the 13^th^ position) binding to the various RIG‐I constructs. The RNA binding data of Hel‐CTD and CHL‐Hel‐CTD cf190–210 were fit using a Hill equation (Hel‐CTD, *n* = 1.8 ± 0.2; CHL‐Hel‐CTD cf190–210 *n* = 1.9 ± 0.1). Because WT RIG‐I and CHL‐Hel‐CTD did not finish binding within 400 nM, they could not be normalized.Fluorimetric titrations show 5’ppp ds27 (DY547 at 3’ position distal to the 3nt DNA overhang) binding to WT RIG‐I and CHL‐Hel‐CTD. The fluorescence intensity changes were normalized to the highest fluorescence in each RNA. The data were fit using a hyperbola.Fluorimetric titrations show 5’ppp ds27 binding to Hel‐CTD and CHL‐Hel‐CTD cf190–210. Both proteins bind 5’ppp RNA stoichiometrically.Table summarizes the RNA K_D_ values and shows the association and dissociation times of each RIG‐I construct. No binding or *n*. b. indicates that stem RNA binding was not observed within 400 nM of titrated RIG‐I. Each K_D_ value was repeated twice (biological replicate, *n* = 2) The average stem RNA association and dissociation times of the various RIG‐I constructs from stopped‐flow experiments (Fig EV4 and Appendix Table S2).

Above results indicate that CHL inhibits stem RNA binding. Does CHL regulate stem RNA binding on its own, and if so, are the negatively charged amino acids involved in RNA regulation? To address these questions, we purified four RIG‐I constructs: WT RIG‐I, CHL‐Hel‐CTD (lacks CARDs but retains CHL), Hel‐CTD (lacks CARDs and CHL), and CHL‐Hel‐CTD cf190–210 (negatively charged residues between 190–210 mutated into lysines) (Fig 4A and Appendix Fig S3A). Comparison of the stem RNA K_D_ of CHL‐Hel‐CTD to Hel‐CTD allowed us to determine whether CHL regulates RNA binding independently of maintaining the CARD2:Hel2i interface. Furthermore, comparing CHL‐Hel‐CTD and cf190–210 CHL‐Hel‐CTD allowed us to determine whether CHL's function is related to its exceptional negative charge.

Fluorescence‐intensity‐based titrations measured the affinity of the Cy3‐labeled ds26 stem RNA to the various RIG‐I constructs. Hel‐CTD binds the stem RNA with a high affinity, but CHL‐Hel‐CTD and WT RIG‐I showed no RNA binding under the same conditions (Fig 4B). The striking difference in the RNA binding property of CHL‐Hel‐CTD compared to Hel‐CTD demonstrates that CHL on its own can inhibit stem RNA binding. Interestingly, cf190–210 binds to stem RNA with a high affinity, like Hel‐CTD which lacks the CHL altogether. This clearly shows that the negative charges in CHL are involved in regulating RNA binding into the helicase domain.

All the RIG‐I constructs were competent in binding to 5’ppp ds27, PAMP RNA. The CHL‐Hel‐CTD binds to PAMP RNA with nanomolar K_D_, like WT RIG‐I (Fig 4C). Both cf190–210 and Hel‐CTD bind PAMP RNA stoichiometrically under the conditions of the experiments, so we could not accurately estimate their RNA K_D_ values (Fig 4D). Our EMSA experiments show that the CHL mutants, including the ∆190–200 RIG‐I, can oligomerize on PAMP RNA in the presence of ATP, like WT RIG‐I (Appendix Fig S3B and C). Thus, small deletions or mutations in CHL do not affect RNA binding and protein oligomerization on PAMP RNA.

**Figure EV4:**
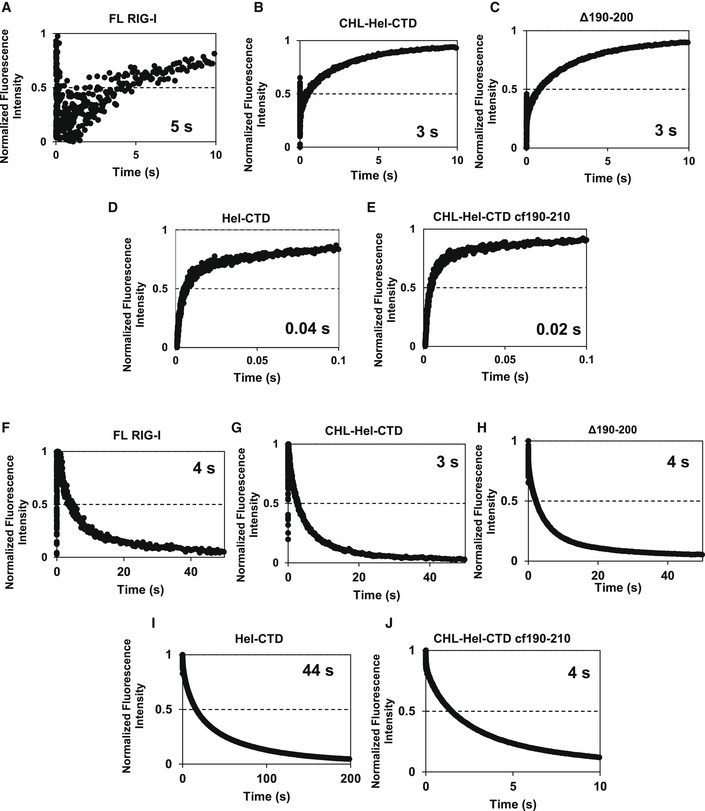
Stopped‐flow kinetic traces of association and dissociation reactions of ds26 stem with various RIG‐I constructs A–EThumbprint graphs showing ds26 stem RNA association kinetics to various RIG‐I constructs. Each curve is an average of at least 6 individual traces (*n* = 6).F–JThumbprint graphs showing ds26 stem RNA dissociation kinetics from various RIG‐I constructs. Numbers are the average association or dissociation times calculated from the amplitude and rate constants shown in Appendix Table S2 using Equation 3. Each curve is an average of at least six individual traces (*n* = 6).

What mechanism does CHL use to inhibit stem RNA binding? CHL can shield the helicase domain using its negatively charges and block RNA access, or CHL can actively destabilize the RNA complex to dissociate the stem RNA. We measured the association and dissociation kinetics of the ds26 stem RNA using stopped‐flow experiments to explore these possibilities. RIG‐I CTD normally has a diffusion‐limited on rate to RNA ends that dominates the association rate measurements (Devarkar *et al*, 2018). Stem ds26 lacks exposed RNA ends; therefore, the kinetics of stem RNA are dominated by the helicase domain rather than CTD. The association kinetics show a 75–125 fold slower rate of stem RNA binding to CHL‐Hel‐CTD and WT RIG‐I than to Hel‐CTD (Figs 4E and EV4, Appendix Table S1A). Thus, having CHL slows down stem RNA binding quite significantly, which is consistent with CHL’s role in shielding the helicase domain and preventing RNA access. The charge flipped CHL‐Hel‐CTD binds to stem RNA with a 2‐fold faster rate than Hel‐CTD, demonstrating that the CHL’s negative charge is important for its ability to shield the helicase domain. We conclude that CHL is an electrostatic gate that uses its negative charges to outcompete stem RNAs accessing the helicase domain.

To investigate the second possibility that CHL destabilizes the RNA‐bound complex, we used the dissociation rates to estimate the lifetimes of stem RNA on the various constructs. Hel‐CTD forms a stable complex with the stem RNA with a lifetime of ~40 s. This lifetime was reduced to ~4 s when CHL was present (Figs 4E and EV4, Appendix Table S1B). Thus, CHL has a role in dissociating the stem RNA. However, cf190–210 Hel‐CTD does not appear to have a significantly different lifetime on stem RNA compared to CHL‐Hel‐CTD. While the CHL does stimulate RIG‐I dissociation from the RNA, it is not through the negative charge of region 190–210.

### The CHL and CARD2:Hel2i interface establish a tunable gating mechanism for RNA discrimination

Is CHL a general RNA competitor that competes with all RNAs or a selective competitor that blocks non‐specific RNAs that are not recruited by the CTD? To investigate the RNA proofreading function of the CHL, we added three new RNA ligands to our ligand panel with varying affinities for the RIG‐I CTD (5’ppp RNA, a PAMP; and 5’ ovg RNA and 3’ovg RNA, nonPAMPs). Previous studies have shown that 5’ppp RNA binds to CTD with the highest affinity (0.5 nM), followed by 3' ovg RNA (240 nM), 5' ovg RNA (840 nM), and stem RNA has no affinity to CTD (Ramanathan *et al*, 2016). This panel of RNAs with a range of affinity for the CTD enabled us to determine the role of CHL in regulating RNA binding into the helicase domain in context of CTD binding.

**Figure 5:**
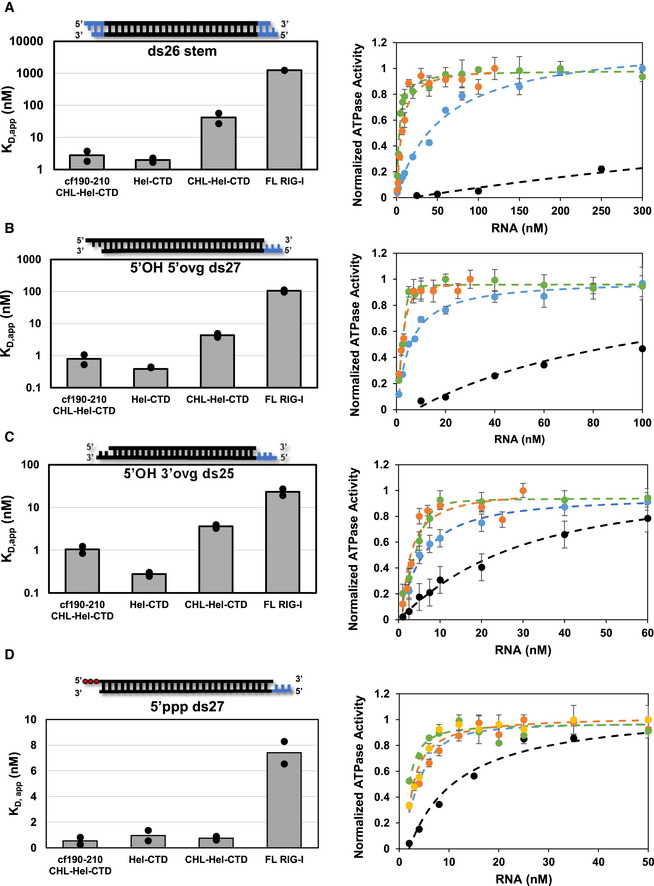
RNA proofreading contributions of CHL and CARD2:Hel2i interface A–DBar charts (left) and ATPase titrations (right) comparing the K_D,app_ values of (A) ds26 stem RNA, (B) 5’OH 5’ovg ds27, (C) 5’OH 3’ovg ds25, (D) 5’ppp ds27 for each of the RIG‐I constructs. Individual K_D,app_ values from two independent titrations are shown in the bar charts. Representative titration experiments show the ATPase activity plotted against RNA concentration for each of the four RIG‐I constructs. Each curve was normalized to the highest measured ATPase activity in each assay. WT RIG‐I (black filled circles), CHL‐Hel‐CTD (blue filled circles), Hel‐CTD (green filled circles), and CHL‐Hel‐CTD cf190–210 (orange filled circles). Standard error of fit from three reaction time points (mechanical replicate, *n* = 3) are shown for each point. Hel‐CTD and cf190–210 CHL‐Hel‐CTD titrations were fit using a quadratic equation (Equation 6), while all other RNAs tested were fit using a hyperbola (Equation 5) to obtain the K_D,app_ values (Fig EV5A). The K_D,app_ values are tabulated in Fig EV5A.

**Figure EV5:**
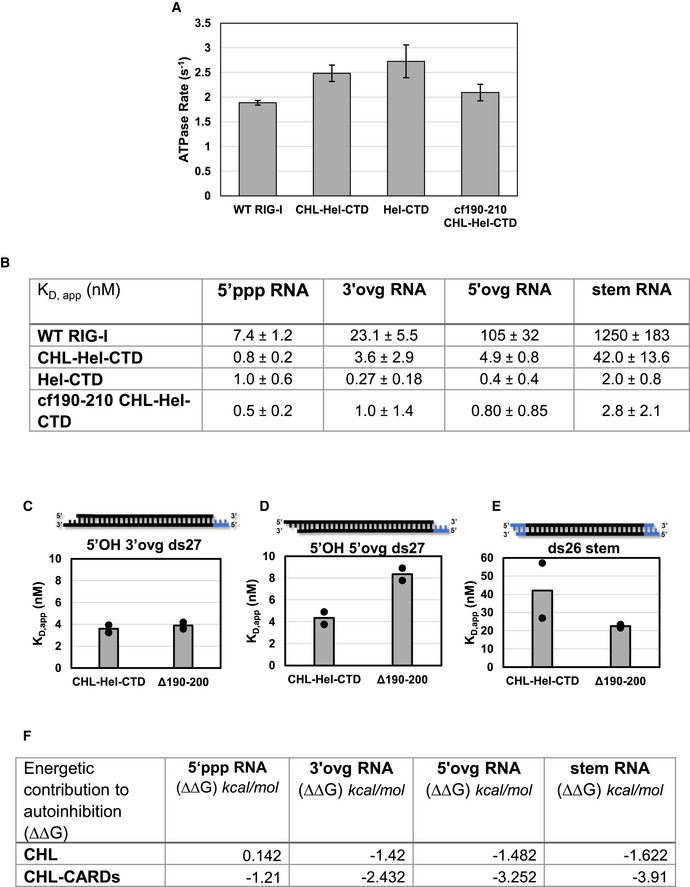
RNA binding studies of RIG‐I constructs ABar chart comparing each protein construct’s ATPase V_max_ tested simultaneously against either 5’ ppp ds27 from Fig 5 or No RNA. Note that no construct could hydrolyze ATP in the absence of bound RNA, typical of RIG‐I, and as such it is not shown. 10nM of protein was incubated with 100 nM of 5’ ppp ds27 RNA in all cases, and time course was performed with points taken at 0”, 30”, 60” and 90” (mechanical replicate, *n* = 3). Bars represent calculated V_max_ values. Error bars represent standard error of fit.BTable shows the RNA K_D,app_ values shown as bars in Fig 5. Standard errors are the average standard errors of fit for two binding trials, each trial with mechanical replicates of *n* = 3.C–EBar charts showing the K_D,app_ of CHL‐Hel‐CTD and ∆190–200 RIG‐I from titrations measuring ATPase as a function of RNA concentration. RNA schematic: Black: RNA, blue: DNA. Bar represents the mean K_D,app_ value of two independent RNA binding experiments, while dots indicate each independent RNA binding experiment. Each independent binding experiment had three time points (mechanical replicate, *n* = 3).FTable shows the energetic contributions of CHL and CHL‐CARDs to autoinhibiting RNA binding, calculated using K_D,app_ values in D and Equations 7, 8, and 9.

The RNA K_D,app_ values were estimated by incubating RIG‐I with different concentrations of RNA and measuring the RNA‐dependent ATPase activity. The resulting binding curves and K_D,app_ values in bar chart format are shown in Fig 5A–D for each of the RNAs and RIG‐I constructs and tabulated in Fig EV5A. Measuring RNA binding under ATP hydrolysis conditions mimics physiological conditions more so than previous fluorescence‐based experiments done in absence of ATP. To determine if the CHL contributes to RNA discrimination, we compared the RNA K_D,app_ values CHL‐Hel‐CTD to Hel‐CTD. For each of the non‐PAMP RNAs, we observed a weaker RNA affinity to CHL‐Hel‐CTD than to Hel‐CTD. However, all three non‐PAMP RNAs were inhibited by 11–15 fold, which suggests that CHL is a general RNA competitor of non‐PAMP RNAs. Interestingly, CHL‐Hel‐CTD does not show weaker binding to 5’ppp ds27 relative to Hel‐CTD, suggesting that CHL does not inhibit PAMP RNA binding. Furthermore, the data with cf190–210 mutant shows that CHL loses its ability to compete against non‐PAMP RNAs when the negative charges in the 190–210 region are mutated to lysines. Therefore, CHL uses its negative charges to specifically inhibit non‐PAMP RNA binding.

In the autoinhibited conformation of RIG‐I, the CARDs are sequestered by Hel2i through the CARD2:Hel2i interactions, which localizes CHL more closely near the helicase domain (Fig 1A). To determine the contribution of CARD2:Hel2i to RNA proofreading, we compared the RNA K_D,app_ values of WT RIG‐I/∆190–200 RIG‐I to Hel‐CTD. As shown above, ∆190–200 mutation disrupts the CARD2:Hel2i interface; hence, these experiments will provide the proofreading contribution of CHL linked to the autoinhibited CARDs versus the exposed CARDs. Depending on the RNA end modification, the CHL in WT RIG‐I weakens RNA binding by 5–700 fold. In contrast, CHL in ∆190–200 RIG‐I behaved like free CHL in CHL‐Hel‐CTD uniformly weakening RNA binding by 11–15 fold (Fig EV5B–D). These results demonstrate that CHL must be linked to the autoinhibited CARDs to discriminate between various RNA‐ends. Our data show that RNAs that have weaker affinities for the CTD are outcompeted by the CHL more effectively than those with higher affinities, like the 5’ppp RNA. Stem RNA with no affinity for the CTD was inhibited by 700‐fold, 5’ ovg RNA by 240‐fold, 3’ ovg RNA by 60‐fold, and while 5’ppp RNA binding was inhibited approximately 5‐fold.

**Figure 6:**
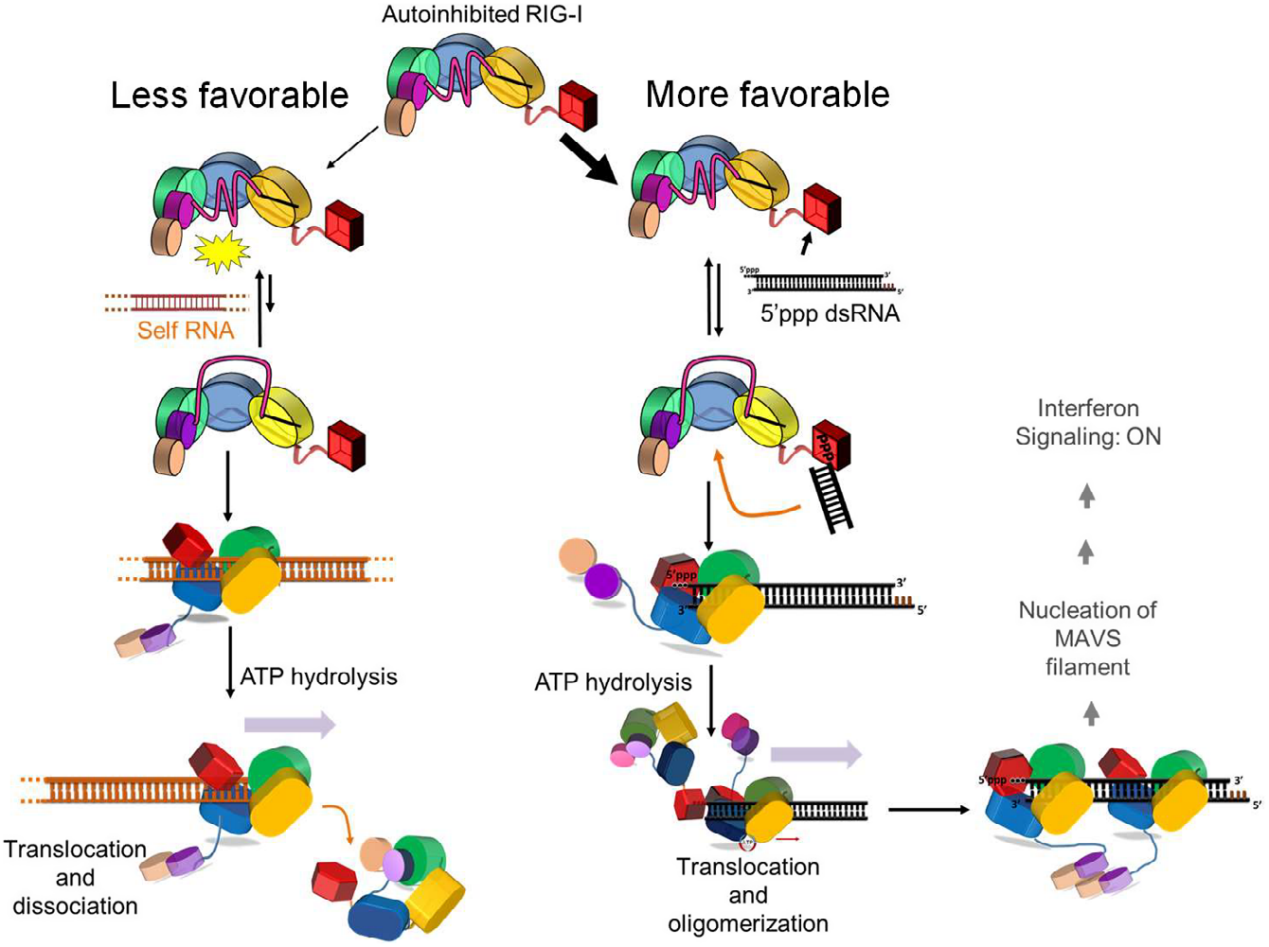
RNA proofreading mechanisms of RIG‐I The model shows how RIG‐I discriminates between self and non‐self RNAs using multiple layers of RNA proofreading. More favorable PAMP RNA binding (black) on the right contrasts with less favorable self‐RNA (orange, dashed lines indicate the RNA continues) binding on the left. In the resting state of RIG‐I, the Hel2i sequesters the CARDs in the autoinhibitory conformation and the CHL stabilizes this conformation while blocking RNA access into the helicase domain. In the autoinhibitory state, the CTD can freely access RNAs and detect PAMP RNA. The high selectivity of the CTD for PAMP RNA over self RNAs provides the first layer of RNA proofreading, while the CHL gate provides the next layer of RNA proofreading. The CHL gate blocks self RNA binding (in the left). However, PAMP RNA binds to CTD and can access the RNA binding site of the helicase domain when the CHL gate opens transiently. RNA binding induces conformational changes in the helicase domain exposing the CARDs. Due to favorable CTD interactions, CARDs exposure is more efficient on PAMP RNA than on self‐RNA. The ensuing ATP hydrolysis checks for erroneous RNA binding by promoting RIG‐I to translocate and dissociate from self‐RNAs. The CTD interactions throttle RIG‐I translocation on PAMP RNA and promote oligomerization, which facilitates CARDs:CARDs interactions and many downstream events to ultimately induce an IFN response.

From the RNA K_D,app_ values, we can calculate the energetic cost of the CHL gating mechanism. The net energy to displace CHL and bind non‐PAMP RNAs is 1.4 to 1.6 kcal/mol when it is not linked to the CARDs, and 2.4 to 4 kcal/mol, when CHL is linked to the autoinhibited CARDs (Fig EV5E). In the proposed model below (Fig 6), we explain how CHL and CARD2:Hel2i establish a tunable gating mechanism that proofreads RNAs, antagonizing non‐specific RNAs while allowing PAMP RNAs chosen by the CTD to bind into the helicase domain.

## Discussion

RIG‐I has evolved multiple mechanisms of proofreading to discriminate between self and non‐self RNAs. In this study, we have discovered a previously unknown RNA proofreading mechanism that RIG‐I uses to prevent the helicase domain from binding RNAs indiscriminately. This RNA proofreading mechanism involving the intrinsically disordered linker, CHL, is distinct from the ATPase‐mediated proofreading mechanism, characterized previously (Lassig *et al*, 2015; Devarkar *et al*, 2018). Our studies show that CHL has a dual role in regulating the helicase domain activities. It uses its negatively charged amino acids to stabilize the CARDs in the autoinhibitory conformation. CHL also shields the helicase domain and electrostatically competes with incoming RNAs to prevent erroneous binding to self RNAs. Both these functions of the CHL are essential in keeping RIG‐I in a silent signaling state in the absence of PAMP RNAs. The multiple layers of RNA proofreading, summarized in Fig 6, are necessary to ensure that cytoplasmic RNAs do not aberrantly activate the RIG‐I signaling pathway resulting in harmful autoinflammatory conditions.

RIG‐I’s CTD provides the first layer of proofreading by selectively binding to 5’ppp blunt‐end RNA ends, typically found in viral RNAs. Even in the autoinhibitory state of RIG‐I, CTD is free to survey RNA ends for PAMP features (Devarkar *et al*, 2018). During this process of RNA searching, the helicase domain must be shielded from non‐specific RNAs. We show that CHL electrostatically shields the positively charged helicase domain through its negative charges and prevents erroneous binding of non‐specific RNAs. This is consistent with the autoinhibited duck RIG‐I strucure that shows that the negatively charged CHL (190–240 aa) bridges the RNA binding channel between domains CARD2 and Hel1 (Kowalinski *et al*, 2011). The Alphafold predicts CHL to be in a random coil conformation and places it within the RNA binding channel of the helicase domain (Fig EV1A and B). Being intrinsically disordered, the CHL gate is expected to be dynamic and to exist in fully open and closed conformations, as shown in Fig 6. We also expect that the CARD2:Hel2i interactions keep the CHL gate mostly in the closed state. Under these conditions, the CHL gate can effectively block RNAs attempting to access the helicase’s RNA binding site from the solution, like non‐specific RNAs that have no affinity to the CTD. The likelihood of gate opening coinciding with RNA collision from the solution would be low. In contrast, CTD‐bound RNAs have an advantage as they remain in the vicinity of the helicase domain and can access the RNA binding site when the CHL gate transiently opens. In particular, RNAs with long lifetimes on the CTD, like the PAMP RNA, have a greater chance of binding into the helicase domain than those with shorter lifetimes, like the overhang RNAs. It is also possible that the CTD‐bound RNAs induce a conformational change to open the CHL gate. Once the RNA accesses the helicase domain, it faces another barrier; the RNA must have sufficient energy to break the CARD2:Hel2i interactions to fully engage with all three helicase subdomains. The energetic barrier of breaking the CARD2:Hel2i interface provides an additional layer of RNA proofreading. Those RNAs that have high affinities to CTD, like PAMP RNA, would have sufficient energy to overcome the energy barrier and activate the CARDs. Non‐PAMP RNAs, like the stem or overhang RNAs, that do not bind to CTD are less likely to activate the CARDs.

The next layer of RNA proofreading is provided by the ATPase activity, which is triggered when RNA completely engages the helicase domain. Several studies show that the ATPase activity of RIG‐I discriminates between PAMP and non‐PAMP RNAs (Patel *et al*, 2013; Anchisi *et al*, 2015; Louber *et al*, 2015; Devarkar *et al*, 2018). RIG‐I begins to translocate on RNA upon ATP hydrolysis (Myong *et al*, 2009; Devarkar *et al*, 2018). This translocation activity decreases the lifetime of RIG‐I on non‐PAMP RNAs that are not tethered to the CTD, while PAMP RNAs remain bound and promote RIG‐I clustering (Devarkar *et al*, 2018). The clustering of RIG‐I on PAMP RNA facilitates intermolecular CARDs:CARDs interactions necessary to activate the RIG‐I signaling pathway (Peisley *et al*, 2013). Thus, CTD plays a crucial role in PAMP RNA selection at all stages of RNA proofreading. The role of the CHL is to stabilize the autoinhibited state and ensure that RNAs chosen by the CTD are loaded into the helicase. The role of the ATPase activity is to check for erroneous binding and promote the formation of signaling competent complexes on PAMP RNAs.

Dysregulation at any of the stage in RNA proofreading can lead to RIG‐I activation in the absence of PAMP RNA. Published studies show the ATPase‐defective Singleton Merten syndrome (SMS) mutants are constitutively activated by self RNAs (Jang *et al*, 2015; Devarkar *et al*, 2018; Lassig *et al*, 2018). The SMS mutants are ATPase defective and hence unable to check for erroneous RNA binding. Moreover, being competent in ATP binding, the SMS mutants form long‐lived complexes on non‐PAMP RNAs and activate the CARDs (Devarkar *et al*, 2018; Zheng *et al*, 2018). The CHL mutants described here are constitutively activated for different reasons. The CHL mutations destabilize the autoinhibited CARDs and dysregulate the RNA gating mechanism which leads to erroneous RNA binding and CARDs activation. We show that the CHL mutants are not defective in ATPase activity and presumably able to proofread RNAs, but this proofreading component is not sufficient to dissociate the non‐PAMP RNAs. Thus far, there are no clinical reports of any autoimmune diseases associated with mutations in the CHL region. Perhaps a single amino acid change is not sufficient and multiple changes must be made to disrupt CHL’s functions. Interestingly, CHL is not unique to RIG‐I; our sequence analysis found a CHL‐like region in MDA5, but not in LGP2. The negatively charged CHL in MDA5 is twice the size of RIG‐I’s CHL. It will be interesting to investigate whether the CHL in MDA5 regulates RNA binding and CARDs activation, like the CHL does in RIG‐I.

Intrinsically disordered linkers are widely found in proteins and shown to have regulatory functions in many signaling proteins (Vuzman & Levy, 2012; van der Lee *et al*, 2014; Wright & Dyson, 2015). Flexible gates like the CHL are found in enzymes, acting as molecular gates that dynamically switch between open and closed states to control substrate access (Marques *et al*, 2017). These gates are recognized as hotspots for binding small molecules that disrupt the gating function and change the ligand binding properties by stabilizing the open or the closed state. The discovery of such a molecular gate in RIG‐I provides a unique opportunity to leverage this region in future RIG‐I‐based therapeutics. The CHL would be a more specific target, than the ubiquitous ATPase pocket, to modulate RIG‐I activity. For example, compounds that destabilize the CHL, akin to deletions and substitutions, may represent a new class of RIG‐I activating molecules that can stabilize the CARDs‐released state without directly targeting the CARD2:Hel2i interface.

## Materials and Methods

### Reagents and Tools Table

**Table:** 

Reagent/Resource	Source	Identifier
**Chemicals, peptides, and recombinant proteins**		
ATP, [γ‐32P]	Perkin Elmer	Cat. # NEG002A250 U
Dual‐Luciferase Reporter Assay	Promega	Cat. # E1980
In‐Fusion HD Cloning Kit	Takara	Cat. # 639648
**Experimental models: Cell lines**		
HEK293T Cell Line	Dr. Marcotrigiano	Devarkar *et al* (2016)
HEK293T RIG‐I KO Cell Line	Dr. Hornung and Dr. Sarkar	Zhu *et al* (2014)
**Antibodies**		
Phospho‐IRF‐3 (Ser396) (4D4G) Rabbit mAb	Cell Signaling Technology	4947S
IRF‐3 (D83B9) Rabbit mAb	Cell Signaling Technology	4302S
Myc Tag 9B11	Cell Signaling Technology	2276S
**Oligonucleotides**		
ss26 RNA chi (for stem dsRNA) 5′‐dAdTdAdCdGUCCUGAUAGUUAGUAUCdCdAdTdC‐3′	Dharmacon	Devarkar *et al* (2018)
complementary ss26 RNA chi intCy3 (for stem dsRNA) 5′‐dCdGdAdTdGGAUACUAAC(Cy3)UAUCAGGAdCdGdTdA‐3′	TriLink BioTechnologies	Devarkar *et al* (2018)
5’ OH ss29 RNA (for 5’ overhang) 5’‐CU AUA CGU CCU GAU AGU UAG UAU CCA UCG‐ 3’	Biosynthesis	This paper
5’OH ss25 RNA (for 3’overhang) 5’‐ACG UCC UGA UAG UUA GUA UCC AUC G‐3’	Trilink BioTechnologies	This paper
5’ppp ss27 RNA (for 5’ppp) 5’ ppp‐AUA CGU CCU GAU AGU UAG UAU CCA UCG‐3’	Trilink	Devarkar *et al* (2018)
ss30 complementary RNA chi 5’Biotin‐dGdCdT CGA UGG AUA CUA ACU AUC AGG ACG UAU‐Cy3‐3’	Dharmacon	This paper
ss27 complementary RNA 5’Biotin‐CGA UGG AUA CUA ACU AUC AGG ACG UAU‐DY547‐3’	Dharmancon	Devarkar *et al* (2018)
5’ppp ss39 (for 5’ppp ds39): 5’ ppp‐AGU UGG UGG UUG UUG UGU GUU UGU GGU UGG UUU GUU UGG‐3’	*In vitro* transcription	This paper
ss39 complementary RNA for 5’ppp ds39 with 3nt DNA overhang: 5'‐Desthiobiotin dT(dT‐FAM)dT CCA AAC AAA CCA ACC ACA AAC ACA CAA CAA CCA CCA ACU‐3’	Trilink	This paper
5’ ppp ss39 DNA transcription template strand 5’ CC AAA CAA ACC AAC CAC AAA CAC ACA ACA ACC ACC AAC TAA TAG TGA GTC GTA TTA CTG‐3’	IDT	This paper
5’ ppp ss39 DNA transcription nontemplate strand 5’ CAG TAA TAC GAC TCA CTA TTA GTT GGT GGT TGT TGT GTG TTT GTG GTT GGT TTG TTT GG‐3’	IDT	This paper
**qRT‐PCR** **primers**		
IFNB1 Forward: GGGACTGGACAATTGCTTCAA	IDT	This paper
Reverse: GCAGTACATTAGCCATCAGTCACTTAA		
MX1 Forward: CTGGGATTTTGGGGCTTT	IDT	This paper
Reverse: GGGATGTGGCTGGAGATG		
OAS1 Forward: GAAGGAAAGGTGCTTCCGAGGTAG	IDT	This paper
Reverse: AAGACAACCAGGTCAGCGTCAGAT		
ISG15 Forward: GAGAGGCAGCGAACTCATCT	IDT	This paper
Reverse: CTTCAGCTCTGACACCGACA		
GAPDH Forward: TCTCTGCCCCCTCTGCTG	IDT	This paper
Reverse: AGTCCTTCCACGATACCAAA		
**Recombinant DNA**		
pET28b 6xHis‐SUMO‐RIG‐I	Jiang *et al*, 2011	
pET28b 6xHis‐SUMO‐HelCTD	Jiang *et al*, 2011	
pET28b 6xHis‐SUMO‐Δ190–200‐RIG‐I	This paper	
pET28b 6xHis‐SUMO‐CHL‐Hel‐CTD	This paper	
pET28b 6xHis‐SUMO‐CHL‐Hel‐CTD‐cf190–210	This paper	
pcDNA 3.1 WT RIG‐I	Devarkar *et al*, 2016	
pcDNA 3.1 Hel‐CTD	This paper	
pcDNA 3.1 Δ190–200 RIG‐I	This paper	
pcDNA 3.1 Δ200–210 RIG‐I	This paper	
pcDNA 3.1 Δ210–220 RIG‐I	This paper	
pcDNA 3.1 Δ220–230 RIG‐I	This paper	
pcDNA 3.1 Δ230–240 RIG‐I	This paper	
pcDNA 3.1 cf190–200 RIG‐I	This paper	
pcDNA 3.1 cf200–210 RIG‐I	This paper	
pcDNA 3.1 cf210–220 RIG‐I	This paper	
pcDNA 3.1 neu190–200 RIG‐I	This paper	
pRL‐TK (Renilla luciferase plasmid)	Devarkar *et al* (2016)	
pLuc125 (Firefly luciferase plasmid)	Devarkar *et al* (2016)	
**Software and algorithms**		
Other		
TLC PEI Cellulose F	Millipore	Cat. # 105579

### Methods and Protocols

#### Cell lines

HEK293T cells (Sex: Female) were a gift from Prof. Charles Rice, Rockefeller University USA. This cell line was described and used previously (Ramanathan *et al*, 2016; Devarkar *et al*, 2018). HEK293T RIG‐I KO cells were kindly provided by Dr. Veit Hornung and Dr. Saumendra Sarkar (Zhu *et al*, 2014).

#### IFN‐β Reporter cell signaling assays

HEK293T and HEK293T RIG‐I KO cells were grown in at 5% CO_2_ and 37°C, in DMEM with 10% FBS in 6‐well plates to 60% confluence and cotransfected with firefly luciferase reporter plasmid (pLuc125 / 2.5 μg), Renilla luciferase reporter plasmid (pRL‐TK / 500 ng), and a plasmid carrying either the WT RIG‐I gene or mutant RIG‐I construct under the constitutively active CMV promoter (pcDNA 3.1 / 2 μg). The firefly luciferase gene is under the control of the interferon β promoter, and the Renilla luciferase plasmid is under the control of the constitutively active TK promoter. The plasmid transfections were carried out with X‐tremeGENE HP DNA Transfection Reagent (Roche). Cells were replated in 96‐well plates the next day at 2 × 10^4^ cells/well density and transfected with either 50 nM 5’ppp ds39 RNA or a titration of 700 nM, 70 nM, and 7 nM 5’ppp ds39 RNA using Lyovec transfection reagent (InvivoGen). After 20 h, the activities of firefly and Renilla luciferases were measured sequentially with the Dual‐Luciferase reporter assay (Promega). Data was collected in sextuplicate sets, and relative luciferase activities were calculated. Error bars represent the standard error of the mean (SEM). Protein expression was tested using western Blots with a primary α‐Myc antibody and β‐actin as a normalization control.

#### Quantitative real‐time PCR (qRT–PCR) analysis

Total RNA was extracted from transfected 293T cells using TRIzol reagent (Invitrogen, Carlsbad, CA) following the manufacturer’s instructions (Gokhale *et al*, 2020). RNA concentration and quality were assessed using a NanoDrop spectrophotometer. Complementary DNA (cDNA) was prepared from 500 ng of RNA using the High Capacity cDNA Reverse Transcription Kit (Applied Biosystems, Carlsbad, CA) (Lalani *et al*, 2015; Gokhale *et al*, 2020). Quantitative real‐time PCR of specific genes was performed using the Power SYBR Green PCR Master Mix (Applied Biosystems) and primers specific for human IFNB1, MX1, ISG15, OAS1, or GAPDH on a QuantStudio™ 3 Real‐Time PCR System (96‐well, 0.1 ml Block, Applied Biosystems). Post‐amplification melting curve analyses controlled the reaction specificity of each gene. Real‐time PCR data were analyzed using the QuantStudio™ Design and Analysis Software v1.5.2 (Applied Biosystems). Each gene's specific mRNA expression levels were normalized to the housekeeping gene GAPDH. For each target gene, the fold induction of each sample relative to the values of mock‐transfected cells was calculated using the comparative threshold cycle (Ct) method (∆∆Ct) as previously described (Edwards *et al*, 2014; Lalani *et al*, 2015).

#### Immunoblot analysis of IRF3 phosphorylation

Total protein lysates were prepared by lysing cell pellets in 2× SDS sample buffer (62.5 mM Tris, pH6.8, 1% SDS, 15% glycerol, 2% β‐mercaptoethanol and 0.005% bromophenol blue), sonicated for 30 pulses, and then boiled for 10 min (Edwards *et al*, 2014; Liu *et al*, 2021). Proteins were separated by SDS–PAGE and immunoblotted with antibodies to phosphorylated (P‐) or total IRF3, or to Myc tag, followed by HRP‐conjugated secondary antibodies (goat anti‐rabbit or goat anti‐mouse IgG) (Lalani *et al*, 2015; Liu *et al*, 2021). A chemiluminescent substrate (Pierce) was used to detect HRP‐labeled Abs on the immunoblots. Chemiluminescence images of the immunoblots were acquired using a low‐light imaging system (LAS‐4000 mini, FUJIFILM Medical Systems USA, Inc., Stamford, CT) (Moore *et al*, 2012; Edwards *et al*, 2013; Liu *et al*, 2021).

#### Protein expression and purification

RIG‐I constructs were cloned into the protein expression vector pET28 SUMO and expressed as SUMO fusion proteins in *Escherichia coli* strain Rosetta (DE3) (Novagen). The proteins were purified using a series of chromatography columns, as published previously (Jiang *et al*, 2011). The soluble lysate was fractionated through HisTrap HP (GE Healthcare), followed by Ulp1 protease digestion to remove 6xHis‐SUMO tag, hydroxyapatite (CHT‐II, Bio‐Rad), and heparin sepharose (GE Healthcare). The purified protein was dialyzed into 50 mM HEPES pH 7.5, 50 mM NaCl, 5 mM MgCl_2_, 5 mM DTT, and 10% glycerol overnight at 4°C, frozen in liquid nitrogen, and stored at −80°C.

#### RNA

The single‐stranded RNAs to make the ds27 PAMP and non‐PAMP RNA ligands, ds39 complementary RNA, and 5’ppp ds10 hairpin were chemically synthesized and HPLC purified by Biosynthesis, Dharmacon, and Trilink BioTechnologies. The 5’ppp 39‐mer RNA was *in vitro* transcribed using T7 RNA polymerase and gel purified. Synthetic RNAs were purity‐analyzed using mass spectrometry and HPLC. Lyophilized RNA was resuspended in 20 mM potassium phosphate buffer 7.0. Concentrations were determined from the A_260_, measured using a NanoDrop spectrophotometer, in 7 M guanidinium HCL, as well as the extinction coefficient for each RNA. RNAs were annealed by mixing complementary ssRNAs in a 1:1.1 ratio (fluorescent:nonfluorescent ratio, where applicable), then heating to 95°C, then finally cooling slowly to 4°C.

#### Hydrogen–Deuterium exchange (HDX) detected by mass spectrometry (MS)

Peptides were identified using tandem MS (MS/MS) with an Orbitrap mass spectrometer (Q Exactive, Thermo Fisher). Product ion spectra were acquired in data‐dependent mode with the top five most abundant ions selected for the product ion analysis per scan event. The MS/MS data files were submitted to Mascot (Matrix Science) for peptide identification. Peptides included in the HDX analysis peptide set had a MASCOT score greater than 20, and the MS/MS spectra were verified by manual inspection. The MASCOT search was repeated against a decoy (reverse) sequence and ambiguous identifications were ruled out and not included in the HDX peptide set.

For HDX‐MS analysis, 10 µM of WT/Δ190–200 RIG‐I receptors (50 mM HEPES, pH 7.4, 150 mM NaCl, 5% glycerol, 5 mM MgCl_2_, 2 mM DTT) were incubated with the RNA ligand at a 1:1.2 molar ratio for 1 hr (protein:ligand) before the HDX reactions at 4°C. Five‐microliter of protein/protein complex with ligand/peptide was diluted into 20 µl D_2_O (deuterium) in exchange buffer (50 mM HEPES, pH 7.4, 150 mM NaCl, 5 mM MgCl_2_, 2 mM DTT) and incubated for various HDX time points (e.g., 0, 30, 60, 300, 600, 900, 1,800 and 3,600 s) at 4°C and quenched with 25 µl of ice‐cold 4 M guanidine hydrochloride, 1% trifluoroacetic acid. Dmax samples were incubated in D_2_O in exchange buffer containing 3 M guanidine hydrochloride (50 mM HEPES, pH 7.4, 150 mM NaCl, 5 mM MgCl_2_, two mM DTT and 3 M guanidine hydrochloride) overnight at room temperature. The samples were immediately placed on dry ice after the quenching reactions until they were injected into the HDX platform. Upon injection, samples were passed through an immobilized pepsin column (2 mm × 2 cm) at 200 µl min^−1^, and the digested peptides were captured on a 2mm × 1cm C_8_ trap column (Agilent) and desalted. Peptides were separated across a 2.1mm × 5cm C_18_ column (1.9 μm Hypersil Gold, Thermo Fisher) with a linear gradient of 4% ‐ 40% CH_3_CN and 0.3% formic acid, over 5 min. Sample handling, protein digestion, and peptide separation were conducted at 4°C. Mass spectrometric data were acquired using an Orbitrap mass spectrometer (Q Exactive, Thermo Fisher) with a measured resolving power of 65,000 at *m*/*z* 400. HDX analyses were performed duplicate or triplicate, with single preparations of each protein‐ligand complex. The intensity weighted mean m/z centroid value of each peptide envelope was calculated and subsequently converted into a percentage of deuterium incorporation. In the absence of a fully deuterated control, corrections for back‐exchange were made by an estimated 70% deuterium recovery, and accounting for the known 80% deuterium content of the deuterium exchange buffer. When comparing the two samples, the perturbation %D is determined by calculating the difference between the two samples. HDX Workbench colors each peptide according to the smooth color gradient HDX perturbation key (D%) shown in each indicated figure. Differences in %D between −5% to 5% are considered non‐significant and are colored gray according to the HDX perturbation key. In addition, unpaired t‐tests were calculated to detect statistically significant (*P* < 0.05) differences between samples at each time point. At least one time point with a *P*‐value less than 0.05 was present for each peptide in the data set, confirming that the difference was significant (Patil *et al*, 2018; Zheng *et al*, 2018).

#### Fluorimetric titrations to measure RNA Binding

Fluorescence intensity measurements were carried out at 25°C using FluoroMax‐4 spectrofluorimeter (Horiba Jobin Yvon) in Buffer A (50 mM MOPS pH 7.4, 5 mM DTT, 5 mM MgCl_2_, 0.01% Tween20). Cy3‐labeled ds26 stem RNA or 5’ppp ds27 RNA (15 nM) was titrated with increasing concentration of RIG‐I proteins, and the change in fluorescence intensity was measured at 570 nm after excitation at 547 nm. The observed fluorescence intensity change (F) from the initial fluorescence intensity (F_0_) is proportional to the amount of protein‐RNA complex (PR) and modified by a coefficient of complex formation (f_c_). This value was plotted as a function of protein concentration (P) and fitted to Equation 1 to obtain the equilibrium dissociation constant (*K*_D_) and Hill coefficient (*n*). The reported RNA *K*_D_ values were consistently observed in titrations repeated two times.

(1) $$F=\;F_{0}+f_{c}\;*\;\left[PR\right]$$

(2) $$\left[PR\right]=\frac{{\left[P\right]}^{n}}{K_{D}^{n}+{\left[P\right]}^{n}}$$

#### Stopped‐flow experiments to measure RNA association and dissociation rate constants

The RNA dissociation rates were measured at 25°C using a stopped‐flow instrument (Auto‐SF 120, Kintek Corp, Austin, Tx). A mixture of Cy3‐labeled ds26 stem RNA mimic (100 nM) and protein (400 nM) in Buffer A (pre‐incubated at 25°C for 10 min) from syringe A was mixed with a 10‐fold excess of an RNA trap (unlabeled 5′ppp ds12 hairpin RNA) from syringe B. The Cy3 fluorescence emission was measured using a 570 nm band‐pass filter after excitation at 547 nm. The change in fluorescence intensity was plotted as a function of time, and the data were fit to a single exponential or sum of two to three exponentials to estimate the dissociation rates.

The RNA association rates were measured at 25°C using the stopped‐flow instrument. A fixed concentration (100 nM) of Cy3‐labeled ds26 stem RNA from syringe A was rapidly mixed with the RIG‐I construct (250 nM) from syringe B. The fluorescence intensity was plotted as a function of time and fitted a single exponential equation or sum of two to three exponentials to obtain the observed rates of binding.

Fitting association and dissociation kinetics yielded the rate constant (k) and the population fraction (A) associated with each phase. Average association and dissociation times (t) were calculated by taking the inverse of the rate constant and weighting it by population fraction, as shown below in Equation 3. Note that the sum of all population fractions for any given fitting will equal 1 (Equation 4 *)*.

(3) $$t=A_{1}\;*\;\frac{1}{k_{1}}+A_{2}\;*\;\frac{1}{k_{2}}+A_{3}\;*\;\frac{1}{k_{3}}$$

(4) $$A_{\text{total}}=A_{1}+A_{2}+A_{3}=1$$

#### RNA binding using ATP hydrolysis

ATP hydrolysis was measured at constant RIG‐I (15 nM) and increasing RNA concentration (1 nM – 5 μM) in the presence of 1 mM ATP (spiked with [γ‐^32^P] ATP). The ATPase reaction was measured after 20, 40, 60 min or 30, 60, 90 min of reaction times in Buffer A at 25°C. Reactions were stopped at each time point using 4 N formic acid and analyzed by PEI‐Cellulose‐F TLC (Merck) developed in 0.4 M potassium phosphate buffer (pH 3.4). TLC plates were exposed to a phosphorimager plate, imaged on a Typhoon phosphor‐imager, and quantified using the ImageQuant software. The molar [Pi] generated during the reaction time intervals was plotted against time and fit to a linear equation. The slopes (ATPase rate) were plotted as a function of RNA concentration and fitted to the following equation to obtain the RNA K_D,app_ values: slope = *k*_atpase_ × [PR]/[Pt]; where [Pt] is total protein concentration; [PR] is the amount of RIG‐I/RNA complex formed and [R] is the RNA concentration being titrated. The hyperbolic (Equation 5) or quadratic equation (Equation 6) was used to determine [PR] and estimate the K_D,app_ and *k*_atpase_. The energetic contributions of both the CHL and the combination of CHL‐CARDs are described in Equations 7, 8, and 9.

(5) $$\left[PR\right]=\frac{\left[P\right]}{K_{D}+\left[P\right]}$$

(6) $$\left[PR\right]=\frac{\left(\left[P_{t}\right]+\left[R_{t}\right]+K_{D,\;app}\right)‐\sqrt{{\left(\left[P_{t}\right]+\left[R_{t}\right]+K_{D,\;app}\right)}^{2}‐4{[P}_{t}]\left[R_{t}\right]}}{2}$$

(7) $${\Delta G}^{0}=RTln\left(K_{D,\;app}\right)$$

(8) $${\Delta \Delta G}_{\mathrm{CHL}}={\Delta G}_{Hel‐CTD}^{0}‐{\Delta G}_{CHL‐Hel‐CTD}^{0}$$

(9) $${\Delta \Delta G}_{CHL‐CARDs}={\Delta G}_{Hel‐CTD}^{0}‐{\Delta G}_{WT\;RIG‐I}^{0}$$

#### Electrophoretic Mobility Shift Assays (EMSA)

EMSAs were performed by incubating RIG‐I constructs (at 30 nM, 50 nM, or 70 nM), a 5’ ppp ds27 RNA carrying a 3’ DY547 fluorophore and distal 5’ biotin (50 nM), and monovalent streptavidin (55 nM) in Buffer A for 60 min at 4°C. 2 mM ATP was added to each reaction, where applicable, 15 min before loading the sample into the gel. Loading buffer (10× concentration of 1.5% Ficoll 400 in Tris‐borate buffer, pH 8.0) was added to the samples and run on a 4–16% gradient Native PAGE gel (Invitrogen) at 4°C. Gels were scanned at 532 nM using a Typhoon FLA 9500 laser‐based scanner (GE).

#### Quantification and statistical analysis

Luciferase signals in cell‐based signaling assays were measured using a luminometer (Tecan Spark) and quantified in Microsoft Excel. Steady‐state fluorescence intensity binding assays and ATP hydrolysis‐based binding assays were quantified and fitted using SigmaPlot v11.0 (Systat Software). Associated errors of the measurements and number of sample sets (*n*) are noted in the corresponding figure legends.

## Author contributions

**Brandon D Schweibenz:** Conceptualization; Data curation; Formal analysis; Investigation; Writing—original draft; Writing—review & editing. **Swapnil C Devarkar:** Conceptualization; Investigation; Writing—review & editing. **Mihai Solotchi:** Investigation. **Candice Craig:** Investigation. **Jie Zheng:** Investigation; Writing—review & editing. **Bruce D Pascal:** Investigation. **Patrick R Griffin:** Supervision; Writing—review & editing. **Samantha Gokhale:** Investigation. **Ping Xie:** Supervision; Investigation; Writing—review & editing. **Smita S Patel:** Conceptualization; Supervision; Funding acquisition; Writing—original draft; Project administration; Writing—review & editing.

In addition to the CRediT author contributions listed above, the contributions in detail are:

Conceptualization: BDS, SSP, SCD; Investigation: BDS, SCD, JZ, PX, SG, MS, CC, PRG, BDP; Writing—Original Draft: BDS, SSP; Writing—Review/Editing: All Authors; Funding Acquisition: SSP, PX.

## Disclosure and competing interests statement

The authors declare that they have no conflict of interest.

## Supporting information



Appendix

Expanded View Figures PDF

## Data Availability

Fluorescence intensity binding data, ATP hydrolysis binding data, cell signaling reporter data, and Western blots for main figures from this publication have been deposited to the BioStudies database (https://www.ebi.ac.uk/biostudies/) and assigned the identifier S-BSST801.

## References

[embj2021109782-bib-0001] Anchisi S , Guerra J , Garcin D (2015) RIG‐I ATPase activity and discrimination of self‐RNA versus non‐self‐RNA. MBio 6: e02349 25736886 10.1128/mBio.02349-14 PMC4358010

[embj2021109782-bib-0002] Civril F , Bennett M , Moldt M , Deimling T , Witte G , Schiesser S , Carell T , Hopfner KP (2011) The RIG‐I ATPase domain structure reveals insights into ATP‐dependent antiviral signalling. EMBO Rep 12: 1127–1134 21979817 10.1038/embor.2011.190 PMC3207106

[embj2021109782-bib-0003] Crowl JT , Gray EE , Pestal K , Volkman HE , Stetson DB (2017) Intracellular nucleic acid detection in autoimmunity. Annu Rev Immunol 35: 313–336 28142323 10.1146/annurev-immunol-051116-052331 PMC6435037

[embj2021109782-bib-0004] Cui S , Eisenacher K , Kirchhofer A , Brzozka K , Lammens A , Lammens K , Fujita T , Conzelmann KK , Krug A , Hopfner KP (2008) The C‐terminal regulatory domain is the RNA 5'‐triphosphate sensor of RIG‐I. Mol Cell 29: 169–179 18243112 10.1016/j.molcel.2007.10.032

[embj2021109782-bib-0005] Devarkar SC , Wang C , Miller MT , Ramanathan A , Jiang F , Khan AG , Patel SS , Marcotrigiano J (2016) Structural basis for m7G recognition and 2'‐O‐methyl discrimination in capped RNAs by the innate immune receptor RIG‐I. Proc Natl Acad Sci USA 113: 596–601 26733676 10.1073/pnas.1515152113 PMC4725518

[embj2021109782-bib-0006] Devarkar SC , Schweibenz B , Wang C , Marcotrigiano J , Patel SS (2018) RIG‐I uses an ATPase‐powered translocation‐throttling mechanism for kinetic proofreading of RNAs and oligomerization. Mol Cell 72: 355–368 30270105 10.1016/j.molcel.2018.08.021 PMC6434538

[embj2021109782-bib-0007] Dickey TH , Song B , Pyle AM (2019) RNA binding activates RIG‐I by releasing an autorepressed signaling domain. Sci Adv 5: eaax3641 31616790 10.1126/sciadv.aax3641 PMC6774723

[embj2021109782-bib-0008] Edwards SK , Moore CR , Liu Y , Grewal S , Covey LR , Xie P (2013) N‐benzyladriamycin‐14‐valerate (AD 198) exhibits potent anti‐tumor activity on TRAF3‐deficient mouse B lymphoma and human multiple myeloma. BMC Cancer 13: 481 24131623 10.1186/1471-2407-13-481 PMC3853153

[embj2021109782-bib-0009] Edwards SK , Baron J , Moore CR , Liu Y , Perlman DH , Hart RP , Xie P (2014) Mutated in colorectal cancer (MCC) is a novel oncogene in B lymphocytes. J Hematol Oncol 7: 56 25200342 10.1186/s13045-014-0056-6 PMC4172902

[embj2021109782-bib-0010] Gokhale S , Lu W , Zhu S , Liu Y , Hart RP , Rabinowitz JD , Xie P (2020) Elevated choline kinase alpha‐mediated choline metabolism supports the prolonged survival of TRAF3‐deficient B lymphocytes. J Immunol 204: 459–471 31826940 10.4049/jimmunol.1900658 PMC6946882

[embj2021109782-bib-0011] Goubau D , Schlee M , Deddouche S , Pruijssers AJ , Zillinger T , Goldeck M , Schuberth C , Van der Veen AG , Fujimura T , Rehwinkel J *et al* (2014) Antiviral immunity via RIG‐I‐mediated recognition of RNA bearing 5'‐diphosphates. Nature 514: 372–375 25119032 10.1038/nature13590 PMC4201573

[embj2021109782-bib-0012] Hu Y , Li W , Gao T , Cui Y , Jin Y , Li P , Ma Q , Liu X , Cao C (2017) The severe acute respiratory syndrome coronavirus nucleocapsid inhibits type I interferon production by interfering with TRIM25‐mediated RIG‐I ubiquitination. J Virol 91: e02143‐16 28148787 10.1128/JVI.02143-16 PMC5375661

[embj2021109782-bib-0013] Jang M‐A , Kim E , Now H , Nguyen N , Kim W‐J , Yoo J‐Y , Lee J , Jeong Y‐M , Kim C‐H , Kim O‐H *et al* (2015) Mutations in DDX58, which encodes RIG‐I, cause atypical Singleton‐Merten syndrome. Am J Hum Genet 96: 266–274 25620203 10.1016/j.ajhg.2014.11.019 PMC4320253

[embj2021109782-bib-0014] Jiang F , Ramanathan A , Miller MT , Tang GQ , Gale Jr M , Patel SS , Marcotrigiano J (2011) Structural basis of RNA recognition and activation by innate immune receptor RIG‐I. Nature 479: 423–427 21947008 10.1038/nature10537 PMC3430514

[embj2021109782-bib-0015] Jumper J , Evans R , Pritzel A , Green T , Figurnov M , Ronneberger O , Tunyasuvunakool K , Bates R , Žídek A , Potapenko A *et al* (2021) Highly accurate protein structure prediction with AlphaFold. Nature 596: 583–589 34265844 10.1038/s41586-021-03819-2 PMC8371605

[embj2021109782-bib-0016] Kato H , Takeuchi O , Sato S , Yoneyama M , Yamamoto M , Matsui K , Uematsu S , Jung A , Kawai T , Ishii KJ *et al* (2006) Differential roles of MDA5 and RIG‐I helicases in the recognition of RNA viruses. Nature 441: 101–105 16625202 10.1038/nature04734

[embj2021109782-bib-0017] Kowalinski E , Lunardi T , McCarthy AA , Louber J , Brunel J , Grigorov B , Gerlier D , Cusack S (2011) Structural basis for the activation of innate immune pattern‐recognition receptor RIG‐I by viral RNA. Cell 147: 423–435 22000019 10.1016/j.cell.2011.09.039

[embj2021109782-bib-0018] Lalani AI , Moore CR , Luo C , Kreider BZ , Liu Y , Morse 3rd HC , Xie P (2015) Myeloid cell TRAF3 regulates immune responses and inhibits inflammation and tumor development in mice. J Immunol 194: 334–348 25422508 10.4049/jimmunol.1401548 PMC4272913

[embj2021109782-bib-0019] Lassig C , Matheisl S , Sparrer KM , de Oliveira Mann CC , Moldt M , Patel JR , Goldeck M , Hartmann G , Garcia‐Sastre A , Hornung V *et al* (2015) ATP hydrolysis by the viral RNA sensor RIG‐I prevents unintentional recognition of self‐RNA. eLife 4: e10859 26609812 10.7554/eLife.10859 PMC4733034

[embj2021109782-bib-0020] Lassig C , Lammens K , Gorenflos Lopez JL , Michalski S , Fettscher O , Hopfner KP (2018) Unified mechanisms for self‐RNA recognition by RIG‐I Singleton‐Merten syndrome variants. eLife 7: e38958 30047865 10.7554/eLife.38958 PMC6086658

[embj2021109782-bib-0021] Liu Y , Gokhale S , Jung J , Zhu S , Luo C , Saha D , Guo JY , Zhang H , Kyin S , Zong W‐X *et al* (2021) Mitochondrial fission factor is a novel interacting protein of the critical B cell survival regulator TRAF3 in B lymphocytes. Front Immunol 12: 670338 34745083 10.3389/fimmu.2021.670338 PMC8564014

[embj2021109782-bib-0022] Louber J , Brunel J , Uchikawa E , Cusack S , Gerlier D (2015) Kinetic discrimination of self/non‐self RNA by the ATPase activity of RIG‐I and MDA5. BMC Biol 13: 54 26215161 10.1186/s12915-015-0166-9 PMC4517655

[embj2021109782-bib-0023] Lu C , Xu H , Ranjith‐Kumar CT , Brooks MT , Hou TY , Hu F , Herr AB , Strong RK , Kao CC , Li P (2010) The structural basis of 5' triphosphate double‐stranded RNA recognition by RIG‐I C‐terminal domain. Structure 18: 1032–1043 20637642 10.1016/j.str.2010.05.007 PMC2919622

[embj2021109782-bib-0024] Luo D , Ding SC , Vela A , Kohlway A , Lindenbach BD , Pyle AM (2011) Structural insights into RNA recognition by RIG‐I. Cell 147: 409–422 22000018 10.1016/j.cell.2011.09.023 PMC3222294

[embj2021109782-bib-0025] Luo D , Kohlway A , Vela A , Pyle AM (2012) Visualizing the determinants of viral RNA recognition by innate immune sensor RIG‐I. Structure 20: 1983–1988 23022350 10.1016/j.str.2012.08.029 PMC3515076

[embj2021109782-bib-0026] Marques SM , Daniel L , Buryska T , Prokop Z , Brezovsky J , Damborsky J (2017) Enzyme tunnels and gates as relevant targets in drug design. Med Res Rev 37: 1095–1139 27957758 10.1002/med.21430

[embj2021109782-bib-0027] Moore CR , Liu Y , Shao C , Covey LR , Morse 3rd HC , Xie P (2012) Specific deletion of TRAF3 in B lymphocytes leads to B‐lymphoma development in mice. Leukemia 26: 1122–1127 22033491 10.1038/leu.2011.309 PMC3433763

[embj2021109782-bib-0028] Myong S , Cui S , Cornish PV , Kirchhofer A , Gack MU , Jung JU , Hopfner KP , Ha T (2009) Cytosolic viral sensor RIG‐I is a 5'‐triphosphate‐dependent translocase on double‐stranded RNA. Science 323: 1070–1074 19119185 10.1126/science.1168352 PMC3567915

[embj2021109782-bib-0029] Oates ME , Romero P , Ishida T , Ghalwash M , Mizianty MJ , Xue B , Dosztanyi Z , Uversky VN , Obradovic Z , Kurgan L *et al* (2013) D(2)P(2): database of disordered protein predictions. Nucleic Acids Res 41: D508–D516 23203878 10.1093/nar/gks1226 PMC3531159

[embj2021109782-bib-0030] Patel JR , Jain A , Chou YY , Baum A , Ha T , Garcia‐Sastre A (2013) ATPase‐driven oligomerization of RIG‐I on RNA allows optimal activation of type‐I interferon. EMBO Rep 14: 780–787 23846310 10.1038/embor.2013.102 PMC3790048

[embj2021109782-bib-0031] Patil DN , Rangarajan ES , Novick SJ , Pascal BD , Kojetin DJ , Griffin PR , Izard T , Martemyanov KA (2018) Structural organization of a major neuronal G protein regulator, the RGS7‐Gbeta5‐R7BP complex. eLife 7: e42150 30540250 10.7554/eLife.42150 PMC6310461

[embj2021109782-bib-0032] Peisley A , Wu B , Yao H , Walz T , Hur S (2013) RIG‐I forms signaling‐competent filaments in an ATP‐dependent, ubiquitin‐independent manner. Mol Cell 51: 573–583 23993742 10.1016/j.molcel.2013.07.024

[embj2021109782-bib-0033] Peisley A , Wu B , Xu H , Chen ZJ , Hur S (2014) Structural basis for ubiquitin‐mediated antiviral signal activation by RIG‐I. Nature 509: 110–114 24590070 10.1038/nature13140 PMC6136653

[embj2021109782-bib-0034] Poeck H , Bscheider M , Gross O , Finger K , Roth S , Rebsamen M , Hannesschlager N , Schlee M , Rothenfusser S , Barchet W *et al* (2010) Recognition of RNA virus by RIG‐I results in activation of CARD9 and inflammasome signaling for interleukin 1 beta production. Nat Immunol 11: 63–1824 19915568 10.1038/ni.1824

[embj2021109782-bib-0035] Ramanathan A , Devarkar SC , Jiang F , Miller MT , Khan AG , Marcotrigiano J , Patel SS (2016) The autoinhibitory CARD2‐Hel2i Interface of RIG‐I governs RNA selection. Nucleic Acids Res 44: 896–909 26612866 10.1093/nar/gkv1299 PMC4737149

[embj2021109782-bib-0036] Rehwinkel J , Tan CP , Goubau D , Schulz O , Pichlmair A , Bier K , Robb N , Vreede F , Barclay W , Fodor E *et al* (2010) RIG‐I detects viral genomic RNA during negative‐strand RNA virus infection. Cell 140: 397–408 20144762 10.1016/j.cell.2010.01.020

[embj2021109782-bib-0037] Roers A , Hiller B , Hornung V (2016) Recognition of endogenous nucleic acids by the innate immune system. Immunity 44: 739–754 27096317 10.1016/j.immuni.2016.04.002

[embj2021109782-bib-0038] Schuberth‐Wagner C , Ludwig J , Bruder A , Herzner A‐M , Zillinger T , Goldeck M , Schmidt T , Schmid‐Burgk J , Kerber R , Wolter S *et al* (2015) A conserved histidine in the RNA sensor RIG‐I controls immune tolerance to N1–2'O‐methylated self RNA. Immunity 43: 41–51 26187414 10.1016/j.immuni.2015.06.015 PMC7128463

[embj2021109782-bib-0039] Stumper R , Loo YM , Foy E , Li K , Yoneyama M , Fujita T , Lemon SM , Gale M (2005) Regulating intracellular antiviral defense and permissiveness to hepatitis C virus RNA replication through a cellular RNA helicase, RIG‐I. J Virol 79: 2689–2699 15708988 10.1128/JVI.79.5.2689-2699.2005 PMC548482

[embj2021109782-bib-0040] van der Lee R , Buljan M , Lang B , Weatheritt RJ , Daughdrill GW , Dunker AK , Fuxreiter M , Gough J , Gsponer J , Jones DT *et al* (2014) Classification of intrinsically disordered regions and proteins. Chem Rev 114: 6589–6631 24773235 10.1021/cr400525m PMC4095912

[embj2021109782-bib-0041] Vela A , Fedorova O , Ding SC , Pyle AM (2012) The thermodynamic basis for viral RNA detection by the RIG‐I innate immune sensor. J Biol Chem 287: 42564–42573 23055530 10.1074/jbc.M112.385146 PMC3522258

[embj2021109782-bib-0042] Vuzman D , Levy Y (2012) Intrinsically disordered regions as affinity tuners in protein‐DNA interactions. Mol Biosyst 8: 47–57 21918774 10.1039/c1mb05273j

[embj2021109782-bib-0043] Wang Y , Ludwig J , Schuberth C , Goldeck M , Schlee M , Li H , Juranek S , Sheng G , Micura R , Tuschl T *et al* (2010) Structural and functional insights into 5'‐ppp RNA pattern recognition by the innate immune receptor RIG‐I. Nat Struct Mol Biol 17: 781–787 20581823 10.1038/nsmb.1863 PMC3744876

[embj2021109782-bib-0044] Wright PE , Dyson HJ (2015) Intrinsically disordered proteins in cellular signalling and regulation. Nat Rev Mol Cell Biol 16: 18–29 25531225 10.1038/nrm3920 PMC4405151

[embj2021109782-bib-0045] Wu B , Peisley A , Tetrault D , Li Z , Egelman EH , Magor KE , Walz T , Penczek PA , Hur S (2014) Molecular imprinting as a signal‐activation mechanism of the viral RNA sensor RIG‐I. Mol Cell 55: 511–523 25018021 10.1016/j.molcel.2014.06.010 PMC4142144

[embj2021109782-bib-0046] Yoneyama M , Kikuchi M , Natsukawa T , Shinobu N , Imaizumi T , Miyagishi M , Taira K , Akira S , Fujita T (2004) The RNA helicase RIG‐I has an essential function in double‐stranded RNA‐induced innate antiviral responses. Nat Immunol 5: 730–737 15208624 10.1038/ni1087

[embj2021109782-bib-0047] Yoneyama M , Kikuchi M , Matsumoto K , Imaizumi T , Miyagishi M , Taira K , Foy E , Loo Y‐M , Gale M , Akira S *et al* (2005) Shared and unique functions of the DExD/H‐box helicases RIG‐I, MDA5, and LGP2 in antiviral innate immunity. J Immunol 175: 2851–2858 16116171 10.4049/jimmunol.175.5.2851

[embj2021109782-bib-0048] Zheng J , Yong HY , Panutdaporn N , Liu C , Tang K , Luo D (2015) High‐resolution HDX‐MS reveals distinct mechanisms of RNA recognition and activation by RIG‐I and MDA5. Nucleic Acids Res 43: 1216–1230 25539915 10.1093/nar/gku1329 PMC4333383

[embj2021109782-bib-0049] Zheng J , Wang C , Chang MR , Devarkar SC , Schweibenz B , Crynen GC , Garcia‐Ordonez RD , Pascal BD , Novick SJ , Patel SS *et al* (2018) HDX‐MS reveals dysregulated checkpoints that compromise discrimination against self RNA during RIG‐I mediated autoimmunity. Nat Commun 9: 5366 30560918 10.1038/s41467-018-07780-z PMC6299088

[embj2021109782-bib-0050] Zheng J , Strutzenberg T , Pascal BD , Griffin PR (2019) Protein dynamics and conformational changes explored by hydrogen/deuterium exchange mass spectrometry. Curr Opin Struct Biol 58: 305–313 31351767 10.1016/j.sbi.2019.06.007

[embj2021109782-bib-0051] Zhu J , Zhang Y , Ghosh A , Cuevas R , Forero A , Dhar J , Ibsen M , Schmid‐Burgk J , Schmidt T , Ganapathiraju M *et al* (2014) Antiviral activity of human OASL protein is mediated by enhancing signaling of the RIG‐I RNA sensor. Immunity 40: 936–948 24931123 10.1016/j.immuni.2014.05.007 PMC4101812

