# Supplementary material for: The intrinsically disordered CARDs‐Helicase linker in RIG‐I is a molecular gate for RNA proofreading
Source: EMBO J. 2022 Apr 19;41(10):e109782. doi: 10.15252/embj.2021109782 (PMC9108607; doi:10.15252/embj.2021109782)
Supplement: Supplementary file 1 — Appendix [file EMBJ-41-e109782-s002.pdf]

# Table of Contents

**Figure S1**: Hydrogen-Deuterium Exchange Mass Spectroscopy (HDX) heatmap comparing WT RIG-I with and without RNA binding…………………………………**Page 2**

**Figure S2:** Hydrogen-Deuterium Exchange Mass Spectroscopy (HDX) heatmap comparing Δ190-200 RIG-I with and without RNA binding…………………………..**Page 3**

**Figure S3:** Purified RIG-I proteins and their binding and oligomerization on 5'ppp RNA with and without ATP………………………………………………………………….**Page 4**

**Table S1:** ds26 stem RNA association and dissociation kinetic constants………..**Page 5**

WT RIG-I +/- 5’ppp HP

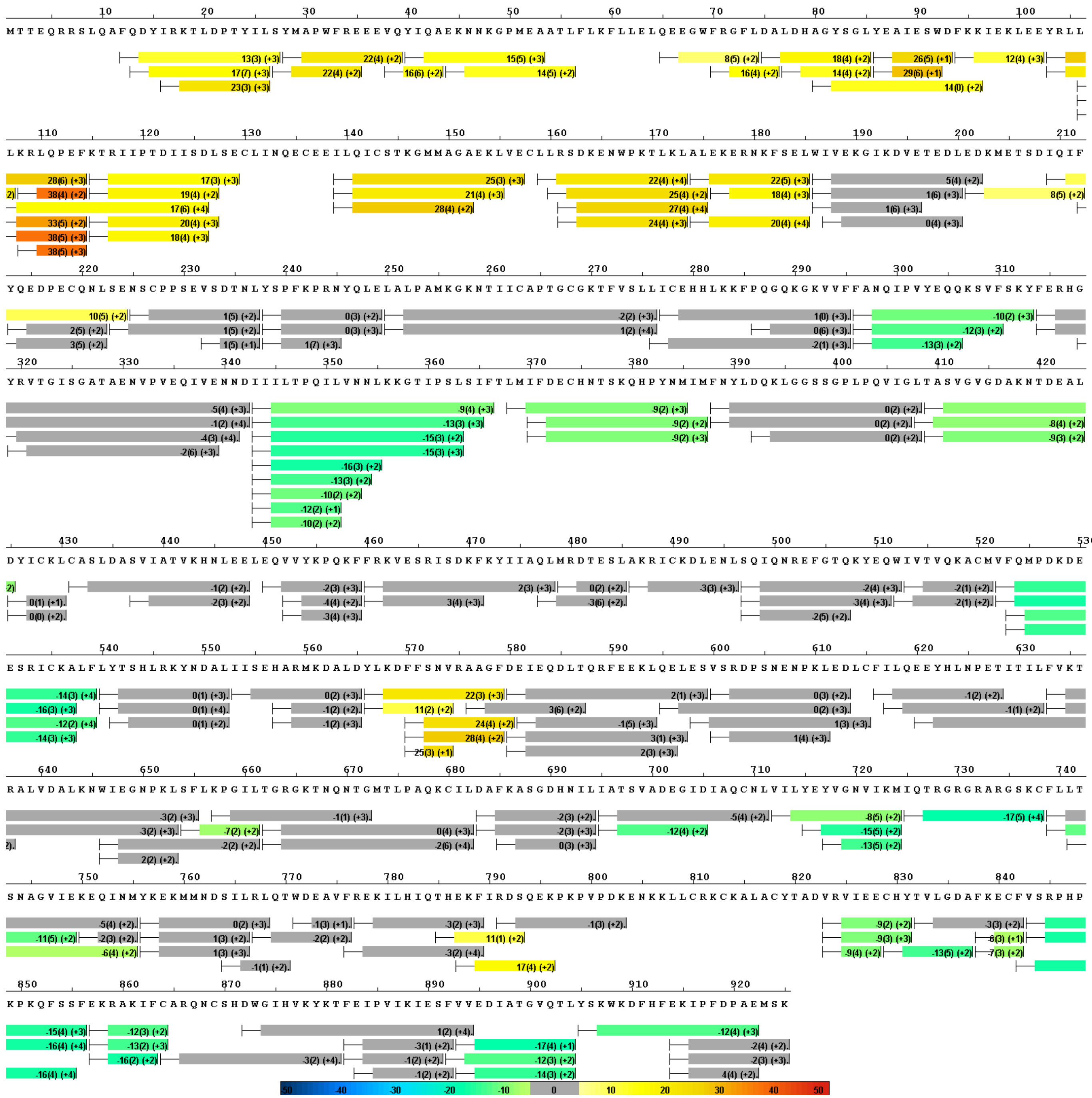

**Appendix Figure S1. Hydrogen-Deuterium Exchange Mass Spectroscopy (HDX) heatmap comparing WT RIG-I with and without RNA binding.** Panels shows the average ΔD2O% ± standard deviation between WT RIG-I with and without bound RNA across all HDX time points. HDX Workbench colors each peptide according to the smooth color gradient HDX perturbation key shown in each indicated figure. Average ΔD2O% between -5% to 5% are considered non-significant and are colored gray.

Δ190-200 RIG-I +/1 5'ppp HP

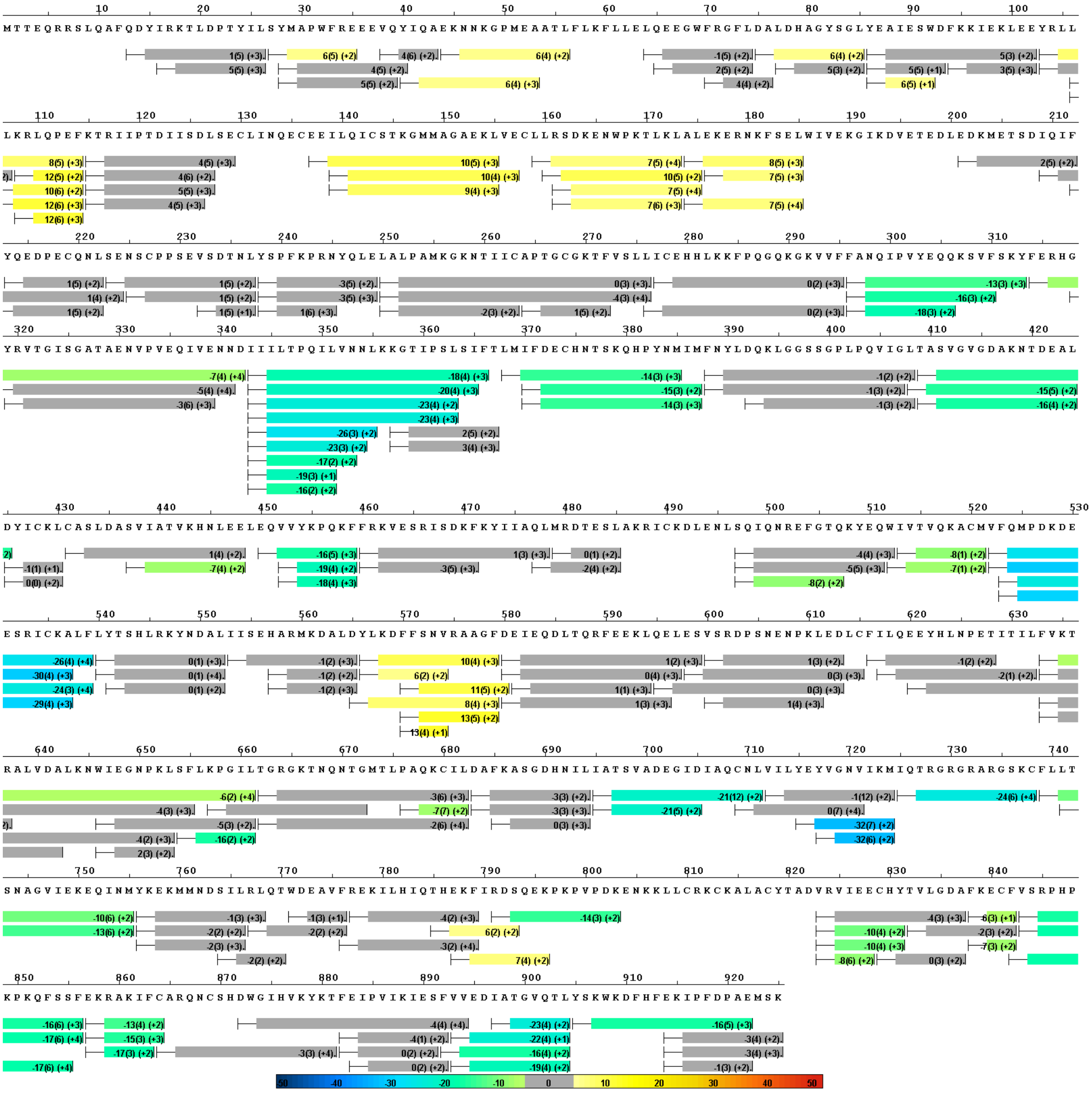

**Appendix Figure S2. Hydrogen-Deuterium Exchange Mass Spectroscopy (HDX) heatmap comparing Δ190-200 RIG-I with and without RNA binding**. Panels shows the average $\Delta D_2O\%$ ± standard deviation between Δ190-200 RIG-I with and without bound RNA across all HDX time points. HDX Workbench colors each peptide according to the smooth color gradient HDX perturbation key shown in each indicated figure. Average $\Delta D_2O\%$ between -5% to 5% are considered non-significant and are colored gray.

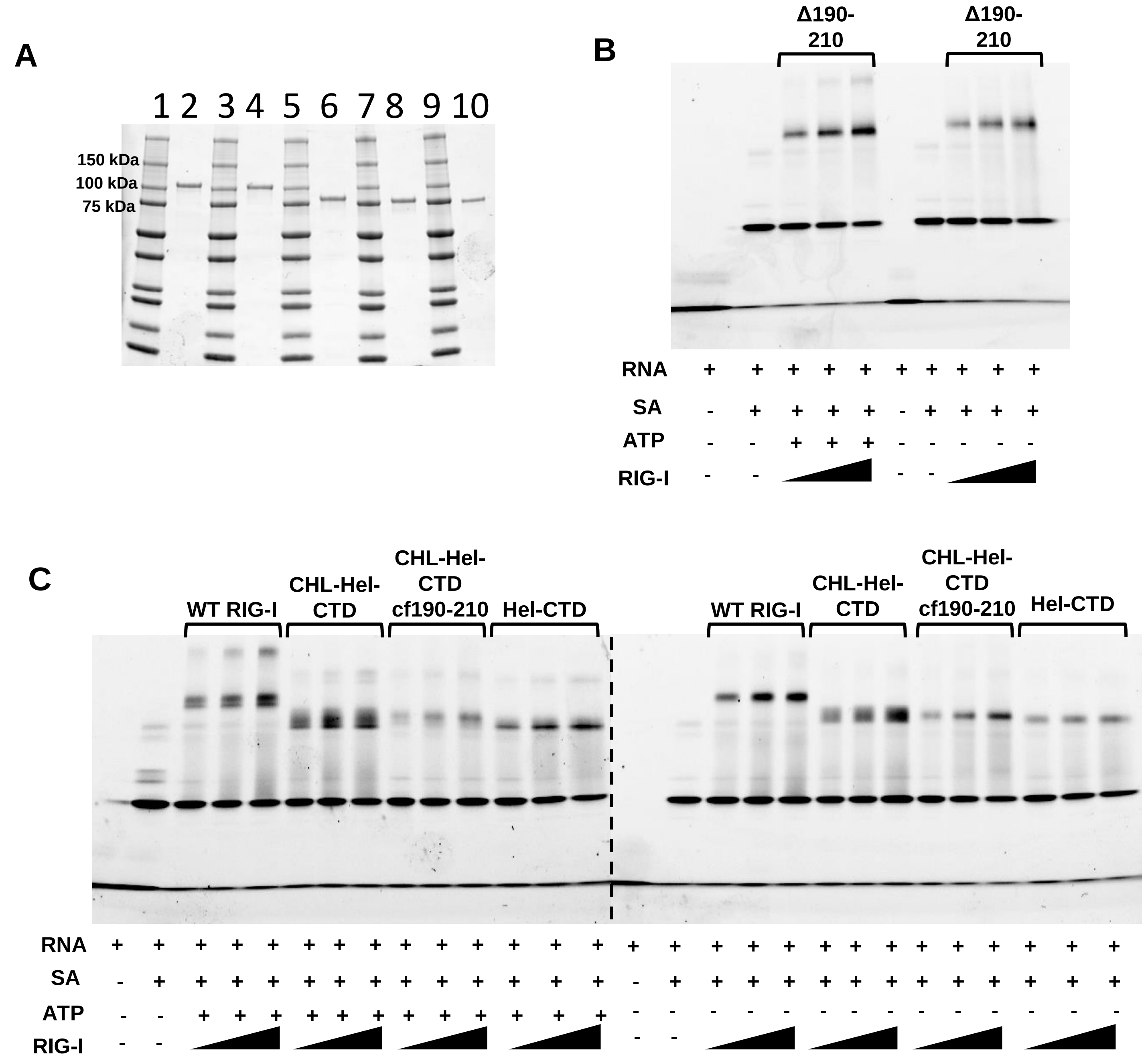


**Appendix Figure S3. Purified RIG-I proteins and their binding and oligomerization on 5'ppp RNA with and without ATP**

(A) SDS PAGE shows the various RIG-I protein constructs. Odd lanes are protein markers. Lane 2: Full length WT RIG-I (107 kDa), lane 4: Δ190-200 RIG-I (105 kDa), lane 6: CHL-Hel-CTD (85 kDa), lane 8: CHL-Hel-CTD cf190-210 (85 kDa), lane 10: Hel-CTD (79 kDa). (B) – (C) EMSA shows binding and oligomerization of various RIG-I constructs on 5'ppp ds27 RNA used in Figure 4 (with streptavidin to block binding from the non-ppp end) in the presence and absence of ATP. RIG-I was incubated at either 30 nM, 50 nM, or 70 nM with 50 nM of 5'ppp ds27 3'DY547 with distal 5' biotin and 55 nM of monovalent streptavidin (SA) for 60 minutes at 4oC. 2 mM ATP was added, where indicated, 15 minutes before loading reactions into the gel.

**A. Association coefficients**

| **RIG-I Construct** | $A_1$ | $k_1$ ($s^{-1}$) | $A_2$ | $k_2$ ($s^{-1}$) | $A_3$ | $k_3$ ($s^{-1}$) |
|---|---|---|---|---|---|---|
| WT RIG-I | 1 | 0.21 ± 0.16 | - | - | - | - |
| CHL-Hel-CTD | 0.33 | 1.99 ± 0.78 | 0.67 | 0.26 ± 0.05 | - | - |
| Δ190-200 | 0.35 | 1.75 ± 0.41 | 0.65 | 0.21 ± 0.03 | - | - |
| Hel-CTD | 0.70 | 260 ± 8 | 0.30 | 9.4 ± 0.5 | - | - |
| Hel-CTD cf190-210 | 0.59 | 560 ± 40 | 0.27 | 110 ± 14 | 0.14 | 7.4 ± 0.7 |

**B. Dissociation coefficients**

| **RIG-I Construct** | $A_1$ | $k_1$ ($s^{-1}$) | $A_2$ | $k_2$ ($s^{-1}$) | $A_3$ | $k_3$ ($s^{-1}$) |
|---|---|---|---|---|---|---|
| WT RIG-I | 1 | 0.25 ± 0.09 | - | - | - | - |
| CHL-Hel-CTD | 1 | 0.33 ± 0.04 | - | - | - | - |
| Δ190-200 | 0.13 | 2.6 ± 1.0 | 0.87 | 0.22 ± 0.02 | - | - |
| Hel-CTD | 0.09 | 0.84 ± 0.13 | 0.43 | 0.070 ± 0.005 | 0.48 | 0.013 ± 0.001 |
| Hel-CTD cf190-210 | 0.10 | 33 ± 4 | 0.50 | 0.67 ± 0.04 | 0.40 | 0.12 ± 0.008 |

**Appendix Table S1. ds26 stem RNA association and dissociation kinetic constants.** The stopped-flow kinetics of ds26 stem RNA association (A) and dissociation (B) experiments shown in Figure EV4 were fit to one or the sum of two or three exponentials and the associated coefficients are shown. An refers to the population fraction and kn the rate constant. Each fit is an average of at least 6 individual traces. Standard errors of the fit are shown.
