# Supplementary material for: The intrinsically disordered CARDs‐Helicase linker in RIG‐I is a molecular gate for RNA proofreading
Source: EMBO J. 2022 Apr 19;41(10):e109782. doi: 10.15252/embj.2021109782 (PMC9108607; doi:10.15252/embj.2021109782)
Supplement: Supplementary file 2 — Expanded View Figures PDF [file EMBJ-41-e109782-s001.pdf]

Expanded View Figures

Figure EV1. Induction of the IFN response genes by the CHL mutants.

A AlphaFold predicted structure of RIG-I shows CHL bound in the RNA binding pocket of the helicase domain. The colors correspond to model per-residue confidence (pLDDT): Dark blue, very high (pLDDT > 90), light blue, confident (90 > pLDDT > 70), yellow, low (70 > pLDDT > 50), orange, very low (pLDDT < 50). Regions below 50 pLDDT are predicted to be unstructured, like the CHL in orange.

B Graph demonstrating per-residue confidence (pLDDT) of AlphaFold prediction shown in (A), indicating low-confidence in the prediction of CHL structure. The CHL (186–241) is highlighted in orange.

C Western Blot confirms RIG-I expression in the reporter assays in Fig 1. In each experiment, pcDNA3.1 myc-tagged RIG-I constructs (approximately 108 kDa) were recognized with a primary α-Myc antibody. β-actin (approximately 42 kDa) was used as a normalization control. Numbers (left) refer to molecular weight in kDa. Note that HEK293T and HEK293T RIG-I KO Western blots were performed on separate gels.

D–G qRT–PCR assays show the induction of antiviral IFN response genes in the absence and presence of PAMP RNA, 5'ppp ds39. Note the Y-axis is in log scale. Each bar represents the mean ± SD. Each point represents a mechanical replicate ($n = 2$).

H Western blot to show induction of pIRF3 in RIG-I and RIG-I mutant transfected cells.

© 2022 The Authors

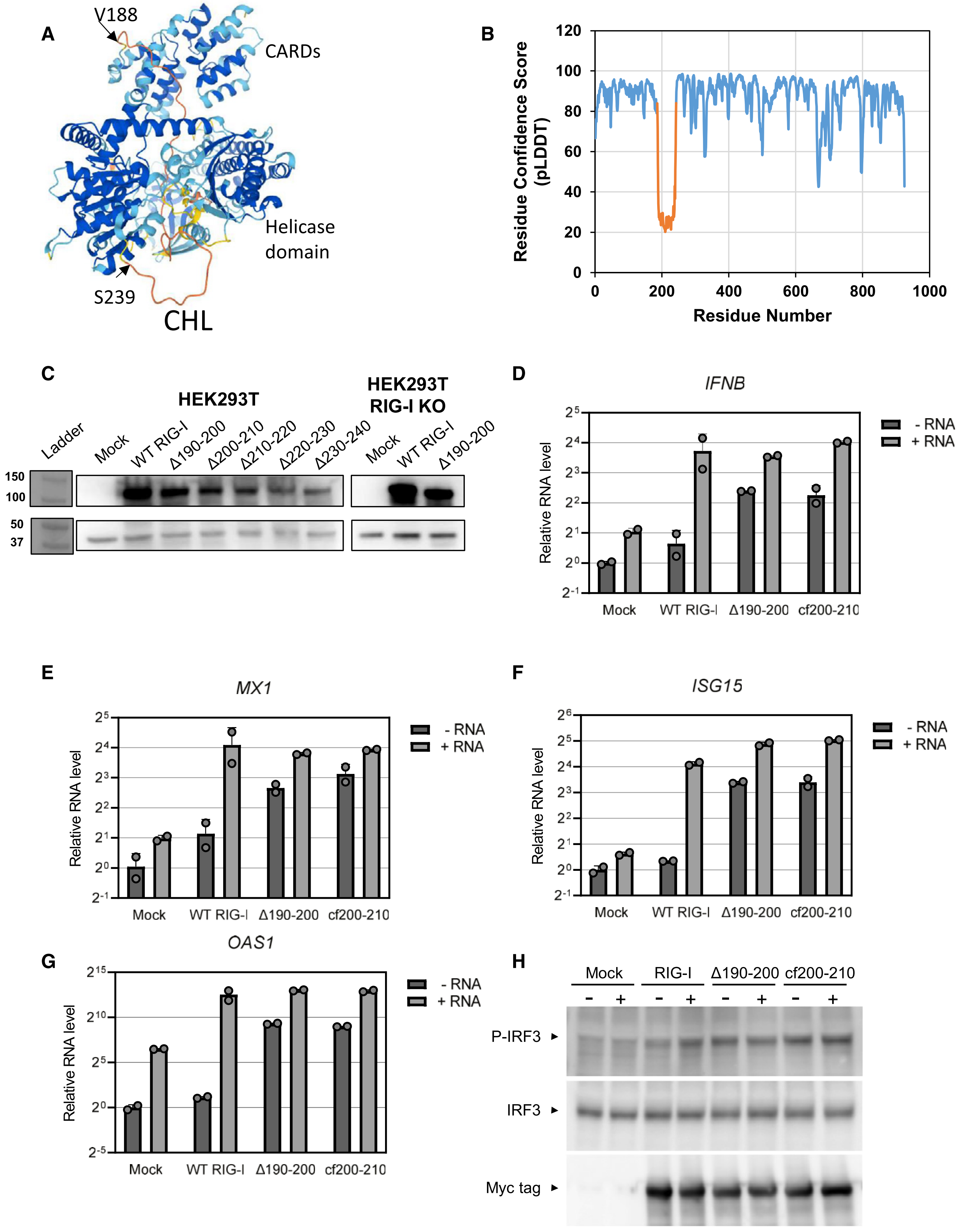

Figure EV1.

© 2022 The Authors

Figure EV2. Hydrogen–Deuterium Exchange Mass Spectroscopy (HDX) heatmap comparing WT RIG-I and Δ190–200 RIG-I.

The average ΔD_2O% ± standard deviation between WT RIG-I and Δ190–200 RIG-I across all HDX time points. HDX Workbench colors each peptide according to the smooth color gradient HDX perturbation key shown in each indicated figure. Average ΔD_2O% between −5% and 5% are considered non-significant and are colored gray.

A WT RIG-I versus Δ190-200 RIG-I, no RNA

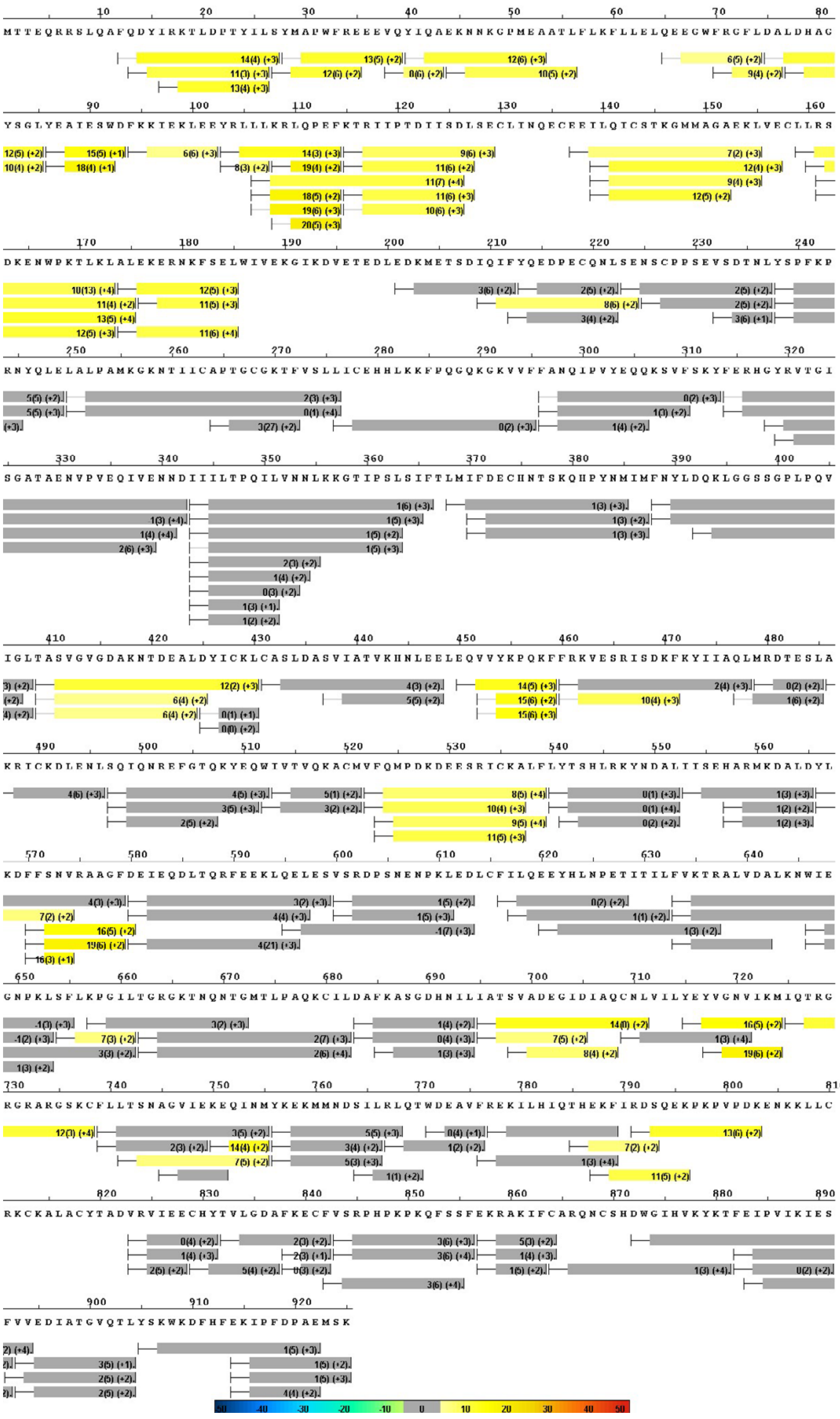

Figure EV2.

© 2022 The Authors

A

MDA5

Theoretical pI = 3.44

```
                                              *      *     *          *
Human    :  177 WFSAFLNVLRQTGNNELVQELTGSDCSESN--AEIENLSQVDGPQVEEQLLSTTVQPNLE
Macaque  :  177 WFSAFLDVLRQTGNDELVQELTGTDCSESN--AEIENLSQDDGPQVEEQLLSTTVQPNLE
Mouse    :  177 WFSTFLDVLRQTGNDALFQELTGGGCPEDN--TDLANSSHREGPAANECLLPAVDESSLE
Mallard  :  175 WFSPFLVALRETQHEDLADDLSGNTGGKENKGNGVKNSTNKETEAASQPGHSVVKDLKQQ
Zebrafish:  177 WFSKFLQALEDTEHRDLMRELRGEPCEGDE---GMSVDSSDAQAEVKDTGAVRRNSMSSP

                 *       *    *    *     * *    *     **         *      *
Human    :  235 KEVWG---MENNSSESSFADSSVVSESDTSLAEGSVSCLDESLGHNSNMGSDSGTMGSDS
Macaque  :  235 KEVWG---MENNSSESSFADSSVVSESDTSLAEGSVSCLDESLGHNSNMGSDSGTMGSDS
Mouse    :  235 AEAWN---VDDILPEASCIDSSVTTESDTSLAEGSVSCFDESLGHNSNMGRDSGTMGSDS
Mallard  :  235 ENLNGGSVSENGVLETSVEKNSVVLESDVATEDRSVSNMNENLGQSS--------TTSNS
Zebrafish:  234 PPSFT---PNSSLQSEDLDDSVDSSVLGVEAGNRGVEMYEDGEEKEEK------DLKEDE

                ***
Human    :  292 DEENVAAR-ASPEPELQLRPYQMEVAQPALEGKNIIICLPTGSGKTRVAVYIAKDHLDKK
Macaque  :  292 DEENVAAR-ASPEPELQLRPYQMEVAQPALEGKNIIICLPTGSGKTRVAVYIAKDHLDKK
Mouse    :  292 DESVIQTKRVSPEPELQLRPYQMEVAQPALDGKNIIICLPTGSGKTRVAVYITKDHLDKK
Mallard  :  287 DEDEMESR-ASPEPDLILRDYQMEVAKPALNGENIIICLPTGSGKTRVAVYITKDHLDKK
Zebrafish:  285 DDDSSASK-----REIKLRDYQMEVARPALEEKNIIVCLPTGSGKTRVAVFITKEHLERE
```

B

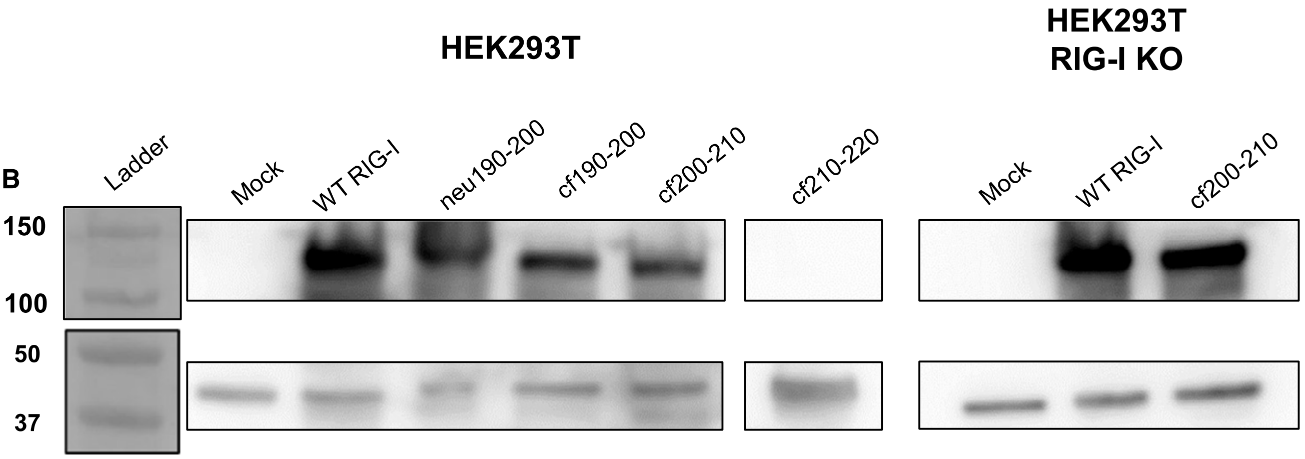

Figure EV3. CHL in MDA5 and Western analysis of RIG-I expression in the reporter assays of the charge flip CHL mutants.

A RIG-I Like Receptor MDA5 has an electronegative CARD2-helicase linker region similar to RIG-I. MDA5 sequences from five homologs spanning human to bony fish were aligned and analyzed as in Fig 3A. Black line above the sequence indicates the MDA5 CHL. All glutamate and aspartate residues are colored red, and lysines are colored blue. Asterisks indicate when a negative charge, either glutamate or aspartate, is conserved at that position in three out of five homologs tested.

B Western blot of signaling data shown in Fig 3 confirm protein expression, as in Fig EV1. In each experiment, pcDNA3.1 myc-tagged RIG-I constructs (approximately 108 kDa) were recognized with a primary α-Myc antibody. β-actin (approximately 42 kDa) was used as a normalization control. Cf210–220 was not included in cell signaling data as it did not express. Numbers (left) refer to molecular weight in kDa.

© 2022 The Authors

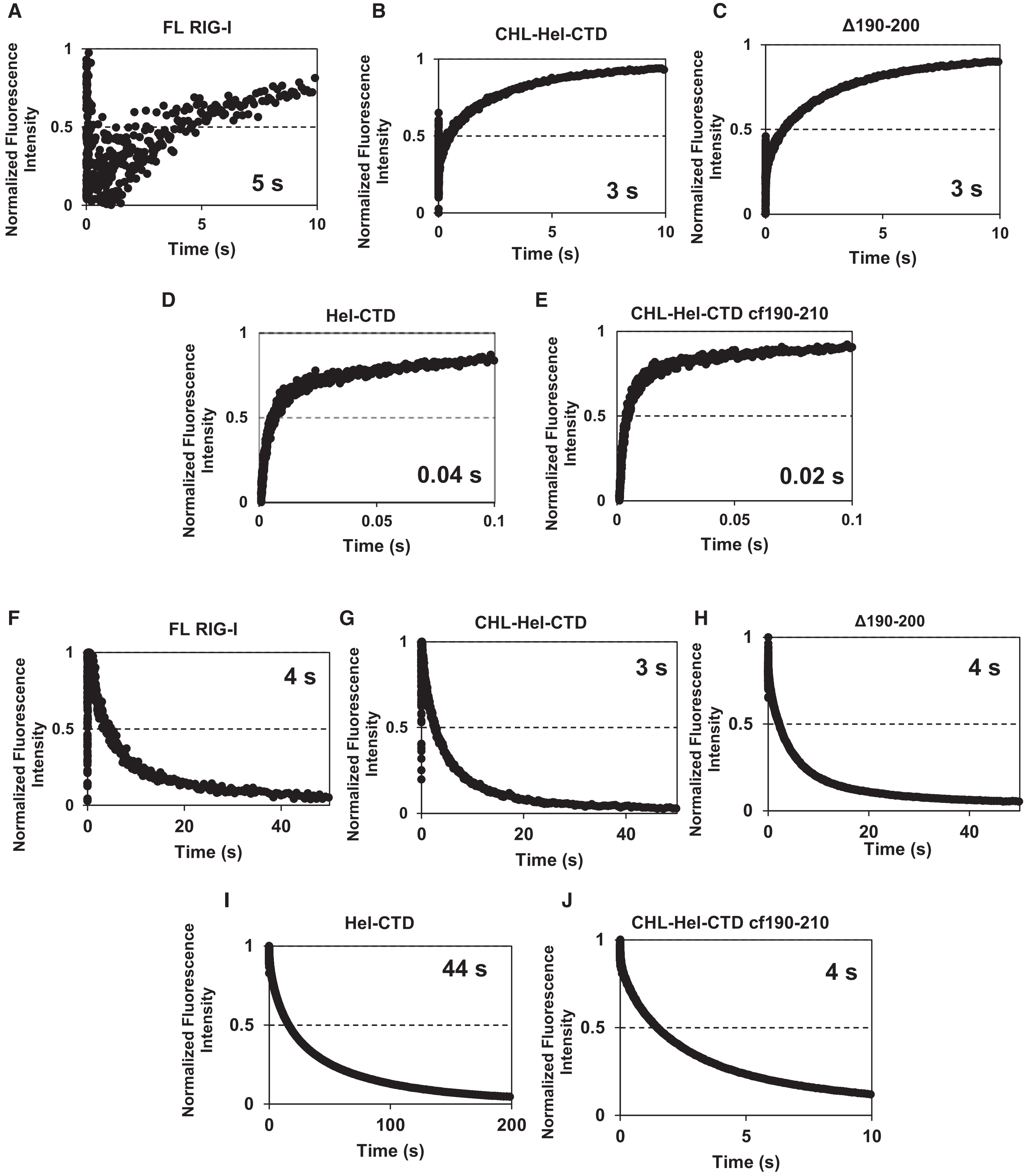

Figure EV4. Stopped-flow kinetic traces of association and dissociation reactions of ds26 stem with various RIG-I constructs.

A–E Thumbprint graphs showing ds26 stem RNA association kinetics to various RIG-I constructs. Each curve is an average of at least 6 individual traces ($n = 6$).

F–J Thumbprint graphs showing ds26 stem RNA dissociation kinetics from various RIG-I constructs. Numbers are the average association or dissociation times calculated from the amplitude and rate constants shown in Appendix Table S2 using Equation 3. Each curve is an average of at least six individual traces ($n = 6$).

© 2022 The Authors

Figure EV5. RNA binding studies of RIG-I constructs.

A Bar chart comparing each protein construct's ATPase V_{max} tested simultaneously against either 5' ppp ds27 from Fig 5 or No RNA. Note that no construct could hydrolyze ATP in the absence of bound RNA, typical of RIG-I, and as such it is not shown. 10nM of protein was incubated with 100 nM of 5' ppp ds27 RNA in all cases, and time course was performed with points taken at 0", 30", 60" and 90" (mechanical replicate, $n = 3$). Bars represent calculated V_{max} values. Error bars represent standard error of fit.

B Table shows the RNA $K_{D,app}$ values shown as bars in Fig 5. Standard errors are the average standard errors of fit for two binding trials, each trial with mechanical replicates of $n = 3$.

C–E Bar charts showing the $K_{D,app}$ of CHL-Hel-CTD and Δ190–200 RIG-I from titrations measuring ATPase as a function of RNA concentration. RNA schematic: Black: RNA, blue: DNA. Bar represents the mean $K_{D,app}$ value of two independent RNA binding experiments, while dots indicate each independent RNA binding experiment. Each independent binding experiment had three time points (mechanical replicate, $n = 3$).

F Table shows the energetic contributions of CHL and CHL-CARDs to autoinhibiting RNA binding, calculated using $K_{D,app}$ values in D and Equations 7, 8, and 9.

© 2022 The Authors

A

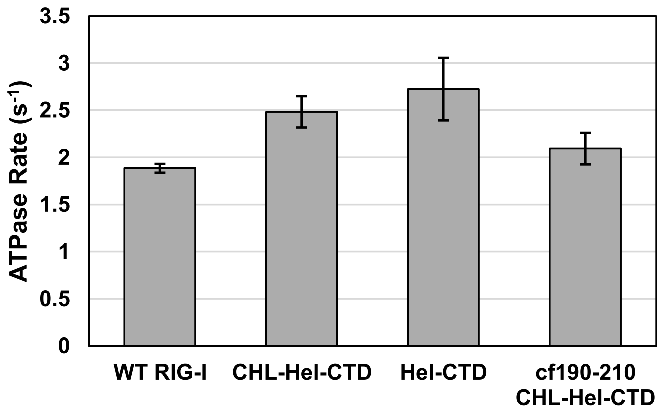

B

$K_{D,\ app}$ (nM)	5’ppp RNA	3'ovg RNA	5'ovg RNA	stem RNA
**WT RIG-I**	7.4 ± 1.2	23.1 ± 5.5	105 ± 32	1250 ± 183
**CHL-Hel-CTD**	0.8 ± 0.2	3.6 ± 2.9	4.9 ± 0.8	42.0 ± 13.6
**Hel-CTD**	1.0 ± 0.6	0.27 ± 0.18	0.4 ± 0.4	2.0 ± 0.8
**cf190-210 CHL-Hel-CTD**	0.5 ± 0.2	1.0 ± 1.4	0.80 ± 0.85	2.8 ± 2.1

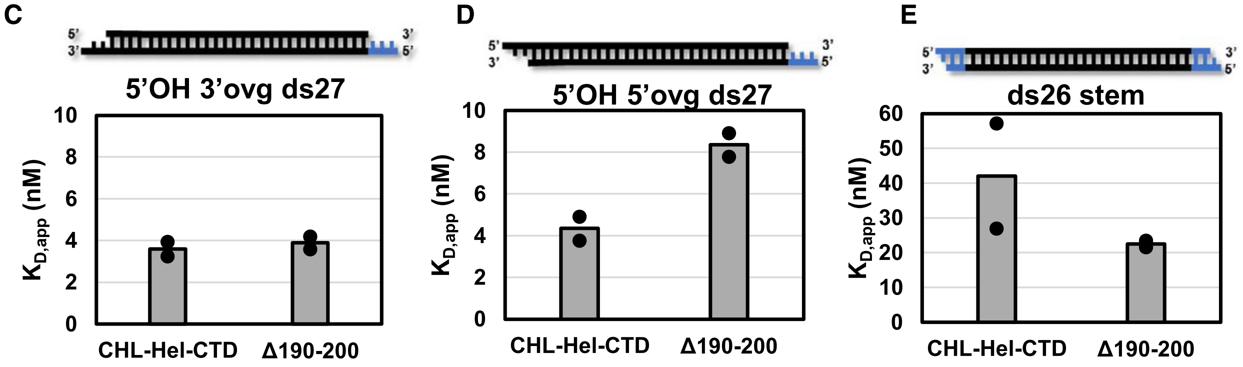

F

Energetic contribution to autoinhibition (ΔΔG)	5‘ppp RNA (ΔΔG) *kcal/mol*	3'ovg RNA (ΔΔG) *kcal/mol*	5'ovg RNA (ΔΔG) *kcal/mol*	stem RNA (ΔΔG) *kcal/mol*
**CHL**	0.142	-1.42	-1.482	-1.622
**CHL-CARDs**	-1.21	-2.432	-3.252	-3.91

Figure EV5.

 © 2022 The Authors
