# Supplementary material for: The intrinsically disordered CARDs‐Helicase linker in RIG‐I is a molecular gate for RNA proofreading
Source: EMBO J. 2022 Apr 19;41(10):e109782. doi: 10.15252/embj.2021109782 (PMC9108607; doi:10.15252/embj.2021109782)
Supplement: Supplementary file 3 [file EMBJ-41-e109782-s003.pdf]

THE EMBO JOURNAL

The intrinsically disordered CARDs-Helicase linker in RIG-I is a molecular gate for RNA proofreading

Brandon Schweibenz, Swapnil Devarkar, Mihai Solotchi, Candice Craig, Jie Zheng, Bruce Pascal, Samantha Gokhale, Ping Xie, Patrick Griffin, and Smita Patel
DOI: 10.15252/embj.2021109782

Corresponding author(s): Smita Patel (patelss@rutgers.edu)

Review Timeline:

Submission Date:	22nd Sep 21
Editorial Decision:	9th Nov 21
Revision Received:	5th Feb 22
Editorial Decision:	3rd Mar 22
Revision Received:	28th Mar 22
Accepted:	4th Apr 22

Editor: Stefanie Boehm

Transaction Report:

(Note: With the exception of the correction of typographical or spelling errors that could be a source of ambiguity, letters and reports are not edited. Depending on transfer agreements, referee reports obtained elsewhere may or may not be included in this compilation. Referee reports are anonymous unless the Referee chooses to sign their reports.)

Thank you again for submitting your manuscript to The EMBO Journal, as well as sending the preliminary point-by-point response to the referee comments. Based on this response and the experiments and revision of the manuscript you propose, we would now like to invite you to prepare and submit a revised version.

In this revised manuscript, please address the referee comments as proposed and revise the text and figures as appropriate, as well as providing a detailed response to each comment. Specifically, as we discussed, it will be important to repeat the key experiments with 5'ppp dsRNA ligand to address both referees' concerns regarding the use of polyI:C for RIG-I induction, as well as adding the appropriate controls to these experiments (ref #1- point 1, ref #2- point 1). In addition, please address referee #2's concerns regarding the involvement of endogenous RIG-I experimentally, as well as providing additional experimental proof for Type I IFN induction (ref #2- point 1, 2). Furthermore, please respond to referee #2's point 3 in detail and revise the text as needed.

Please note that it is our policy to allow only a single round of major revision. Acceptance depends on a positive outcome of a second round of review and therefore on the completeness of your responses included in the next, final version of the manuscript. It is thus important to clarify any questions or issues with us as soon as possible during the revision.

Thank you for the opportunity to consider your work for publication. I look forward to receiving your revised manuscript.

Referee #1:

In this study Schweibenz et al use cell-based assays, biochemical assays, and hydrogen/deuterium exchange coupled to mass spectrometry (HDX-MS) to dissect the contribution of the 56-amino acid CARDs-Helicase Linker (CHL) of RIG-I in RNA ligand binding, signaling activation, and viral RNA vs self RNA discrimination. The authors tested the effect of five different 11-amino acid deletions in the CHL on RIG-I-dependent signaling using an HEK293T cell-based IFN-beta luciferase reporter assay. Three of the deletions were found to increase RIG-I signaling without stimulation with PAMP-like RNA, and to a lesser extent with poly(I:C) stimulation. Substitution of the abundant negatively charged residues in the CHL with neutral or flipped-charge residues instead of deletion has similar effects on signaling, albeit more modest in their amplitude. The authors also examined the effect of their CHL mutations on binding (affinities and rates) to a dsRNA ligand lacking 5'-di/triphosphate (and hence non-PAMP-like). The CHL mutations were found to increase dsRNA binding affinity, and the binding on-rate significantly, consistent with a role of the CHL in competing with cellular dsRNA binding to prevent autoinflammation. Lastly, the authors used an ATPase assay to measure the RNA Kd values for RNA ligands with different types of 5' and 3' overhangs, showing that binding to non-PAMP-like RNAs is uniformly weakened by the CHL.

This study sheds light on the function of the CHL of RIG-I in RNA ligand discrimination. The potential functions of this linker have been overlooked so far, probably because its amino acid sequence is poorly conserved, and it appears to lack a stable fold. However, this study convincingly shows that the enriched negatively charged residues in the CHL compete with backbone RNA phosphates for binding to RIG-I, providing the innate immune receptor with an additional layer of discrimination between viral and cellular dsRNA. The primary concerns are that the authors fail to integrate virus-like dsRNAs and previously discovered RNA proofreading mechanisms into the study, and the partially incorrect interpretation of the HDX-MS data.

Concerns (listed from most to least concerning)

1. None of the ligands used effectively mimic virus-like RIG-I ligands or bind to the RIG-I CTD. PolyI:C is a poor mimic of virus-like RNA for RIG-I as it contains few if any 5'-di/triphosphorylated blunt-ends. Hence the study is missing positive controls with virus-like, 5'-di/triphosphorylated, blunt-ended dsRNAs. Specifically, in the RNA binding and ATPase experiments (p. 6-8, Figs. 4-5) the author's claim of the CHL contributing to RNA binding specificity would be strengthened if they showed how binding of ligands with 5'-di/triphosphates is or is not affected by CHL mutations. One would expect that virus-like RNAs are most effective as outcompeting the CHL for binding to the RIG-I helicase domains than the ligands tested in this study, and it seems relevant and useful to confirm this experimentally. As the manuscript currently stands, characterizing the contribution of the CHL to competing with cellular RNA binding as a "proofreading mechanism" seems like an overstatement as the authors have not yet shown how the CHL functions with cognate virus-like ligands in their cell-based and biochemical assay readouts.

2. Related to the point above, the authors previously uncovered an ATPase-dependent "translocation-throttling" proofreading mechanism in RIG-I, yet this study does not mention this mechanism, or how the additional layer of ligand discrimination provided by the CHL relates to the ATPase-dependent proofreading. Can the effect of CHL mutations on ATPase activity be used to make inferences on the interplay between ATP- and CHL-dependent proofreading? Can fluorescent or FRET readouts be added to dissect the role (or lack thereof) of CHL in RIG-I oligomerization? The model proposed here (Fig. 6) should be integrated with other known proofreading mechanisms and specifically Devarkar et al (2016).

3. HDX-MS: increased H/D exchange is a measure of solvent exposure, not peptide backbone flexibility as stated (p. 4). Increased backbone flexibility can increase H/D exchange indirectly by increasing solvent exposure of the more flexible region,

but perfectly rigid backbones can also have increased H/D exchange as a result of being exposed. Indeed, this is most likely why the CARDs have increased H/D exchange in the CHL deletion mutants: the shorter CHLs are too short to allow CARDs to reach their preferred binding side on Hel2i, causing some residues normally buried at the CARD-Hel2i interface to remain exposed and undergo H/D exchange.

4. It is not surprising (as stated on p. 3) that deletions in the CHL increased signaling, as one could naturally expect shortening of the CHL to render the linker too short to be compatible with the auto-inhibitory CARD-Hel2i interaction. In other words, it is an expected but noteworthy result that CHL deletions prevent the CARDs from binding Hel2i and properly maintain RIG-I in its autoinhibitory conformation. Results, p. 3 first paragraph: "To our surprise" should be deleted.

5. Mutations in the CHL would be expected to elevated baseline RIG-I signaling in the absence of viral RNA, potentially causing autoinflammation. Do any (autoinflammatory or other) disease-associated mutations map to the CHL of RIG-I?

6. Introduction: where Oates et al (2013) is cited the Alphafold2 structure prediction should also be mentioned. P. 8. Please show the Alfafold2 model of the CHL somewhere.

7. The Discussion repeats several points already stated or discussed earlier in the paper. The Discussion should be shortened by about one-third (particularly top of p. 9) and be made less repetitive and speculative (particularly bottom of p. 9 and top of p. 10).

8. Statements such as "The discovery... represents an exciting, new and unique opportunity" (p. 20) should be deleted throughout the text. Readers will form their own opinions and primacy statements are best avoided.

9. Typos: P. 3 line 3: change "discovery" to "discovered"; P. 6 last line: delete "more".

10. Please add line numbers and text-embedded figures and figure legends for easier review.

Referee #2:

This manuscript describes an interesting and new observation that adds to our understanding of how RIG-I distinguishes between 'normal' RNAs and immunostimulatory RNAs: a disordered linker (called here CHL) prevents RNAs from binding to the helicase domain, unless such RNAs are first bound by the CTD. This finding is of high interest to specialists in the field of nucleic acid sensing. Overall, the manuscript is well written and the conclusions are supported by the data. In particular, the HDX-MS and biochemical (binding and ATPase) data are convincing. Unfortunately, functional experiments are limited to a single rather artificial assay. The manuscript would be strengthened (and its wider appeal increased) by further work in this direction, as detailed below.

Major points

1. The HEK293T cells used for the reporter assay do not express high levels of RIG-I and MDA5 at baseline. However, in the 20 hour timeframe of the experiment, transfected pI:C will lead to type I IFN production, which in turn via IFNAR signalling is likely to induce endogenous RIG-I and MDA5 expression. This is an important caveat as pI:C activates both these receptors. As such, the readout at the end of the experiment is a combination of signalling via the overexpressed RIG-I mutants, endogenous RIG-I and endogenous MDA5. This poses difficulties in interpreting the data, in particular because different levels of IFN induction are seen with the mutants; hence, upregulation of the endogenous receptors will likely be different. I suggest to circumvent this issue by using RIG-I KO cells and a more selective RIG-I agonist: 5'PPP dsRNA. The former cells are widely used in the field and the latter is commercially available (Invivogen). Alternatively, if the authors wish to use pI:C, RIG-I + MDA5 double KO cells should be used.
2. Type I IFN induction should be measured by additional readouts (in addition to the luciferase reporter). These should include: measurement of IFN and ISG mRNA levels by RT-qPCR, measurement of IFNs by ELISA and measurement of ISGs by western blots. Such data would strengthen the conclusions. In addition, p-IRF3 and p-TBK1 western blots may be informative.
3. In addition to using synthetic transfected RNAs as stimuli, the authors should confirm their findings using a virus infection model. Sendai virus would be ideal; it is commercially available (ATCC), is a biosafety level 1 virus, and activates RIG-I specifically.
4. Fig 1D and the statement on page 4 ("We observed a normal PAMP-stimulated signaling response from CHL mutants with Poly I:C") are misleading. For WT RIG-I, pI:C stimulation versus no RNA upregulates the reporter by ca 6-fold (2.5 to 15). However, for the first three mutant RIG-I constructs, this effect is less than 2-fold (15 to 27). Dose-titrations are be needed to properly draw comparative conclusions.
5. Fig 3A and EV3. Please provide the theoretical pI for the CHLs of all species shown and also for MDA5 CHL.
6. Fig 4. Please briefly explain the protein purification strategy and provide evidence that all four RIG-I proteins are of equal purity.
7. The experiments in Figs 5 and EV5 need to be repeated at least once more to allow statistical comparison between different constructs.

8. In Fig 5, it would be informative to include 5-PPP (or 5-PP) RNAs in the analysis as these are the most relevant RIG-I triggers in virus infected cells.
9. Does LGP2 have a CHL like region?
10. Are there any naturally occurring mutations in the CHL linker, perhaps linked to diseases such as Singleton-Merten syndrome?

Minor points
11. Abstract. First line. Please change 'the first line' to 'a first line' to avoid over-generalisation.
12. Page 2. Please provide appropriate references for the statement: "Unlike CTD, the helicase domain lacks RNA specificity and hence can bind non-specific RNAs."
13. The graphs in Fig 1C and EV1 seem identical. I suggest to incorporate the western blot from EV1 into Fig 1. Similarly, I suggest to incorporate the western blot from EV3B (which should be straightened) into Fig 3C/D. Please use consistent nomenclature (presumably neu190-200 and GST 190-200 are the same construct).
14. Page 6, figure call-out: Figure 4 has no panel D.

We thank the reviewers for the careful evaluation of our work and pleased that they are generally excited with our findings of a new regulatory region in RIG-I involved in RNA proofreading. We have taken all the comments and suggestions very seriously and addressed the concerns by conducting a large number of new experiments that have further strengthened our main conclusions. The manuscript text was edited for clarity and changes describing the new experiments are marked in red. The list below summarizes the changes:

1. We have slightly edited the title to make it more specific "The intrinsically disordered CARDs-Helicase linker in RIG-I is a molecular gate for RNA proofreading".

2. Changes in Figure 1 and EV1:

   a. We have replaced the IFN reporter results of the PolyIC in the previous version with the 5'ppp RNA data (Figures 1C and 1D).

   b. We have conducted new experiments with 293T RIG-I KO cells and these are added in Figure 1E and 1F.

   c. In EV1, we show the Alphafold RIG-I model, new qRT-PCR results of IFN response gene induction, and Westerns to demonstrate pIRF3 expression.

3. Changes in Figure 2 – None

4. Changes in Figure 3:

   a. We have replaced the PolyIC reporter data with the 5'ppp RNA data set (Figure 3C and 3D)

   b. The new experiments with charge flip mutants in 293T RIG-I KO cells are shown in Figure 3E and 3F.

5. Changes in Figure 4:

   a. We have carried out new RNA binding studies of the RIG-I constructs with 5'ppp RNA (Figure 4C, 4D, 4E).

6. Change in Figure 5 and EV5:

   a. We added new ATPase-based RNA binding studies of 5'ppp RNA (Figure 5D).

b. EV5 includes tables of RNA K_D values and energetic contributions.

7. Changes in Figure 6:

   a. We have added a more complete model showing how RIG-I uses various layers of RNA proofreading in self and non-self discrimination. We have removed the modeling data (originally EV6, only 5 EV figures are accepted).

8. Appendix Figure S1 shows the SDS PAGE of RIG-I mutant proteins and the new EMSA results showing 5'ppp RNA binding and dimer formation with ATP.

9. Appendix Figure S2 shows the rate constants of stem RNA association and dissociation from stopped flow studies.

Referee #1 (Report for Author)

In this study Schweibenz et al use cell-based assays, biochemical assays, and hydrogen/deuterium exchange coupled to mass spectrometry (HDX-MS) to dissect the contribution of the 56-amino acid CARDs-Helicase Linker (CHL) of RIG-I in RNA ligand binding, signaling activation, and viral RNA vs self RNA discrimination. The authors tested the effect of five different 11-amino acid deletions in the CHL on RIG-I-dependent signaling using an HEK293T cell-based IFN-beta luciferase reporter assay. Three of the deletions were found to increase RIG-I signaling without stimulation with PAMP-like RNA, and to a lesser extent with poly(I:C) stimulation. Substitution of the abundant negatively charged residues in the CHL with neutral or flipped-charge residues instead of deletion has similar effects on signaling, albeit more modest in their amplitude. The authors also examined the effect of their CHL mutations on binding (affinities and rates) to a dsRNA ligand lacking 5'-di/triphosphate (and hence non-PAMP-like). The CHL mutations were found to increase dsRNA binding affinity, and the binding on-rate significantly, consistent with a role of the CHL in competing with cellular dsRNA binding to prevent autoinflammation. Lastly, the authors used an ATPase assay to measure the RNA Kd values for RNA ligands with different types of 5' and 3' overhangs, showing that binding to non-PAMP-like RNAs is uniformly weakened by the CHL.

This study sheds light on the function of the CHL of RIG-I in RNA ligand discrimination. The potential functions of this linker have been overlooked so far, probably because its amino acid sequence is poorly conserved, and it appears to lack a stable fold. However, this study convincingly shows that the enriched negatively charged residues in the CHL compete with backbone RNA phosphates for binding to RIG-I, providing the innate immune receptor with an additional layer of discrimination between viral and cellular dsRNA. The primary concerns are that the authors fail to integrate virus-like dsRNAs and previously discovered RNA proofreading mechanisms into the study, and the partially incorrect interpretation of the HDX-MS data.

Concerns (listed from most to least concerning)

1. None of the ligands used effectively mimic virus-like RIG-I ligands or bind to the RIG-I CTD. PolyI:C is a poor mimic of virus-like RNA for RIG-I as it contains few if any 5'-di/triphosphorylated blunt-ends. Hence the study is missing positive controls with virus-like, 5'-di/triphosphorylated, blunt-ended dsRNAs. Specifically, in the RNA binding and ATPase experiments (p. 6-8, Figs. 4-5) the author's claim of the CHL contributing to RNA binding specificity would be strengthened if they showed how binding of ligands with 5'-di/triphosphates is or is not affected by CHL mutations. One would expect that virus-like RNAs are most effective as outcompeting the CHL for binding to the RIG-I helicase domains than the ligands tested in this study, and it seems relevant and useful to confirm this experimentally. As the manuscript currently stands, characterizing the contribution of the CHL to competing with cellular RNA binding as a "proofreading mechanism" seems like an overstatement as the authors have not yet shown how the CHL functions with cognate virus-like ligands in their cell-based and biochemical assay readouts.

Both reviewers suggested that we conduct the IFN promoter reporter and RNA binding assays with a RIG-I PAMP. We have now repeated all the cell signaling assays with a 5'ppp blunt-ended 39 bp RNA in parallel with the No RNA experiments and replaced the original polyIC data with the 5'ppp ds39 data (Figures 1C and 1D, see below). These new data show that the CHL mutants are not defective in responding to the viral like, 5'ppp ds39 RNA, while confirming the hyperactive basal signaling activity of the CHL mutants in the 190-200 region.

We have carried out additional reporter assays in 293T RIG-I KO cells (Figures 1E and 1F, see below), which confirms that the hyperactive basal signaling is not due to activation of endogenous RIG-I. We also show that the CHL mutants activate many IFN response genes (qRT-PCR and Westerns in Figure EV1) (see response to reviewer 2, Q.1.)

These new cell signaling experiments strengthen our main claim that CHL is a regulatory region that autoinhibits RIG-I, keeping the CARDs in a silent signaling state in the absence of PAMP RNA.

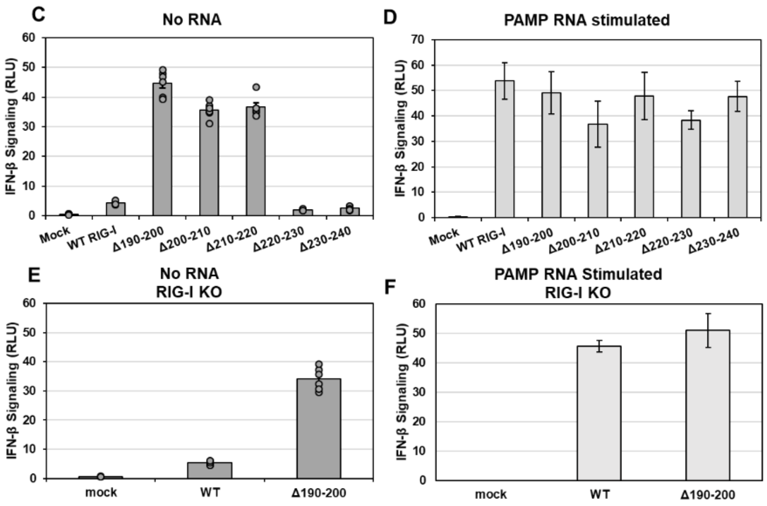

Panels from Figure 1

1C. IFN response of mock-transfected, WT and CHL RIG-I mutants measured by luciferase reporter assays in HEK293T cells under No RNA conditions and reported as Relative Luciferase Units (RLU). Each condition was performed 3 times by 2 independent workers (n=6), and individual trails are plotted. Error bars reflect standard error.

1D. 5'ppp ds39 stimulated IFN response. Note that the PAMP RNA stimulated graph reflects the background; No RNA signal from panel C subtracted from the total amount of signaling after RNA transfection. Error bars from 6 trials were calculated by the sum of squares methods from subtracted graphs.

1E. IFN response of WT and Δ190-200 performed as in (C), except in HEK293T RIG-I KO cells.

1F. 5'ppp ds39 stimulated IFN response of WT and Δ190-200 as in (D) in HEK293T RIG-I KO cells.

To address the second concern, we have measured the 5'ppp RNA K_D values, and these new RNA binding data are added in Figures 3 and 4. As expected, the results show that the 5'ppp dsRNA is better at outcompeting the CHL than the non-PAMP RNAs. For example, we show that CHL and CARD2:Hel2i interface inhibit stem RNA binding by ~ 700-fold whereas 5'ppp RNA

binding is inhibited by ~ 5-fold only (see figure below, note log y-axis in panel A and linear in panel D). These provide strong support for the RNA proofreading function of the CHL. Panels of Figure 4 are shown in response to reviewer 2 Q.8.

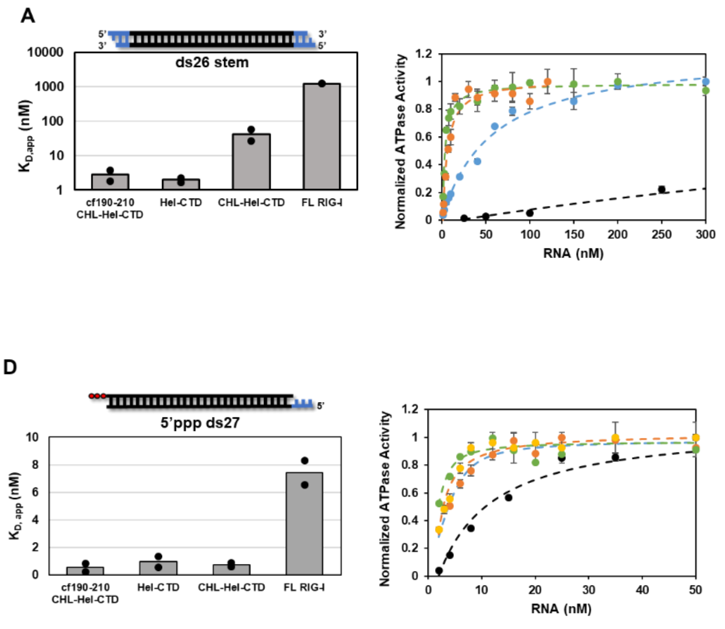

Panels from Figure 5

5(A) –(D) Bar charts (left) and ATPase titrations (right) comparing the $K_{D,\ app}$ values of (A) ds26 stem RNA and (D) 5'ppp ds27 for each of the RIG-I constructs. Individual $K_{D,app}$ values from two independent titrations are shown in the bar charts. Representative titration experiments show the ATPase activity plotted against RNA concentration for each of the four RIG-I constructs. Each curve was normalized to the highest measured ATPase activity in each assay. WT RIG-I (black filled circles), CHL-Hel-CTD (blue filled circles), Hel-CTD (green filled circles), and CHL-Hel-CTD cf190-210 (orange filled circles). Error of fit from three time points of reaction are shown for each point. Hel-CTD and cf190-210 CHL-Hel-CTD titrations were fit using a quadratic equation (Equation 6), while all other RNAs tested were fit using a hyperbola (Equation 5) to obtain the KD, app values (Figure EV5A). The $K_{D,app}$ values are tabulated in Figure EV5A.

2. Related to the point above, the authors previously uncovered an ATPase-dependent "translocation-throttling" proofreading mechanism in RIG-I, yet this study does not mention this mechanism, or how the additional layer of ligand discrimination provided by the CHL relates to the ATPase-dependent proofreading. Can the effect of CHL mutations on ATPase activity be used to make inferences on the interplay between ATP- and CHL-dependent proofreading? Can fluorescent or FRET readouts be added to dissect the role (or lack thereof) of CHL in RIG-I oligomerization? The model proposed here (Fig. 6) should be integrated with other known proofreading mechanisms and specifically Devarkar et al (2016).

We thank the reviewer for making this suggestion. We have modified Figure 6 to include the three layers of RNA proofreading that RIG-I uses to discriminate between self and non-self RNAs, discovered from this and published works. The model (see below) shows how RIG-I's CTD, the CHL gate, and the ATPase activity work together to antagonize self RNAs and form signaling active complexes on PAMP RNA.

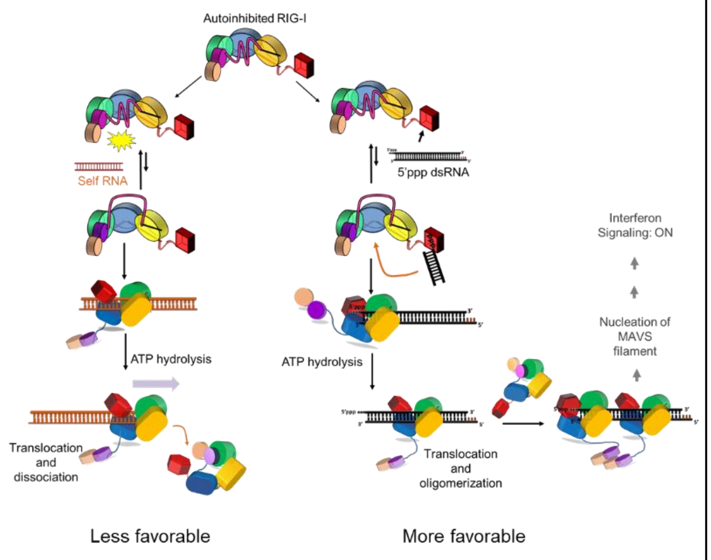

Figure 6. RNA proofreading mechanisms of RIG-I. The model shows how RIG-I discriminates between self and non-self RNAs using layers of RNA proofreading. PAMP RNA binding on the right contrasts with self-RNA binding on the left. In the resting state of RIG-I, the Hel2i sequesters the CARDs in the autoinhibitory conformation and the CHL stabilizes this conformation while blocking RNA access into the helicase domain. In the autoinhibitory state, the CTD can freely access RNAs and detect PAMP RNA. The high selectivity of the CTD for PAMP RNA over self RNAs provides the first layer of RNA proofreading. The CHL gate provides the second layer of RNA proofreading. Transient opening of the CHL gate disfavors self RNA binding (in the left). However, PAMP RNAs can access the RNA binding site of the helicase domain when CHL gate opens transiently because they are held close to the helicase domain through prebinding to CTD. RNA binding induces conformational changes in the helicase domain that expose the CARDs. Due to favorable CTD interactions, CARDs exposure is more efficient on PAMP RNA than on self-RNA. The ensuing ATP hydrolysis checks for erroneous RNA binding by promoting RIG-I to translocate and dissociate from self-RNAs. The CTD interactions throttle RIG-I translocation on PAMP RNA and

Is there an interplay between the CHL- and ATP-dependent proofreading mechanisms, and does CHL play a role in RIG-I oligomerization? Our results suggest that the CHL- and ATPase –mediated proofreading mechanisms are independent. The CHL mutants are competent in ATP hydrolysis in the presence of RNA (Figure 5, EV5A, see below). Thus deletions in CHL do not affect the ATPase activity, so presumably the mutants can use the ATPase activity to proofread RNAs. But this proofreading mechanism alone, without the CHL's gating mechanism, is not enough to dissociate non-PAMP RNAs.

The EMSA shows that CHL mutants can dimerize on the 5'ppp ds27 RNA in the presence of ATP at 1:1 amounts of protein:RNA (Appendix Figure S1B-C, see below). These results suggest that CHL can oligomerize, which is consistent with their ability to activate RIG-I signaling.

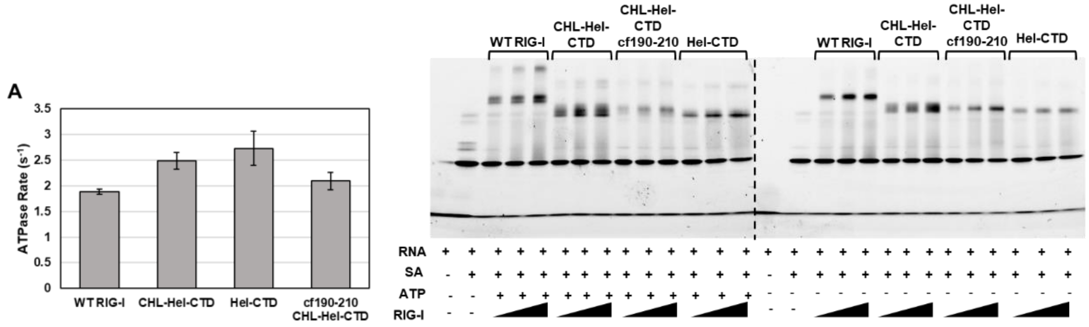

Panels from Figure EV5 and Appendix Figure S1

(A) Bar chart comparing each protein construct's ATPase Vmax tested simultaneously against 5' ppp ds27

(C) EMSA shows binding and oligomerization of various RIG-I constructs on 5'ppp ds27 RNA used in Figure 4 (with streptavidin to block binding from the non-ppp end) in the presence and absence of ATP. RIG-I was incubated at either 30 nM, 50 nM, or 70 nM with 50 nM of 5'ppp ds27 3'DY547 with distal 5' biotin and 55 nM of monovalent streptavidin (SA) for 60 minutes at 4°C. 2 mM ATP was added, where indicated, 15 minutes before loading reactions into the gel.

3. HDX-MS: increased H/D exchange is a measure of solvent exposure, not peptide backbone flexibility as stated (p. 4). Increased backbone flexibility can increase H/D exchange indirectly by increasing solvent exposure of the more flexible region, but perfectly rigid backbones can also have increased H/D exchange as a result of being exposed. Indeed, this is most likely why the CARDs have increased H/D exchange in the CHL deletion mutants: the shorter CHLs are too short to allow CARDs to reach their preferred binding side on Hel2i, causing some residues normally buried at the CARD-Hel2i interface to remain exposed and undergo H/D exchange.

We agree that the increased H/D exchange can be due to exposure of flexible or rigid region, and accordingly we have revised p.4 statements. Regarding the comment on shorter CHL preventing CARDs from reaching Hel2i, see our response to Q. 4, below.

4. It is not surprising (as stated on p. 3) that deletions in the CHL increased signaling, as one could naturally expect shortening of the CHL to render the linker too short to be compatible with the auto-inhibitory CARD-Hel2i interaction. In other words, it is an expected but noteworthy result that CHL deletions prevent the CARDs from binding Hel2i and properly maintain RIG-I in its autoinhibitory conformation. Results, p. 3 first paragraph: "To our surprise" should be deleted.

The reason we were surprised to see that CHL deletions constitutively activated RIG-I signaling is because we had expected that shortening the CHL would disrupt CARDs:CARDs interactions and lower RIG-I signaling. This is explained more clearly in the introduction.

Introduction p.3, we added:

"No known functions were associated with the CHL, except that it tethers the CARDs to the helicase and appropriately long CHL might be important for enabling intermolecular CARDs:CARDs interactions. Therefore, we were surprised to find that small deletions in CHL did not impair RIG-I signaling and instead constitutively activated RIG-I signaling without the addition of PAMP RNA."

For the following reason, we believe that the loss of key interactions involving the negative charges in CHL rather than CHL shortening is responsible for CARDs exposure. The key result that supports this argument is the charge flipped mutant which is hyperactive despite having the same CHL length as WT CHL.

In response to this comment, on p.6, we added more explanation:

"How does the deletion of 11 aa in CHL destabilize the CARD2:Hel2i interface? Is it a length effect, or does the deletion disrupt some autoinhibitory interactions of the CHL with the helicase? An unstructured CHL of 56 aa should be theoretically 168 Å long (assuming ~ 3 Å per aa), which is four times longer than the distance it needs to span between CARD2 and Hel1 (~ 40 Å). A 33 Å deletion is not long enough to shorten the linker and disrupt the interface if the CHL is truly unstructured. The CHL may have specific, dynamic interactions with the helicase to stabilize the autoinhibitory CARDs conformation."

5. Mutations in the CHL would be expected to elevated baseline RIG-I signaling in the absence of viral RNA, potentially causing autoinflammation. Do any (autoinflammatory or other) disease-associated mutations map to the CHL of RIG-I?

We checked several databases, but to our knowledge, there are no published CHL mutants associated with autoimmune or other diseases. Perhaps point mutations are not sufficient to disrupt the CHL functions. We point this out in the Discussion (p.11).

6. Introduction: where Oates et al (2013) is cited the Alphafold2 structure prediction should also be mentioned. P. 8. Please show the Alfafold2 model of the CHL somewhere.

We now show the Alphafold model in Figure EV1A. The model shows the CHL binding in the RNA channel of the helicase domain.

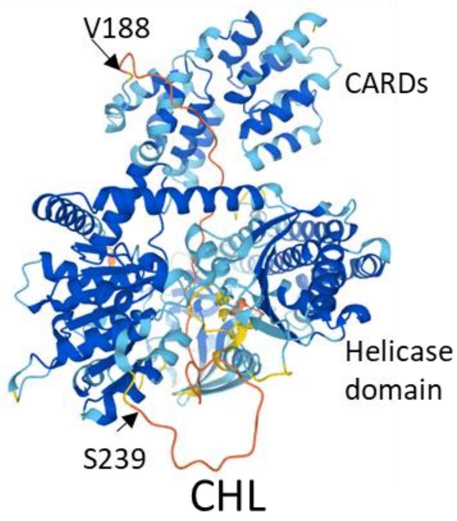

Panel from Figure EV1

A. Alphafold predicted structure of RIG-I shows CHL (in orange) bound in the RNA binding pocket of the helicase domain. The colors correspond to model per-residue confidence (pLDDT): Dark blue, very high (pLDDT > 90), light blue, confident (90 > pLDDT > 70), yellow, low (70 > pLDDT > 50), orange, very low (pLDDT < 50). Regions

7. The Discussion repeats several points already stated or discussed earlier in the paper. The Discussion should be shortened by about one-third (particularly top of p. 9) and be made less repetitive and speculative (particularly bottom of p. 9 and top of p. 10).

We have substantially revised the Discussion. We have made the discussion less speculative by taking out the modeling. We discuss the known proofreading mechanisms of RIG-I. We also compare and discuss the distinct mechanisms of constitutive activation by the Singleton-Merten Syndrome RIG-I mutants and the CHL mutants.

8. Statements such as "The discovery... represents an exciting, new and unique opportunity" (p. 20) should be deleted throughout the text. Readers will form their own opinions and primacy statements are best avoided.

Thank you for the suggestion, we have removed this primacy statement.

9. Typos: P. 3 line 3: change "discovery" to "discovered"; P. 6 last line: delete "more".

We have edited the lines.

10. Please add line numbers and text-embedded figures and figure legends for easier review.

We have provided line numbered, text-embedded version for easier review.

Referee #2 (Report for Author)

This manuscript describes an interesting and new observation that adds to our understanding of how RIG-I distinguishes between 'normal' RNAs and immunostimulatory RNAs: a disordered linker (called here CHL) prevents RNAs from binding to the helicase domain, unless such RNAs are first bound by the CTD. This finding is of high interest to specialists in the field of nucleic acid sensing. Overall, the manuscript is well written and the conclusions are supported by the data. In particular, the HDX-MS and biochemical (binding and ATPase) data are convincing. Unfortunately, functional experiments are limited to a single rather artificial assay. The manuscript would be strengthened (and its wider appeal increased) by further work in this direction, as detailed below.

Major points

1. The HEK293T cells used for the reporter assay do not express high levels of RIG-I and MDA5 at baseline. However, in the 20 hour timeframe of the experiment, transfected pI:C will lead to type I IFN production, which in turn via IFNAR signalling is likely to induce endogenous RIG-I and MDA5 expression. This is an important caveat as pI:C activates both these receptors. As such, the readout at the end of the experiment is a combination of signalling via the overexpressed RIG-I mutants, endogenous RIG-I and endogenous MDA5. This poses difficulties in interpreting the data, in particular because different levels of IFN induction are seen with the mutants; hence, upregulation of the endogenous receptors will likely be different. I suggest to circumvent this issue by using RIG-I KO cells and a more selective RIG-I agonist: 5'PPP dsRNA. The former cells are widely used in the field and the latter is commercially available (Invivogen). Alternatively, if the authors wish to use pI:C, RIG-I + MDA5 double KO cells should be used.

At the request of both reviewers, we have repeated all the cell signaling reporter assays with a RIG-I specific PAMP RNA (blunt-ended 5'ppp ds39 RNA), and replaced the polyIC panels with the 5'ppp RNA data set, in Figures 1 and 3. The use to 5'ppp dsRNA addresses the concern of MDA5 expression in 293T cells.

To address the concern of endogenous RIG-I activation, we have performed the reporter assays in 293T RIG-I KO cells (kindly provided by Dr. Veit Hornung and Dr. Saumendra Sarkar) with two hyperactive mutants with and without PAMP RNA. These data are now included as new panels in Figure 1 (1E & 1F) (shown above in response to reviewer 1, Q.1) and Figure 3 (3E & 3F, see below). It does not appear that there is substantial activation of endogenous RIG-I expression in Δ190-200 or cf200-210 RIG-I transfected cells. Both mutants show close to similar levels of increased basal signaling response in RIG-I KO cells and 293T cells relative to WT RIG-I (7-10

fold). These new experiments fully address reviewer's concerns of endogenous upregulation of RIG-I and MDA5 contributing to the observed signaling responses by the CHL mutants.

See also response to reviewer one comment Q.1.

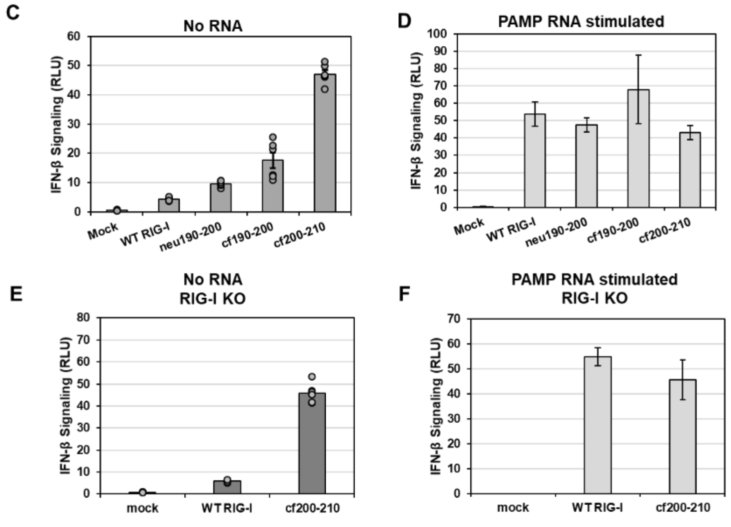

Panels from Figure 3

3C. IFN response measured by reporter assays in HEK293T cells in Mock-transfected, WT RIG-I, a glycine-serine-threonine repeat substitution CHL mutant (neu190-200), and CHL charge flip (cf) mutants under No RNA conditions and reported as Relative Luciferase Units (RLU). Each condition was performed 3 times by 2 independent workers (n=6), and individual trails are plotted. Error bars reflect standard error.

3D. 5'ppp ds39 stimulated IFN response. No RNA signal from panel C was subtracted from the total amount of signaling after RNA transfection. Error bars from 6 trials were calculated by the sum of squares methods from subtracted graphs.

3E. IFN response of Mock, WT RIG-I, and cf200-210 RIG-I performed as in (D) in HEK293T RIG-I KO cells.

3F. 5'ppp ds39 stimulated IFN response of Mock, WT RIG-I, and cf200-210 RIG-I as in (D) in HEK293T RIG-I KO cells.

2. Type I IFN induction should be measured by additional readouts (in addition to the luciferase reporter). These should include: measurement of IFN and ISG mRNA levels by RT-qPCR, measurement of IFNs by ELISA and measurement of ISGs by western blots. Such data would strengthen the conclusions. In addition, p-IRF3 and p-TBK1 western blots may be informative.

To demonstrate that the CHL mutants activate the Type I IFN response pathway, we measured the induction of IFNB, MX1, ISG15, and OAS1 genes using qRT-PCR. Our colleague, Dr. Ping Xie has expertise in this area of research and was kind enough to collaborate with us for these experiments. Both Δ190-200 and cf190-210 RIG-I activated the IFN response genes and pIRF3 expression without PAMP RNA, whereas WT RIG-I required PAMP RNA for activation. These data are now shown in Figure EV1 C-G (see below).

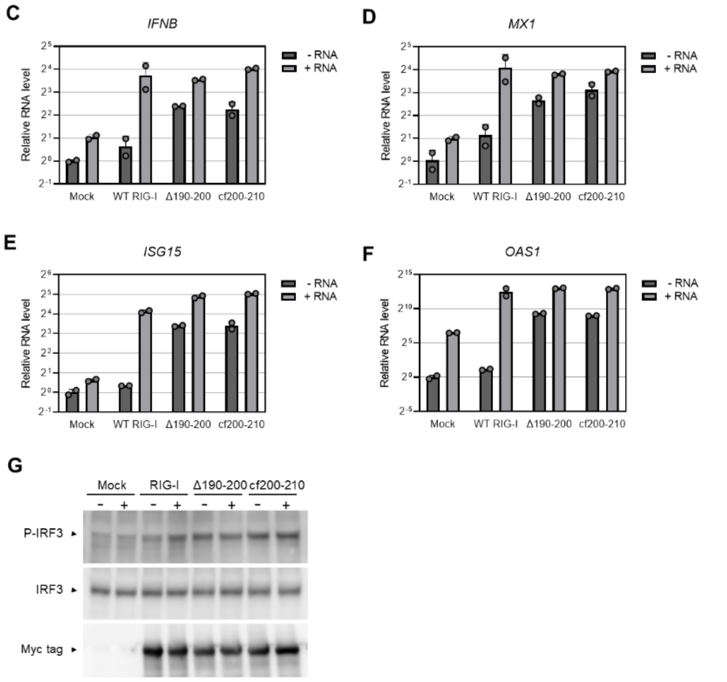

Panels from Figure EV1

C-F, qRT-PCR assays shows the induction of antiviral IFN response genes in the absence and presence of PAMP RNA, 5'ppp ds39. Note the Y-axis is in log scale. Each was performed in duplicate.

G. Western Blot show induction of pIRF3 in RIG-I transfected cells.

3. In addition to using synthetic transfected RNAs as stimuli, the authors should confirm their findings using a virus infection model. Sendai virus would be ideal; it is commercially available (ATCC), is a biosafety level 1 virus, and activates RIG-I specifically.

The reviewer asks us to investigate how the CHL mutants will respond to viral RNA infection, but we are not sure what to expect because the CHL mutants constitutively activate the IFN response pathway, as discussed above in Q. 2. The constitutively activated IFN response could be protective to viral infection. In such a case, the results would be more demonstrative of poor viral infection from high IFN conditions rather than through how CHL mutants recognize viruses. Additionally, the results may depend on the type of virus assayed, as viruses may potentially have undiscovered strategies of inhibiting RIG-I through interactions with CHL. These are interesting lines of experiments that are clearly beyond the scope of this biochemical/biophysical study and should be carried out in future collaboration with a virology lab, where we can test various viruses. We do not have the necessary expertise in this area and it will take a significant amount of time getting the appropriate training to set up the virology assays in the lab.

4. Fig 1D and the statement on page 4 ("We observed a normal PAMP-stimulated signaling response from CHL mutants with Poly I:C") are misleading. For WT RIG-I, pI:C stimulation versus no RNA upregulates the reporter by ca 6-fold (2.5 to 15). However, for the first three mutant RIG-I constructs, this effect is less than 2-fold (15 to 27). Dose-titrations are be needed to properly draw comparative conclusions.

We have repeated all the experiments with the 5'ppp RNA, as requested above, and carried out the No RNA in parallel. To assess the contribution of the 5'ppp RNA to signaling, we subtract the basal signaling response. As shown below panel D from Figure 1, the resulting signaling response of the CHL mutants is not significantly different from the WT RIG-I.

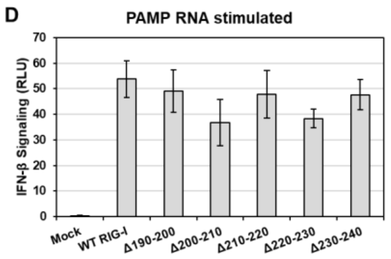

Panel from Figure 1

D. ds39 stimulated IFN response. Note that the PAMP RNA stimulated graph reflects the background; No RNA signal from panel C subtracted from the total amount of signaling after RNA transfection. Error bars from 6 trials were calculated by the sum of squares methods from subtracted graphs.

5. Fig 3A and EV3. Please provide the theoretical pI for the CHLs of all species shown and also for MDA5 CHL.

The pI values are now provided in Figure 3A, and show that the CHL in all RIG-I homologs is almost equally acidic.

6. Fig 4. Please briefly explain the protein purification strategy and provide evidence that all four RIG-I proteins are of equal purity.

We used the published protocol (Jiang et al, 2011) to purify all the RIG-I constructs. The purification strategy is described in Methods (p. 14), and SDS-PAGE of purified RIG-I constructs is shown in Appendix Figure S1A, see below.

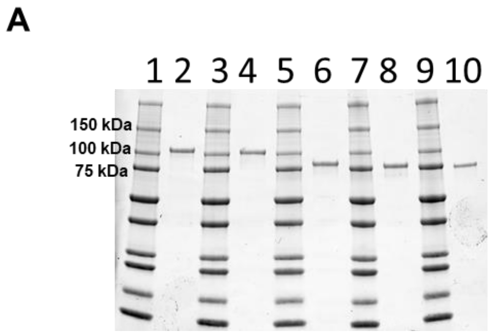

Panel from Appendix Figure S1

(A) SDS PAGE shows the various RIG-I protein constructs. Odd lanes are protein markers. Lane 2: Full length WT RIG-I (107 kDa), lane 4: Δ190-200 RIG-I (105 kDa), lane 6: CHL-Hel-CTD (85 kDa), lane 8: CHL-Hel-CTD cf190-210 (85 kDa), lane

7. The experiments in Figs 5 and EV5 need to be repeated at least once more to allow statistical comparison between different constructs.

As pointed out in the methods, each RNA $K_{D,app}$ value was obtained from three measurements of ATP hydrolysis reactions, repeated twice. We believe that the triplicates and n = 2 allow us to statistically compare the various RNAs and RIG-I constructs. We show the individual RNA binding titrations and the associated standard errors in Figure 5.

8. In Fig 5, it would be informative to include 5-PPP (or 5-PP) RNAs in the analysis as these are the most relevant RIG-I triggers in virus infected cells.

We have now added the 5’ppp dsRNA K_D data to Figure 5 (see response to reviewer 1, Q.1, w) and to Figure 4 (see below). Both data demonstrate that 5’ppp RNA is more competent at outcompeting CHL for RIG-I binding than the non-PAMP RNAs. These results strongly support a role of CHL in RNA proofreading.

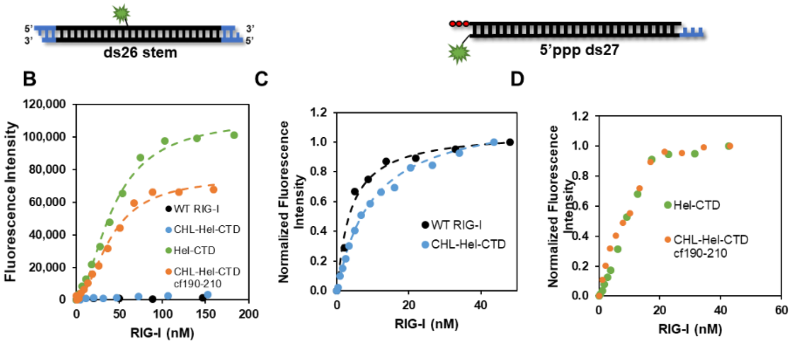

Panels from Figure 4

Fluorimetric titrations show ds26 stem RNA (B) and 5’ppp RNA binding (C, D) to the various RIG-I constructs.

Table summarizes the RNA K_D values and shows the association and dissociation times of each RIG-I construct. No binding or n. b. indicates that stem RNA binding was not observed within 400 nM of titrated RIG-I.

RIG-I Construct	5'ppp RNA K_D (nM)	Stem RNA K_D (nM)	Stem RNA Average Association Time (s)	Stem RNA Average lifetime (s)
WT RIG-I	1.4 ± 0.2	n.b.	5	4
CHL-Hel-CTD	10.5 ± 1.3	n.b.	3	3
Hel-CTD	<1 nM	32.4 ± 1.4	0.04	44
CHL-Hel-CTD cf190-210	<1 nM	22.8 ± 2.4	0.02	4

9. Does LGP2 have a CHL like region?

LGP2 lacks both CHL and CARDs. Sequence alignments position the start of LGP2 at the beginning of the helicase region, which is conserved between LGP2, RIG-I, and MDA5.

10. Are there any naturally occurring mutations in the CHL linker, perhaps linked to diseases such as Singleton-Merten syndrome?

We have not identified any reports demonstrating CHL mutations causing autoimmune disorders. Perhaps multiple mutations are needed to disable the functions of CHL. This point has been added in the Discussion.

Minor points

11. Abstract. First line. Please change 'the first line' to 'a first line' to avoid over-generalisation.

We have made this correction.

12. Page 2. Please provide appropriate references for the statement: "Unlike CTD, the helicase domain lacks RNA specificity and hence can bind non-specific RNAs."

We have modified the sentence and added the appropriate references. *“Unlike CTD, which specifically recognizes blunt 5’ tri- or dephosphorylated RNA ends, the helicase domain generally interacts with the phosphodiester backbone (Jiang et al., 2011; Kowalinski et al., 2011; Luo et*

al., 2011)."

13. The graphs in Fig 1C and EV1 seem identical. I suggest to incorporate the western blot from EV1 into Fig 1. Similarly, I suggest to incorporate the western blot from EV3B (which should be straightened) into Fig 3C/D. Please use consistent nomenclature (presumably neu190-200 and GST 190-200 are the same construct).

Thank you for pointing out the error. Figure 1 and EV1 are substantially revised with the addition of new RIG-I KO signaling data, qRT-PCR data, and Westerns for the reporter assays and pIRF3 expression. We decided that it was important to include the RIG-I KO data in the main figure, which left little space to include the western blots in the main figure. Hence, the qRT-PCR and westerns are shown in Figure EV1.

14. Page 6, figure call-out: Figure 4 has no panel D.

Thank you for pointing this out. Figure 4 is now changed and 4D shows the 5'ppp RNA binding data.

1st Revision - Editorial Decision **3rd Mar 2022**

Thank you for submitting your revised manuscript. We have now received comments from the two initial referees (please see below) and I am pleased to say that they now in principle support publication. Both referees have some remaining issues, which should be addressed in the final revised version of the manuscript. Referee #2 also again asks for titration of the PAMP RNA to show in dose-response experiments that the effects for the different RIG-I constructs are indeed similar. Please add this experiment or provide alternative evidence to support the claim. In addition, I would ask you to address a number of editorial issues that are listed in detail below in a final revised version of the manuscript. Please make all changes to the document provided by our data editors (please see below for more information). Please also include a brief point-by-point response to all referee comments when you submit the revised manuscript.

Referee #1:

The revised version of this manuscript is in general greatly improved, in particular by the addition of binding and signaling assays a more virus-like 5'ppp RNA ligand rather than polyIC, and the use of 293T RIG-I KO cells as requested by Reviewer 2.

Most of the original concerns are fully addressed by the current revision. The following concerns remain:

In the mechanistic model shown in Fig. 6, in the righthand pathway, the length of the two arrows between the autoinhibited stated (top) and the next state with 5'ppp RNA binding should be adjusted to reflect the fact that this pathway is more favorable than the one on the left with self RNA (i.e. the downwards arrow should be longer and upwards arrow shorter for 5'ppp RNA).

A second point regarding Fig. 6 is that on the bottom left the first subunit of RIG-I on 5'pppRNA should be shown binding at the end of the RNA rather than in the middle. In the current model RIG-I first binds at the ends of RNA ligands including PAMPS. Translocation to an internal, stem-binding position only occurs following the ATP-dependent throttling step.

"How does the deletion of 11 aa in CHL destabilize the CARD2:Hel2i interface? Is it a length effect, or does the deletion disrupt some autoinhibitory interactions of the CHL with the helicase? An unstructured CHL of 56 aa should be theoretically 168 Å long (assuming ~ 3 Å per aa), which is four times longer than the distance it needs to span between CARD2 and Hel1 (~ 40 Å). A 33 Å deletion is not long enough to shorten the linker and disrupt the interface if the CHL is truly unstructured. The CHL may have specific, dynamic interactions with the helicase to stabilize the autoinhibitory CARDs conformation."

A remaining concern regarding the statement above (p.6) is that the data presented does not prove conclusively that the CHL is fully unstructured. The H/D exchange experiments show that the CHL can "open up" and expose new surfaces to solvent. While this requires some flexibility or disorder at two or more hinge regions, it remains possible that some of CHL has some stable secondary structure throughout. The best argument so far for the CHL being disordered is actually the new Alphafold model, which shows low confidence score and a random coil conformation for the CHL.

"We now show the Alphafold model in Figure EV1A. The model shows the CHL binding in the RNA channel of the helicase domain."

Although the confidence score for the CHL is low in the AlphaFold model, is an interesting observation that the CHL localized to the RNA binding channel. It might be worth adding a sentence or two in the Discussion expanding on this a little.

Referee #2:

The authors have addressed most of my points. The only exception is that no dose-responses were carried out in the cell-based assay, as requested in major point 4. As such, it remains unclear whether the responses seen upon 'PAMP RNA stimulation' (Fig 1D,F & 3D,F) are actually similar for the different RIG-I constructs, as claimed by the authors. It is possible that differences would be apparent at intermediate (non-saturating) doses of PAMP RNA. I suggest to repeat the experiment in Fig 1F with a dilution series of PAMP RNAs. Additional minor points should also be addressed before publication:

1. Please add size markers to all western blots (Fig EV1B,G and EV3B).
2. The data in Fig 1D,F & 3D,F show reporter signals after RNA stimulation minus basal signals. This is fine, but for transparency the authors should add extra panels showing the data without subtracting basal signals. These panels should show individual data points for comparison with Fig 1C,E & 3C,E.

Referee #1:

The revised version of this manuscript is in general greatly improved, in particular by the addition of binding and signaling assays a more virus-like 5'ppp RNA ligand rather than polyIC, and the use of 293T RIG-I KO cells as requested by Reviewer 2.

Most of the original concerns are fully addressed by the current revision. The following concerns remain:

In the mechanistic model shown in Fig. 6, in the righthand pathway, the length of the two arrows between the autoinhibited stated (top) and the next state with 5'ppp RNA binding should be adjusted to reflect the fact that this pathway is more favorable than the one on the left with self RNA (i.e. the downwards arrow should be longer and upwards arrow shorter for 5'ppp RNA).
Fixed

A second point regarding Fig. 6 is that on the bottom left the first subunit of RIG-I on 5'pppRNA should be shown binding at the end of the RNA rather than in the middle. In the current model RIG-I first binds at the ends of RNA ligands including PAMPS. Translocation to an internal, stem-binding position only occurs following the ATP-dependent throttling step.
The reviewer probably means the bottom right. Nevertheless, to distinguish self RNA from PAMP RNA (other than the color), we have added dashes to each 5' and 3' end to indicate the RNA continues beyond the schematic. The bottom right portion molecule of PAMP RNA has been amended to show translocation (arrow added) and loading of the second molecule of RIG-I.

"How does the deletion of 11 aa in CHL destabilize the CARD2:Hel2i interface? Is it a length effect, or does the deletion disrupt some autoinhibitory interactions of the CHL with the helicase? An unstructured CHL of 56 aa should be theoretically 168 Å long (assuming ~ 3 Å per aa), which is four times longer than the distance it needs to span between CARD2 and Hel1 (~ 40 Å). A 33 Å deletion is not long enough to shorten the linker and disrupt the interface if the CHL is truly unstructured. The CHL may have specific, dynamic interactions with the helicase to stabilize the autoinhibitory CARDs conformation."

A remaining concern regarding the statement above (p.6) is that the data presented does not prove conclusively that the CHL is fully unstructured. The H/D exchange experiments show that the CHL can "open up" and expose new surfaces to solvent. While this requires some flexibility or disorder at two or more hinge regions, it remains possible that some of CHL has some stable secondary structure throughout. The best argument so far for the CHL being disordered is actually the new Alphafold model, which shows low confidence score and a random coil conformation for the CHL.

Thank you for this comment. We have added this comment mentioning Alphafold's disorder prediction to the paper. We have also added a graph showing the confidence scores to make it clearer that the CHL is predicted to be disordered (Figure EV1B).

"We now show the Alphafold model in Figure EV1A. The model shows the CHL binding in the

RNA channel of the helicase domain."

Although the confidence score for the CHL is low in the AlphaFold model, is an interesting observation that the CHL localized to the RNA binding channel. It might be worth adding a sentence or two in the Discussion expanding on this a little.
Thank you for this comment, we have added lines discussing this.

Referee #2:

The authors have addressed most of my points. The only exception is that no dose-responses were carried out in the cell-based assay, as requested in major point 4. As such, it remains unclear whether the responses seen upon 'PAMP RNA stimulation' (Fig 1D,F & 3D,F) are actually similar for the different RIG-I constructs, as claimed by the authors. It is possible that differences would be apparent at intermediate (non-saturating) doses of PAMP RNA. I suggest to repeat the experiment in Fig 1F with a dilution series of PAMP RNAs. Additional minor points should also be addressed before publication:
A dose titration for the CHL deletion constructs has been added (Fig 1D-E).

1. Please add size markers to all western blots (Fig EV1B,G and EV3B).
Added, additionally we have updated the raw data on BioStudies to show Western Blots with molecular weight markers.

2. The data in Fig 1D,F & 3D,F show reporter signals after RNA stimulation minus basal signals. This is fine, but for transparency the authors should add extra panels showing the data without subtracting basal signals. These panels should show individual data points for comparison with Fig 1C,E & 3C,E.
We now show the total values without subtracting basal signal for each cell signal assay.

2nd Revision - Editorial Decision 4th Apr 2022

Thank you again for submitting the final revised version of your manuscript and addressing the remaining points. I am pleased to inform you that we have now accepted it for publication in The EMBO Journal.

EMBO Press Author Checklist

Corresponding Author Name: Smita Patel
Journal Submitted to: EMBO J
Manuscript Number: EMBOJ-2021-109782

USEFUL LINKS FOR COMPLETING THIS FORM
The EMBO Journal - Author Guidelines
EMBO Reports - Author Guidelines
Molecular Systems Biology - Author Guidelines
EMBO Molecular Medicine - Author Guidelines

Reporting Checklist for Life Science Articles (updated January 2022)
This checklist is adapted from Materials Design Analysis Reporting (MDAR) Checklist for Authors. MDAR establishes a minimum set of requirements in transparent reporting in the life sciences (see Statement of Task: 10.31222/osf.io/9sm4x). Please follow the journal's guidelines in preparing your manuscript.
Please note that a copy of this checklist will be published alongside your article.

Abridged guidelines for figures
1. Data
The data shown in figures should satisfy the following conditions:
- the data were obtained and processed according to the field's best practice and are presented to reflect the results of the experiments in an accurate and unbiased manner.
- ideally, figure panels should include only measurements that are directly comparable to each other and obtained with the same assay.
- plots include clearly labeled error bars for independent experiments and sample sizes. Unless justified, error bars should not be shown for technical replicates.
- if n<5, the individual data points from each experiment should be plotted. Any statistical test employed should be justified.
- Source Data should be included to report the data underlying figures according to the guidelines set out in the authorship guidelines on Data Presentation.

2. Captions
Each figure caption should contain the following information, for each panel where they are relevant:
- a specification of the experimental system investigated (eg cell line, species name).
- the assay(s) and method(s) used to carry out the reported observations and measurements.
- an explicit mention of the biological and chemical entity(ies) that are being measured.
- an explicit mention of the biological and chemical entity(ies) that are altered/varied/perturbed in a controlled manner.
- the exact sample size (n) for each experimental group/condition, given as a number, not a range;
- a description of the sample collection allowing the reader to understand whether the samples represent technical or biological replicates (including how many animals, litters, cultures, etc.).
- a statement of how many times the experiment shown was independently replicated in the laboratory.
- definitions of statistical methods and measures:
  - common tests, such as t-test (please specify whether paired vs. unpaired), simple χ2 tests, Wilcoxon and Mann-Whitney tests, can be unambiguously identified by name only, but more complex techniques should be described in the methods section;
  - are tests one-sided or two-sided?
  - are there adjustments for multiple comparisons?
  - exact statistical test results, e.g., P values = x but not P values < x;
  - definition of 'center values' as median or average;
  - definition of error bars as s.d. or s.e.m.

Please complete ALL of the questions below.
Select "Not Applicable" only when the requested information is not relevant for your study.

Materials

**Newly Created Materials**	**Information included in the manuscript?**	**In which section is the information available?** (Reagents and Tools Table, Materials and Methods, Figures, Data Availability Section)
New materials and reagents need to be available; do any restrictions apply?	Not Applicable	

**Antibodies**	**Information included in the manuscript?**	**In which section is the information available?** (Reagents and Tools Table, Materials and Methods, Figures, Data Availability Section)
For **antibodies** provide the following information:<br>- Commercial antibodies: RRID (if possible) or supplier name, catalogue number and or/clone number<br>- Non-commercial: RRID or citation	Yes	Reagents and Tools Table

**DNA and RNA sequences**	**Information included in the manuscript?**	**In which section is the information available?** (Reagents and Tools Table, Materials and Methods, Figures, Data Availability Section)
**Short novel DNA or RNA including primers, probes**: provide the sequences.	Yes	Reagents and Tools Table

**Cell materials**	**Information included in the manuscript?**	**In which section is the information available?** (Reagents and Tools Table, Materials and Methods, Figures, Data Availability Section)
**Cell lines:** Provide species information, strain. Provide accession number in repository **OR** supplier name, catalog number, clone number, and/**OR** RRID.	Yes	Devakar et al, 2016, Materials and Methods
**Primary cultures:** Provide species, strain, sex of origin, genetic modification status.	Yes	Devakar et al, 2016, Materials and Methods
Report if the cell lines were recently **authenticated** (e.g., by STR profiling) and tested for mycoplasma contamination.	Not Applicable	

**Experimental animals**	**Information included in the manuscript?**	**In which section is the information available?** (Reagents and Tools Table, Materials and Methods, Figures, Data Availability Section)
**Laboratory animals or Model organisms:** Provide species, strain, sex, age, genetic modification status. Provide accession number in repository **OR** supplier name, catalog number, clone number, **OR** RRID.	Not Applicable	
**Animal observed in or captured from the field:** Provide species, sex, and age where possible.	Not Applicable	
Please detail **housing and husbandry conditions**.	Not Applicable	

**Plants and microbes**	**Information included in the manuscript?**	**In which section is the information available?** (Reagents and Tools Table, Materials and Methods, Figures, Data Availability Section)
**Plants:** provide species and strain, ecotype and cultivar where relevant, unique accession number if available, and source (including location for collected wild specimens).	Not Applicable	
**Microbes:** provide species and strain, unique accession number if available, and source.	Not Applicable	

**Human research participants**	**Information included in the manuscript?**	**In which section is the information available?** (Reagents and Tools Table, Materials and Methods, Figures, Data Availability Section)
If collected and within the bounds of privacy constraints report on age, sex and gender or ethnicity for all study participants.	Not Applicable	

**Core facilities**	**Information included in the manuscript?**	**In which section is the information available?** (Reagents and Tools Table, Materials and Methods, Figures, Data Availability Section)
If your work benefited from core facilities, was their service mentioned in the acknowledgments section?	Not Applicable	

Design

Study protocol	Information included in the manuscript?	In which section is the information available? (Reagents and Tools Table, Materials and Methods, Figures, Data Availability Section)
If study protocol has been **pre-registered, provide DOI in the manuscript**. For clinical trials, provide the trial registration number **OR** cite DOI.	Not Applicable	
Report the **clinical trial registration number** (at ClinicalTrials.gov or equivalent), where applicable.	Not Applicable	

Laboratory protocol	Information included in the manuscript?	In which section is the information available? (Reagents and Tools Table, Materials and Methods, Figures, Data Availability Section)
Provide DOI OR other citation details if **external detailed step-by-step protocols** are available.	Not Applicable	

Experimental study design and statistics	Information included in the manuscript?	In which section is the information available? (Reagents and Tools Table, Materials and Methods, Figures, Data Availability Section)
Include a statement about **sample size** estimate even if no statistical methods were used.	Yes	Materials and Methods
Were any steps taken to minimize the effects of subjective bias when allocating animals/samples to treatment (e.g. **randomization procedure**)? If yes, have they been described?	Not Applicable	
Include a statement about **blinding** even if no blinding was done.	Not Applicable	
Describe **inclusion/exclusion criteria** if samples or animals were excluded from the analysis. Were the criteria pre-established?<br><br>If sample or data points were omitted from analysis, report if this was due to attrition or intentional exclusion and provide justification.	Not Applicable	
For every figure, are **statistical tests** justified as appropriate? Do the data meet the assumptions of the tests (e.g., normal distribution)? Describe any methods used to assess it. Is there an estimate of variation within each group of data? Is the variance similar between the groups that are being statistically compared?	Yes	Figures

Sample definition and in-laboratory replication	Information included in the manuscript?	In which section is the information available? (Reagents and Tools Table, Materials and Methods, Figures, Data Availability Section)
In the figure legends: state number of times the experiment was **replicated** in laboratory.	Yes	Figures
In the figure legends: define whether data describe **technical or biological replicates**.	Yes	Figures

Ethics

Ethics	Information included in the manuscript?	In which section is the information available? (Reagents and Tools Table, Materials and Methods, Figures, Data Availability Section)
Studies involving **human participants**: State details of **authority granting ethics approval** (IRB or equivalent committee(s), provide reference number for approval.	Not Applicable	
Studies involving **human participants**: Include a statement confirming that **informed consent** was obtained from all subjects and that the experiments conformed to the principles set out in the WMA Declaration of Helsinki and the Department of Health and Human Services Belmont Report.	Not Applicable	
Studies involving **human participants:** For publication of **patient photos**, include a statement confirming that consent to publish was obtained.	Not Applicable	
Studies involving experimental **animals**: State details of **authority granting ethics approva**l (IRB or equivalent committee(s), provide reference number for approval. Include a statement of compliance with ethical regulations.	Not Applicable	
Studies involving **specimen and field samples:** State if relevant **permits** obtained, provide details of authority approving study; if none were required, explain why.	Not Applicable	

Dual Use Research of Concern (DURC)	Information included in the manuscript?	In which section is the information available? (Reagents and Tools Table, Materials and Methods, Figures, Data Availability Section)
Could your study fall under dual use research restrictions? Please check biosecurity documents and list of **select agents and toxins** (CDC): https://www.selectagents.gov/sat/list.htm	Not Applicable	
If you used a select agent, is the security level of the lab appropriate and reported in the manuscript?	Not Applicable	
If a study is subject to dual use research of concern regulations, is the name of the **authority granting approval and reference number** for the regulatory approval provided in the manuscript?	Not Applicable	

Reporting

The MDAR framework recommends adoption of discipline-specific guidelines, established and endorsed through community initiatives. Journals have their own policy about requiring specific guidelines and recommendations to complement MDAR.

Adherence to community standards	Information included in the manuscript?	In which section is the information available? (Reagents and Tools Table, Materials and Methods, Figures, Data Availability Section)
State if relevant guidelines or checklists (e.g., **ICMJE, MIBBI, ARRIVE, PRISMA**) have been followed or provided.	Not Applicable	
For **tumor marker prognostic studies,** we recommend that you follow the **REMARK** reporting guidelines (see link list at top right). See author guidelines, under 'Reporting Guidelines'. Please confirm you have followed these guidelines.	Not Applicable	
For **phase II and III randomized controlled trials**, please refer to the **CONSORT** flow diagram (see link list at top right) and submit the CONSORT checklist (see link list at top right) with your submission. See author guidelines, under 'Reporting Guidelines'. Please confirm you have submitted this list.	Not Applicable	

Data Availability

Data availability	Information included in the manuscript?	In which section is the information available? (Reagents and Tools Table, Materials and Methods, Figures, Data Availability Section)
Have **primary datasets** been deposited according to the journal's guidelines (see 'Data Deposition' section) and the respective accession numbers provided in the Data Availability Section?	Not Applicable	
Were **human clinical and genomic datasets** deposited in a public access-controlled repository in accordance to ethical obligations to the patients and to the applicable consent agreement?	Not Applicable	
Are **computational models** that are central and integral to a study available without restrictions in a machine-readable form? Were the relevant accession numbers or links provided?	Not Applicable	
If publicly available data were reused, provide the respective **data citations in the reference list**.	Not Applicable	
